# An enhanced ant colony optimizer with Cauchy-Gaussian fusion and novel movement strategy for multi-threshold COVID-19 X-ray image segmentation

**DOI:** 10.3389/fninf.2023.1126783

**Published:** 2023-03-17

**Authors:** Xiuzhi Zhao, Lei Liu, Ali Asghar Heidari, Yi Chen, Benedict Jun Ma, Huiling Chen, Shichao Quan

**Affiliations:** ^1^College of Artificial Intelligence, Zhejiang Industry & Trade Vocational College, Wenzhou, Zhejiang, China; ^2^College of Computer Science, Sichuan University, Chengdu, Sichuan, China; ^3^School of Surveying and Geospatial Engineering, College of Engineering, University of Tehran, Tehran, Iran; ^4^Institute of Big Data and Information Technology, Wenzhou University, Wenzhou, China; ^5^Department of Industrial and Manufacturing Systems Engineering, The University of Hong Kong, Hong Kong, Hong Kong SAR, China; ^6^Department of Big Data in Health Science, The First Affiliated Hospital of Wenzhou Medical University, Wenzhou, China; ^7^Key Laboratory of Intelligent Treatment and Life Support for Critical Diseases of Zhejiang Province, Wenzhou, China; ^8^Zhejiang Engineering Research Center for Hospital Emergency and Process Digitization, Wenzhou, China

**Keywords:** ant colony optimization, continuous optimization, swarm intelligence, 2D Kapur’s entropy, multi-threshold image segmentation

## Abstract

The novel coronavirus pneumonia (COVID-19) is a respiratory disease of great concern in terms of its dissemination and severity, for which X-ray imaging-based diagnosis is one of the effective complementary diagnostic methods. It is essential to be able to separate and identify lesions from their pathology images regardless of the computer-aided diagnosis techniques. Therefore, image segmentation in the pre-processing stage of COVID-19 pathology images would be more helpful for effective analysis. In this paper, to achieve highly effective pre-processing of COVID-19 pathological images by using multi-threshold image segmentation (MIS), an enhanced version of ant colony optimization for continuous domains (MGACO) is first proposed. In MGACO, not only a new move strategy is introduced, but also the Cauchy-Gaussian fusion strategy is incorporated. It has been accelerated in terms of convergence speed and has significantly enhanced its ability to jump out of the local optimum. Furthermore, an MIS method (MGACO-MIS) based on MGACO is developed, where it applies the non-local means, 2D histogram as the basis, and employs 2D Kapur’s entropy as the fitness function. To demonstrate the performance of MGACO, we qualitatively analyze it in detail and compare it with other peers on 30 benchmark functions from IEEE CEC2014, which proves that it has a stronger capability of solving problems over the original ant colony optimization for continuous domains. To verify the segmentation effect of MGACO-MIS, we conducted a comparison experiment with eight other similar segmentation methods based on real pathology images of COVID-19 at different threshold levels. The final evaluation and analysis results fully demonstrate that the developed MGACO-MIS is sufficient to obtain high-quality segmentation results in the COVID-19 image segmentation and has stronger adaptability to different threshold levels than other methods. Therefore, it has been well-proven that MGACO is an excellent swarm intelligence optimization algorithm, and MGACO-MIS is also an excellent segmentation method.

## 1. Introduction

The outbreak of novel coronavirus pneumonia (COVID-19) in late 2019 and early 2020 is an emerging acute respiratory disease, and diagnosis based on X-ray images is one of the effective complementary diagnostic methods for COVID-19. In clinical practice, imaging devices, such as chest X-ray and chest CT, can significantly help to screen for COVID-19 (Bernheim et al., 2020; Kanne, 2020; Xie et al., 2020). COVID-19 causes severe respiratory symptoms, and most patients diagnosed with COVID-19 have been found to have abnormal chest X-ray images in clinical management, and the X-ray imaging presentation is varied. The diagnostic process is laborious and time-consuming if it is based on the experience of pathologists. As a result, computer-aided diagnostic techniques are a key pre-condition for further X-ray image analysis, which is vital for early illness diagnosis and functional analysis of small parts of the lung, such as lung density analysis, airway analysis, and pulmonary septum mechanics. A weakly supervised deep active learning system called COVID-AL was suggested by Wu et al. (2021a) to diagnose COVID-19 using CT images and patient-level labels. In their research, Shorfuzzaman and Hossain (2021) suggested an artificial intelligence (AI) method based on deep meta-learning to speed up the interpretation of chest X-ray images in the automated detection of COVID-19 patients. Hu et al. (2021) analytical model for COVID-19 diagnosis and therapy is built on complex networks and machine learning methods. For the automated diagnosis of COVID-19, Chen et al. (2021) introduced a new deep learning technique that only needs a small number of training data. Jin et al. (2021) presented a self-correction approach based on domain adaptation for COVID-19 infection segmentation on CT images. Abdel-Basset et al. (2020) suggested a new hybrid solution for COVID-19 chest X-ray pictures based on the thresholding technique by mixing a slime mold algorithm with the whale optimization algorithm. It is also important to be able to separate and identify lesions from COVID-19 pathology images regardless of the computer-aided diagnosis techniques. Therefore, the introduction of image segmentation in the pre-processing stage of COVID-19 pathology images would be more helpful for effective analysis of COVID-19 pathology images.

In recent years, multi-threshold image segmentation (MIS) method is playing an increasingly important role in medical image processing, which can achieve highly effective pre-processing of pathological images and help to promote the development of related medical aid diagnosis technologies (Manikandan et al., 2014; Li et al., 2017; Kotte et al., 2018; Tarkhaneh and Shen, 2019; Hilali-Jaghdam et al., 2020). Medical information systems are a key requirement in the current medical sciences (Ban et al., 2022; Qin et al., 2022). Therefore, in recent years, many scholars have carried out research on MIS techniques based on swarm intelligence optimization algorithms. Zhao et al. (2021a) developed an image segmentation model that was proved by conducting an experiment on a standard image set, in which a modified continuous version of the ant colony optimizer was its core. Verma et al. (2021) presented a hybrid algorithm by combining the excellent features of fuzzy c-means and particle swarm optimization (PSO) to achieve MIS, which was proved by an experiment on a triangular dataset and publicly available real brain datasets. Zhao et al. (2021b) proposed an MIS method based on an improved continuous ant colony optimization algorithm, which was verified by a test on a standard image set. Rakesh and Mahesh (2021) proposed a cuckoo search optimization technique to achieve the initial segmentation of the lung portion. Vaze et al. (2021) developed quantum entanglement-inspired PSO, which was applied to MIS and employed eight gray-scale standard test pictures. In order to resolve pre-treatment and post-treatment organ segmentation difficulties, Chakraborty et al. (2021) proposed a novel dynamically learned PSO-based neighborhood-influenced fuzzy c-means clustering technique. Aranguren et al. (2021) reported an MIS methodology for magnetic resonance brain imaging segmentation based on the LSHADE algorithm. Huang et al. (2021) used the fruit fly optimization algorithm (FOA) for OTSU segmentation, resulting in an FOA-OTSU segmentation method that employed a classical picture to validate the proposed segmentation technique.

Xing (2020) proposed and evaluated a MIS method based on improved Emperor Penguin Optimizer, utilizing Berkeley pictures, satellite images, and plant canopy pictures. Dinkar et al. (2021) suggested a modified equilibrium optimizer for segmenting gray-scale images with MIS and tested their proposed technique by utilizing some standard images. Wu B. et al. (2020) proposed a MIS approach using an improved teaching–learning-based optimization algorithm, which is successfully applied in casting X-ray image segmentation for MIS. The Harris Hawks optimization (HHO) technique was used by Rodríguez-Esparza et al. (2020) to suggest an effective solution for MIS and test it on specific digital mammography medical pictures. Abd Elaziz et al. (2021) presented a modified version of the manta ray foraging optimizer algorithm to deal with MIS problems, which is proved by a test on some standard images. Radha and Gopalakrishnan (2020) provided an intelligent fuzzy-level set approach for medical picture segmentation and an overall search proficiency of enhanced quantum PSO.

Kalyani et al. (2020) devised and evaluated an efficient exchange market algorithm for image segmentation utilizing the minimal cross-entropy thresholding approach on brain pictures with varying threshold values. Elaziz et al. (2020) introduced an improved Harris hawks optimizer for global optimization and determining the best threshold values for MIS situations. With the help of a greedy snake model and fuzzy C-means optimization, Sheela and Suganthi (2019) suggested an effective automated brain tumor segmentation. A brand-new multi-objective optimization strategy for segmenting magnetic resonance imaging of the human brain was introduced by Pham et al. (2019). Wang et al. (2022) developed a unique, consistent perception generative adversarial network for semi-supervised stroke lesion segmentation that may eliminate the need for wholly labeled data. Authors in You et al. (2022) proposed fine perceptive generative adversarial networks, which are built to deal with the low-frequency and high-frequency components of MR images separately and concurrently by using the divide-and-conquer strategy (Wang et al., 2022) presented a 3D end-to-end synthesis network dubbed Bidirectional Mapping Generative Adversarial Networks for brain magnetic resonance imaging and positron emission tomography synthesis and segmentation. Through the study of various MIS methods proposed in recent years, it is found that most MIS techniques are based on swarm intelligence optimization algorithms. In the MIS method based on swarm intelligence algorithm, the swarm intelligence optimization algorithm is the core of the segmentation, and the suitability of the optimization algorithm directly determines the good or bad segmentation effect. Due to the characteristics of the swarm intelligence algorithm itself, it is difficult for the algorithm to converge to the optimal solution of the problem in complex practical problems, so the segmentation method based on the optimization algorithm still has more room for improvement to a certain extent, and the image segmentation effect can be improved as much as possible by proposing a suitable optimization algorithm.

In terms of swarm intelligence optimization algorithms, not only a series of basic algorithms have been proposed, such as multi-verse optimizer (MVO) (Mirjalili et al., 2016), ant colony optimization for continuous domains (ACOR) (Socha and Dorigo, 2008), bat-inspired algorithm (BA) (Yang, 2010), different evolution (DE) (Storn and Price, 1997), firefly algorithm (FA) (Yang, 2009), gray wolf optimization (GWO) (Mirjalili et al., 2014), moth-flame optimization (MFO) (Mirjalili, 2015), PSO (Kennedy and Eberhart, 1995), sine cosine algorithm (SCA) (Mirjalili, 2016), slime mold algorithm (Chen H. et al., 2022), whale optimizer (WOA) (Mirjalili and Lewis, 2016), and HHO (Heidari et al., 2019c), but also many variant versions based on the basic algorithms have been proposed by many scholars, such as boosted GWO (OBLGWO) (Heidari et al., 2019a), opposition-based SCA (OBSCA) (Abd Elaziz et al., 2017), ant colony optimizer with random spare strategy and chaotic intensification strategy (RCACO) (Zhao et al., 2021a), hybrid bat algorithm (RCBA) (Liang et al., 2018), A-C parametric WOA (ACWOA) (Elhosseini et al., 2019), enhanced WOA with associative learning (BMWOA) (Heidari et al., 2019b), bat algorithm based on collaborative and dynamic learning of opposite population (CDLOBA) (Yong et al., 2018), enhanced whale optimizer with new communication mechanism and biogeography-based optimization (EWOA) (Tu et al., 2021), hybridized gray wolf optimization (HGWO) (Zhu et al., 2015), modified SCA (m_SCA) (Qu et al., 2018), biogeography-based learning PSO (BLPSO) (Chen X. et al., 2017), comprehensive learning PSO (CLPSO) (Liang et al., 2006), enhanced GWO with a new hierarchical structure (IGWO) (Cai et al., 2019), improved WOA (IWOA) (Tubishat et al., 2019) and so on. Swarm intelligence algorithms have been applied to solve many problems such as bankruptcy prediction (Zhang et al., 2021), feature selection (Xue et al., 2019, 2022a; Liu et al., 2022b), economic emission dispatch (Dong et al., 2021), dynamic multiobjective optimization (Yu et al., 2022), constrained multiobjective optimization (Liang et al., 2022), global optimization (Deng et al., 2022), large-scale complex optimization (Huang et al., 2023), and feed-forward neural networks (Xue et al., 2022b).

Among them, ACOR is an algorithm proposed by Socha and Dorigo (2008) to apply it to solve problems in the continuous domain, which not only retains the original ACO characteristics but also overcomes the drawback that it can only be applied to discrete problems. Therefore, ACOR has a high research value and has been studied by many scholars. By combining the basic ant colony optimizer with chaotic intensification and random spare strategies, Zhao et al. (2021a) presented a unique variation of ACOR and put it to the test using 30 benchmark functions. Kumar et al. (2018) proposed a new ant colony optimization algorithm that incorporated Laplace distribution-based interaction scheme among the ants. A hybrid form of ant colony optimization for continuous domains was presented by Karakonstantis and Vlachos (2018), which can deal with continuous optimization issues with or without constraints. An innovative population-based elite-mixed continuous ant colony optimization with central initialization was developed by Chen and Shen (2018). In order to solve issues involving continuous variables, Wu et al. (2017) suggested a dynamic-edge ant colony systems technique that could produce edges between two nodes and provide pheromone measurements in a continuous solution space. A cooperative continuous ant colony optimization approach was put out by Juang et al. (2013) and used to solve accuracy-focused design issues for fuzzy systems. A novel classifier based on ant colony optimization in continuous domains was proposed by Shahraki and Zahiri (2017), and it was utilized to identify the decision hyperplanes between various classes. Falcon-Cardona and Coello (2017) developed an ant colony optimizer-based multi-objective ant colony optimizer for continuous search spaces. Chen Z. et al. (2017) published a robust ant colony optimization for continuous functions that employed self-adaptive approaches for domain modification, pheromone increment, domain division, and ant size. Zhang et al. (2016) introduced a novel hybrid ant colony optimization and PSO technique to solve the slow convergence of the ant colony optimization strategy for continuous domain difficulties. In order to solve a continuous space optimization problem, Huang (2016) suggested an ant colony algorithm that enhanced the fundamental algorithm in terms of ant colony initialization, information density function, distribution methods, the direction of ant colony motion, and other areas. Three crossover approaches are used in the enhanced continuous ant colony optimization with crossover operators developed by Chen and Wang (2017) to provide a new set of probability density functions. By enhancing the selection mechanism and incorporating horizontal and vertical crossover search into the ant colony optimization, Zhao et al. (2021b) proposed an improved ant colony optimization algorithm. This algorithm was tested in a series of comparative experiments using 30 benchmark functions from IEEE CEC 2014. Wu et al. (2019) developed an effective optimization technique for local search operations and established a multimodal continuous ant colony optimization algorithm. Gao (2015) proposed an unique immunological continuous ant colony approach to improve the efficacy of the standard evolutionary algorithm. Liu et al. (2014) provided an ant colony foraging distribution model for continuous domains based on their study of the interplay between position distribution and food supply in the process of ant colony foraging.

The multiple study studies above discovered that when ACOR is used to tackle real issues, it is more prone to slip into local optimality, resulting in disappointing outcomes. Furthermore, when applying ACOR to MIS, we discovered a similar issue: ACOR is more prone to falling into local optima, resulting in pictures that are as prone to falling into local optima during the segmentation process, leading in worse segmentation outcomes. Therefore, we propose an enhanced version of ACOR (MGACO) to apply ACOR to MIS to avoid falling into local optima as much as possible and obtain high-quality segmentation results. In MGACO, not only a novel movement strategy is introduced, but also a Cauchy-Gaussian fusion strategy is incorporated after this movement strategy. The introduction of the novel movement strategy and the Cauchy-Gaussian fusion strategy has led to a speedup in the convergence rate of MGACO and has resulted in a significant enhancement in the ability of MGACO to jump out of the local optimum. To demonstrate these core advantages of MGACO, we have used the 30 benchmark functions of IEEE CEC2014 as a basis for qualitatively analyzing MGACO and ACOR in detail and comparing MGACO with ten traditional basic algorithms and ten variants of basic-based algorithms, respectively, in our experiments. We conducted a detailed statistical analysis of the obtained experimental results using mean, variance, Wilcoxon signed-rank test (García et al., 2010), and Friedman test (Derrac et al., 2011), all of which fully demonstrate that MGACO not only has a certain acceleration in convergence speed but also its ability to jump out of the local optimum is significantly enhanced. In addition, we developed an MIS method based on MGACO (MGACO-MIS) using the non-local means, 2D histogram as the basis, and 2D Kapur’s entropy as the fitness function. Intending to demonstrate that MGACO can obtain high-quality segmentation results during image segmentation, we conducted a comparison experiment between MGACO-MIS and eight other similar segmentation methods based on real pathology images of COVID-19 at threshold levels of 4, 5, 6, 15, 20, and 25, where 4, 5, and 6 represent low threshold levels and 15, 20, and 25 represent high threshold levels. First of all, three common and accepted evaluation methods were evaluated for obtaining segmentation results, including Peak Signal Noise Ratio (PSNR) (Huynh-Thu and Ghanbari, 2008), Structural Similarity Index (SSIM) (Zhou et al., 2004), and Feature Similarity Index (FSIM) (Zhang et al., 2011). Secondly, the evaluation results were further analyzed in detail using the mean, variance, Wilcoxon signed-rank test (Chen H. et al., 2022), and Friedman test (Mirjalili and Lewis, 2016). Finally, the evaluation and analysis result sufficiently demonstrate that the proposed MGACO-MIS can obtain high-quality segmentation results when performing image segmentation.

In summary, the main contributions of this paper are in the following aspects.

λ Taking ACOR as a basis, an enhanced version of ACOR combining a new mobility strategy and a Cauchy-Gaussian fusion strategy is proposed, called MGACO.

λ Based on IEEE CEC2014, the qualitative analysis of MGACO and ACOR is conducted, and MGACO is compared with many similar methods, which fully demonstrate the core advantages of MGACO.

λ An MIS based on MGACO, called MGACO-MIS, is developed using non-local means, 2D histogram, and 2D Kapur’s entropy as the fitness function.

λ Based on the real pathological images of COVID-19, the comparison experiments between MGACO-MIS and similar methods have been conducted at several threshold levels, and the segmentation effect of MGACO-MIS has been well demonstrated.

The other sections of this paper are organized as follows: Section “2. An overview of ACOR” provides a brief review of the ACOR basic theory. Section “3. Proposed MGACO” describes the proposed MGACO based on the new movement strategy and the Cauchy-Gaussian fusion strategy in detail. The experimental results and analysis are presented in Section “5. Experiments and results.” In Section “6. Discussion,” the main work of this paper is discussed, and Section “7. Conclusion and future works” gives a summary of the whole paper and future research directions.

## 2. An overview of ACOR

In 2008, Socha and Dorigo (2008) proposed ACOR, which directly extends the ACO in the discrete domain to the continuous domain by treating the solutions in the solution archive as its pheromone, where the update of the pheromone is done by updating the solutions in the archive.

In ACOR, the archive stores *k* solutions *x*_*l*_(*l*=1,…,*k*), the fitness value of each solution, and the weight value corresponding to each solution, where each solution has *n* dimensions, which also represent the dimensions of the problem.$x_{l}=\left(x_{l}^{1},x_{l}^{2},\ldots ,x_{l}^{i},\ldots ,x_{l}^{n}\right)$ denotes a solution of the problem, *f*(*x*_*l*_) is its corresponding fitness value, and *w_l_* is its corresponding weight value.

These *k* solutions are initially generated randomly, and then the solutions *x_l_* are ordered according to the function value *f*(*x*_*l*_) of the solutions.

**FIGURE 1:**
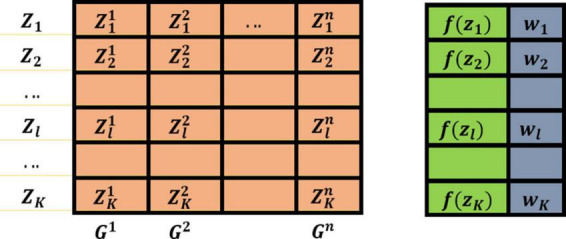
The constructed archive in ACOR.

Therefore, if the fitness value corresponding to each solution satisfies *f*(*s*_1_)≤…*f*(*s*_*l*_)≤…*f*(*s*_*k*_), then the weight *w_l_* corresponding to each solution satisfies *w*_1_≥…*w*_*l*_≥…*w*_*k*_. Based on the above principle, the ACOR archive can be shown in Figure 1.

Therefore, the main mathematical model of the Gaussian kernel function corresponding to ACOR is shown in Eq. (1).


(1) Gi⁢(x)=∑l=1kwl⁢gli⁢(x)=∑l=1kwl⁢1σli⁢2⁢π⁢e-(x-μli)22⁢σli2

where *w*={*w*_1_,…,*w*_*l*_,…,*w*_*k*_} is the weight vector corresponding to each solution, and *w_l_* can be calculated by Eq. (2), $\sigma^{i}=\left\{\sigma_{1}^{i},\ldots ,\sigma_{l}^{i},\ldots ,\sigma_{k}^{i}\right\}$ is the vector of standard deviation corresponding to each solution, and $\sigma_{l}^{i}$ can be calculated from Eq. (3), $\mu^{i}=\left\{\mu_{1}^{i},\ldots ,\mu_{l}^{i},\ldots ,\mu_{k}^{i}\right\}$ is the vector of means corresponding to each solution, and $\mu_{l}^{i}$ can be expressed as Eq. (4).


(2) $$w_{l}=\frac{1}{q\,k\,\sqrt{2\,\pi }}\,e^{-\frac{{\left(i-l\right)}^{2}}{2\,q^{2}\,k^{2}}}$$

(3) $$\sigma_{l}^{i}=\xi \,\sum_{e=1}^{k}\frac{|x_{e}^{i}-x_{l}^{i}}{k-1}$$

(4) $$\mu^{i}=\left\{\mu_{1}^{i},\ldots \,\mu_{l}^{i},\ldots \,\mu_{k}^{i}\right\}=\left\{x_{1}^{i},\ldots \,x_{l}^{i},\ldots \,x_{k}^{i}\right\}$$

where *q* and ξ > 0 are the algorithmic parameters, and the weight *w_l_* is essentially a Gaussian function with *l* as the mean and *qk* as the standard deviation. The sampling procedure is then carried out in practice so that the probability *p_l_* is determined in the first stage using the weight size *w_l_* and Eq. (5). In the second phase, a Gaussian function $g_{l}^{i}\,\left(x\right)$ is picked using the probability *p_l_* from the Gaussian kernel function *G^i^*(*x*), and the selected guiding solution *x_l_* is calculated. The third phase uses the Gaussian function $g_{l}^{i}\,\left(x\right)$ to sample each of the *n* dimensions of the guiding solution *x_l_*.


(5) $$p_{l}=\frac{W_{l}}{\sum_{r=1\,W_{r}}^{k}}$$

**FIGURE 2:**
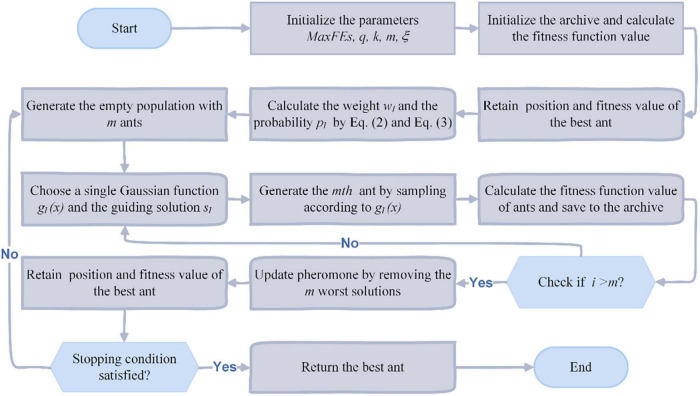
The flowchart of ACOR.

After the completion of sampling, its pheromone updating process is represented by the updating process of the solutions in the archive. In each iteration, the *m* new solutions constructed by the ants are combined with the *k* old solutions in the archive, and the *k* better solutions from these *m+k* solutions are selected and sorted into the archive, while the remaining *m* worse solutions are discarded. Further, according to the algorithm principle of ACOR, Algorithm 1 gives its corresponding pseudo-code, and Figure 2 further gives its corresponding flowchart according to the pseudo-code.

## 3. Proposed MGACO

In MGACO, not only a new movement strategy is introduced, but also the Cauchy-Gaussian fusion strategy is incorporated after the movement strategy. Due to the introduction of the new movement strategy and the Cauchy-Gaussian fusion strategy, MGACO has been accelerated in terms of convergence speed, and the ability of MGACO to jump out of local optimum has been significantly enhanced.

### 3.1. The novel movement strategy

In 2015, Mirjalili et al. (2016) proposed an optimizer based on multiverse theory with a strong ability to jump out of the local optimum. Inspired by this, a new ant movement strategy is proposed by analyzing the movement principles of white holes, black holes, and wormholes in this optimizer.

In MGACO, when the archive is updated, the ants in the archive will continue to search for the optimal food by mimicking the movement principle of the universe individuals. Therefore, the ants will mimic the movement of the universe individuals in order to achieve local changes, and it will excite the internal objects to move toward the current optimal individual, which can be expressed as Eq. (6).


(6) $$x_{l}^{i}=\left\{ \begin{array}{cc} \left\{ \begin{array}{c} x_{l}+T\,D\,R\times \left(\left(u\,b_{l}-l\,b_{l}\right)\times r_{4}+l\,b_{l}\right),\\ r_{3}<0.5\\ x_{l}-T\,D\,R\times \left(\left(u\,b_{l}-l\,b_{l}\right)\times r_{4}+l\,b_{l}\right),\\ r_{3}\geq 0.5 \end{array}\right., & r_{2}<W\,E\,P\\ x_{l}^{i}, & r_{2}\geq W\,E\,P \end{array}\right.$$

where $x_{l}^{i}$ denotes the *ith* dimension of the current ant individual *l*, *lb*_*l*_ and *ub*_*l*_ refer to the movement boundary of $x_{l}^{i}$, and *r*_2_,*r*_3_,*r*_4_ are random numbers in the range of [0, 1]. In MVO, *WEP* denotes the probability of wormhole existence, and *TDR* denotes the step length of the current individual moving toward the current optimal individual. Its update rule is represented by Eqs (7, 8).


(7) $$W\,E\,P=W\,E\,P_{m\,i\,n}+t\times \left(\frac{W\,E\,P_{m\,a\,x}-W\,E\,P_{m\,i\,n}}{M\,a\,x\,F\,E\,s}\right)$$

(8) $$T\,D\,R=1-\frac{F\,E\,s^{1/p}}{M\,a\,x\,F\,E\,s^{1/p}}$$

where *FEs* is the number of current iterations, *MaxFEs* is the maximum number of iterations, and *p* denotes the exploitation level.

### 3.2. Cauchy-Gaussian fusion strategy

The Cauchy-Gaussian fusion strategy is a motion strategy that incorporates the Cauchy distribution and Gaussian distribution, and Kumar et al. (2017) applied this strategy to SCA, which resulted in a significant enhancement of its ability to jump out of the local optimum. Inspired by this, to further enhance the ability of MGACO to avoid falling into local optimum, after the individual ants simulate the locomotor behavior of the multiverse, the individuals in the archive will continue to move toward the optimal food according to the Cauchy-Gaussian fusion strategy. The Cauchy distribution and Gaussian distribution function are described as Eqs (9, 10).


(9) $$f_{c}\,\left(v\right)=\frac{1}{\pi }\,\frac{\gamma }{\gamma^{2}+{\left(v-v_{0}\right)}^{2}}$$

(10) $$f_{N}\,\left(v\right)=\frac{1}{\sqrt{2\,\pi \,\sigma }}\,e^{\left[-{\left(v-u\right)}^{2}/2\,\sigma^{2}\right]}$$

where γ is proportional parameter, *v_0_* is the peak position of the Cauchy distribution *C*(*v*_0_,γ^2^), σ^2^ and *u* are the variance and the mean value of the normal distribution *N*(*u*,σ^2^), respectively. Therefore, *C*(0,1) is also a Cauchy random number, and *N*(0,1) is also a normal random number.

On the basis of the Cauchy distribution and Gaussian distribution, the Cauchy-Gaussian fusion strategy can be described as shown in Eq. (11).


(11) $$x_{l}^{i}=x_{l}^{i}\times \left(1+\delta \times \left(\eta \times N\,\left(0,1\right)\right)+\left(1-\eta \right)\times C\,\left(0,1\right)\right)$$

where δ is inertia constant, and η=*FEs*/*MaxFEs*.

### 3.3. The proposed MGACO

**FIGURE 3:**
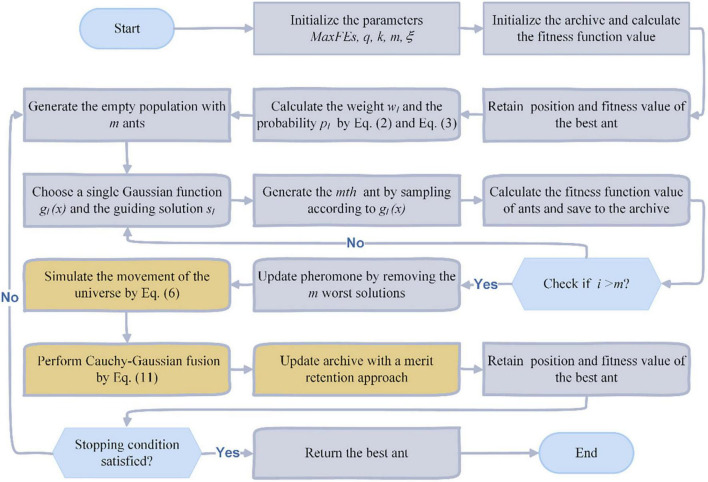
The flowchart of MGACO.

In order to enable ACOR to have some speedup in terms of convergence speed when dealing with real-world problems, as well as to be enhanced in terms of the ability to jump out of local optima, we propose an enhanced version of ACOR called MGACO. In MGACO, when the archive is updated, first let the ants in the archive will simulate the movement principle of the individuals in the MVO to continue searching for the optimal food, and the ants will move toward the current optimal individual in order to achieve local changes. After that, let the individuals in the archive move toward the optimal food according to the Cauchy-Gaussian fusion strategy. After completing two successive movements of ants, the ants in the archive are finally updated again in a constructive manner. Therefore, the introduction of the new moving strategy and the Cauchy-Gaussian fusion strategy makes MGACO speed up in the convergence speed and enhances the ability of MGACO to jump out of the local optimum significantly. According to the basic principle of MGACO, Figure 3 further gives the corresponding flowchart of MGACO according to the pseudo-code.

## 4. Proposed MIS model

### 4.1. Non-local means for 2D histogram

The algorithm’s starting point is that the picture created by categorizing and weighting the regions with the same attributes in the same image should be superior in terms of noise reduction. That is, it employs all pixels in the picture, or more specifically, all pixels within a search window, which are weighted and averaged based on some similarity. Unlike bilinear and median filtering, which utilize local information in the picture to filter, denoising uses the entire image. To better filter out Gaussian noise in the picture, it looks for comparable regions in the image in terms of image blocks (neighborhoods) and then averages these regions. It is feasible to determine the non-local mean values for image *I* using Eqs (12–15), assuming that (*p*) and *I*(*q*) are the corresponding gray-scale values of pixels *p* and *q*.


(12) $$O\,\left(p\right)=\frac{\sum_{q\in I}I\,\left(q\right)\,\omega \,\left(p,q\right)}{\sum_{q\in I}\omega \,\left(p,q\right)}\;$$

(13) $$\omega \,\left(p,q\right)=e^{-\frac{{\left|\mu \,\left(p\right)-\mu \,\left(q\right)\right|}^{2}}{\sigma^{2}}}\;$$

(14) $$\mu \,\left(p\right)=\frac{1}{m\times m}\,\sum_{i\in L\,\left(p\right)}I\,\left(i\right)$$

(15) $$\mu \,\left(q\right)=\frac{1}{m\times m}\,\sum_{i\in L\,\left(q\right)}I\,\left(i\right)$$

where *O*(*p*) is a corresponding filter value, ω(*p*,*q*) is the corresponding weight, and σ is the corresponding standard deviation, and *L*(*p*) and *L*(*q*) are *m*×*m* blocks orientated at *p* and *q*, respectively.

**FIGURE 4:**
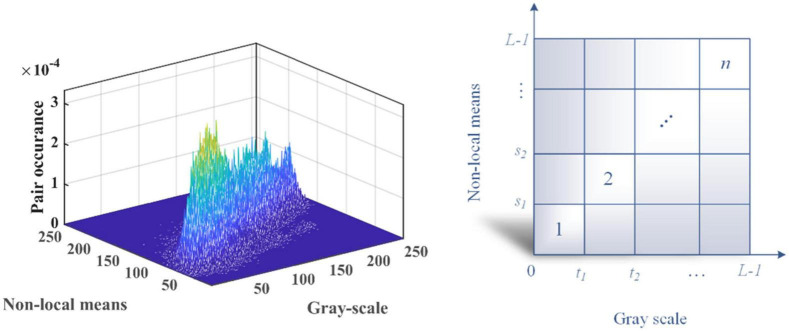
The 2D histogram and the 2D plan view.

A 2D histogram for an image may be produced using grayscale and non-local mean pictures. If we suppose that a gray image *I*(*x*,*y*) has levels [0,*L*−1] and an image size of *M*×*N*, then the picture *g*(*x*,*y*) produced *via* non-local means filtering likewise has these properties. Consequently, the level and gray values of *I*(*x*,*y*) and *g*(*x*,*y*) may be used to create the point (*i*,*j*). The gray value of the pixel in *I*(*x*,*y*) is denoted by *I* while the equivalent pixel in g is denoted by *j*. In light of this, it is also conceivable to have the quantity of pixels *h*(*i*,*j*) that occur at this location (*s*,*t*). This method of developing a 2D histogram is standardized by Eq. (16). A final 2D histogram that we may create is displayed in Figure 4 along with the accompanying plane view.


(16) $$P_{i\,j}=\frac{h\,\left(i,j\right)}{M\times N}$$

### 4.2. 2D Kapur’s entropy

The Kapur entropy thresholding image segmentation method utilizes the important concept of Shannon entropy in information theory. In information theory, entropy is a physical quantity used to measure the degree of uniformity of a given distribution; a higher entropy value indicates a more uniform distribution (Hilali-Jaghdam et al., 2020). Applied in the field of image segmentation, the entropy value of the image grayscale histogram is measured to find a pixel point such that the maximum amount of information is distributed between the target and background regions in the image, and that pixel point is the threshold image segmentation point. It is possible to produce the 2D histogram and 2D plane view shown in Figure 4 using the above-discussed non-local mean 2D histogram idea, where {*t*_1_,*t*_2_,*L*−1} signify the levels of the grayscale image and {*s*_1_,*s*_2_,*L*−1} the levels of the non-local mean image, respectively.

The majority of the visual data in the 2D histogram is concentrated along its main axis, so the 2D Kapur’s entropy is only computed for the n subsections on the center diagonal to make calculation simpler and more precise. As a result of the foregoing explanation, the 2D Kapur’s entropy is shown in the figure as Eq. (17). As a result, when the 2D Karpur’s entropy is taken into account as the objective function of MGACO, the threshold set {*t*_1_,*t*_2_,,*t*_*n*−1_} that maximizes φ(*s*,*t*) is the optimal threshold set.


(17) $$\varphi \,\left(s,t\right)=-\sum_{i=0}^{s_{1}}\sum_{j=0}^{t_{1}}\frac{P_{i\,j}}{P_{1}}\,\ln\frac{P_{i\,j}}{P_{1}}-\sum_{i=t_{1}+1}^{s_{2}}\sum_{j=t_{1}+1}^{t_{2}}$$

$$\frac{P_{i\,j}}{P_{2}}\,\ln\frac{P_{i\,j}}{P_{2}}-\sum_{i=s_{L-2}+1}^{s_{L-1}}\sum_{j=t_{L-2}+1}^{t_{L-1}}\frac{P_{i\,j}}{P_{L-1}}\,\ln\frac{P_{i\,j}}{P_{L-1}}$$

where


$$P_{1}=\sum_{i=0}^{s_{1}}\sum_{j=0}^{t_{1}}P_{i\,j},P_{2}=\sum_{i=t_{1}+1}^{s_{2}}\sum_{j=t_{1}+1}^{t_{2}}P_{i\,j},P_{L-1}$$

$$=\sum_{i=s_{L-2}+1}^{s_{L-1}}\sum_{j=t_{L-2}+1}^{t_{L-1}}P_{i\,j}.$$

### 4.3. The proposed MGACO-MIS method

The main objective of threshold-based segmentation, an effective image segmentation approach, is to find an appropriate threshold set to distinguish the target from the background in a picture. The technique of identifying a threshold set in an image and using that set to split the image into several pieces is also defined as MIS. In order to achieve improved one-threshold image segmentation, Pun (1981) presented a maximum entropy-based thresholding approach that analyzes the histogram of a picture as a probability distribution and calculates the maximum entropy to establish an optimum threshold value. Later, Kapur et al. (1985) presented Kapur’s entropy, an easy-to-compute modified maximum entropy-based threshold segmentation technique that yields better segmentation results. The temporal complexity is *O*((*L*−*M* + 1)^*M*−1^) and it increases exponentially when the exhaustive approach is used to segment a picture with many layers of maximum entropy.

*L* is the grayscale range of the image, and *M* denotes the number of segmentation levels. Although 1D histogram-based segmentation is more common, a serious misclassification issue exposes the segmentation results to noise interference when the target only takes up a small portion of a picture. The conventional 2D histogram segmentation technique, based on the local mean, does not take into account certain precise information in an image, such as some points, lines, planes, etc. (Buades et al., 2005).

**FIGURE 5:**
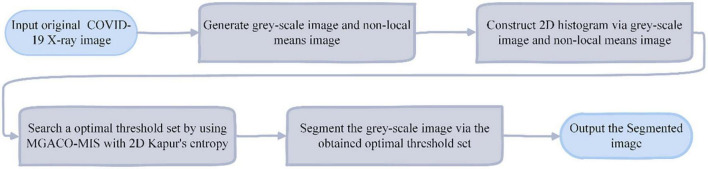
The flowchart of the MGACO-MIS method.

The suggested MIS approach is based on 2D histograms with non-local means and employs 2D Kapur’s entropy as the fitness function of MGACO to lessen the aforementioned constraints. Figure 5 gives a thorough description of it and is also used for the multi-level segmentation of COVID-19 X-ray pictures.

## 5. Experiments and results

We employ 30 benchmark functions from IEEE CEC2014 as our starting point to qualitatively assess MGACO and ACOR in great depth as well as compare MGACO with 10 conventional basic algorithms and 10 variations based on basic algorithms in order to illustrate these essential benefits of MGACO firmly. The acquired experimental findings show conclusively that MGACO has improved leap out of local optimum capability in addition to a certain speedup in convergence. In image processing tasks, it is vital to utilize a valid dataset covering various features and properties to assess the method sincerely (Jin et al., 2022). We also performed comparison studies between MGACO-MIS and eight other comparable segmentation algorithms at various threshold levels using genuine pathological photos of COVID-19 in order to show that MGACO-MIS can provide high-quality segmentation results while doing image segmentation. The acquired experimental findings adequately show that the suggested MGACO-MIS may provide high quality segmentation results while conducting image segmentation.

### 5.1. Experiment setup

To prove the core advantages of MGACO, we first conducted a strategy analysis experiment, followed by a scalability test of MGACO, and finally compared MGACO with some common basic algorithms and some variant algorithms in the experiment. In the strategy analysis experiments, various variants of ACOR were constructed using the new movement strategy and Cauchy-Gaussian fusion strategy, namely MACO, GACO, and MGACO, and comparative strategy combinations were used conducted. Then a directional analysis of MGACO was also performed, including a multi-angle, balanced, and diversity analysis. In the scalability experiments of MGACO, MGACO, and ACOR are compared in different dimensions, where the dimensions are set to 10, 20, 50, and 100, respectively. In the comparative experiments between MGACO and its peers, the comparison experiments were mainly conducted first between MGACO and 10 traditional basic algorithms involving MVO, ACOR, BA, DE, FA, GWO, MFO, PSO, SCA, WOA. Further, we compared MGACO with 10 very high-performance variants, including OBLGWO, OBSCA, RCACO, RCBA, ACWOA, BMWOA, CDLOBA, EWOA, HGWO, m_SCA. The benchmark functions used in the benchmark function experiments, which include unimodal, fundamental multimodal, hybrid, and composition functions, are shown in Table 1. The population size for each method in the comparison is 30, and the maximum number of evaluations is 300,000. All algorithms are performed under these identical settings. Doing this guarantees the validity and trustworthiness of the experimental results. To decrease the influence of chance events, each algorithm is also independently tested 30 times. Comprehensive statistics and analysis were performed using the mean, variance, Wilcoxon signed-rank test, and Friedman test for all experimental results on the benchmark functions. The results unambiguously demonstrate that MGACO not only shows some acceleration in convergence speed but also significantly increases its ability to diverge from the local optimum.

**TABLE 1:** The brief description of IEEE CEC2014.

Class	ID	Description	Range	F*_i_ = F*_i_(x*)
Unimodal functions	1	Rotated high conditioned Elliptic function	[−100, 100]	100
	2	Rotated bent cigar function	[−100, 100]	200
	3	Rotated discus function	[−100, 100]	300
Simple multimodal functions	4	Shifted and rotated Rosenbrock’s function	[−100, 100]	400
	5	Shifted and rotated Ackley’s function	[−100, 100]	500
	6	Shifted and rotated Weierstrass function	[−100, 100]	600
	7	Shifted and rotated Griewank’s function	[−100, 100]	700
	8	Shifted Rastrigin’s function	[−100, 100]	800
	9	Shifted and rotated Rastrigin’s function	[−100, 100]	900
	10	Shifted Schwefel’s function	[−100, 100]	1000
	11	Shifted and rotated Schwefel’s function	[−100, 100]	1100
	12	Shifted and rotated Katsuura function	[−100, 100]	1200
	13	Shifted and rotated HappyCat function	[−100, 100]	1300
	14	Shifted and rotated HGBat function	[−100, 100]	1400
	15	Shifted and rotated expanded Griewank’s plus Rosenbrock’s function	[−100, 100]	1500
	16	Shifted and rotated expanded Schaffer’s F6 function	[−100, 100]	1600
Hybrid functions	17	Hybrid function 1 (*N* = 3)	[−100, 100]	1700
	18	Hybrid function 2 (*N* = 3)	[−100, 100]	1800
	19	Hybrid function 3 (*N* = 4)	[−100, 100]	1900
	20	Hybrid function 4 (*N* = 4)	[−100, 100]	2000
	21	Hybrid function 5 (*N* = 5)	[−100, 100]	2100
	22	Hybrid function 6 (*N* = 5)	[−100, 100]	2200
Composition functions	23	Composition function 1 (*N* = 5)	[−100, 100]	2300
	24	Composition function 2 (*N* = 3)	[−100, 100]	2400
	25	Composition function 3 (*N* = 3)	[−100, 100]	2500
	26	Composition function 4 (*N* = 5)	[−100, 100]	2600
	27	Composition function 5 (*N* = 5)	[−100, 100]	2700
	28	Composition function 6 (*N* = 5)	[−100, 100]	2800
	29	Composition function 7 (*N* = 3)	[−100, 100]	2900
	30	Composition function 8 (*N* = 3)	[−100, 100]	3000

**FIGURE 6:**
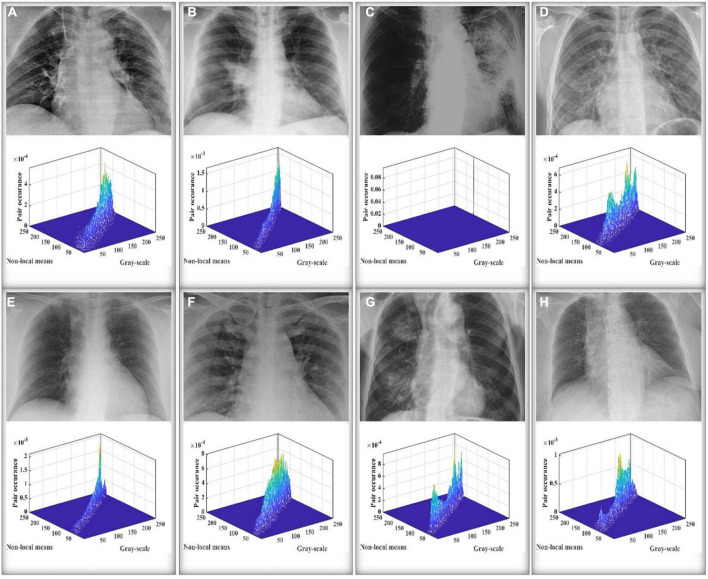
The COVID-19 X-ray images used in the segmentation experiment.

Then, to demonstrate that MGACO-MIS may provide improved segmentation results, we used X-ray images of 8 COVID-19 patients from a public database produced by Cohen et al. (2020). Segmentation experiments were first carried out to represent low threshold levels using MGACO-MIS and 8 other equivalent approaches at levels 4, 5, and 6. Second, to illustrate high threshold levels, we also ran segmentation trials at levels 15, 20, and 25. The letters A, B, C, D, E, F, G, and H in Figure 6 represent the X-ray images of the COVID-19 patients who participated in the segmentation investigations. These comparisons were conducted using the following methodologies: ACOR-MIS, MVO-MIS, HHO-MIS, SCA-MIS, BLPSO-MIS, IGWO-MIS, IWOA-MIS, and CLPSO-MIS. To guarantee fairness (Chen P. et al., 2022; Liu et al., 2022a,c; Yang et al., 2022), and the reliability of the findings, 100 iterations were used in each segmentation experiment. The population size for each segmentation technique included in the comparison was set to 20, the size of the selected segmentation pictures was set to 512 × 400, and each experiment was done 30 times separately to avoid randomization. We first evaluated the segmentation results using the three commonly used evaluation indices—PSNR, SSIM, and FSIM. Second, a detailed analysis of the assessment results was performed using the mean, variance, Wilcoxon signed-rank test, Friedman test, and other statistical techniques. The assessment and analysis results conclusively demonstrate that the suggested MGACO-MIS is suitable for producing outstanding segmentation results in image segmentation, which is the last but not least point.

### 5.2. Benchmark function validation

In this subsection, in order to demonstrate the core advantages of MGACO, firstly, a strategy analysis experiment is conducted; secondly, the scalability test of MGACO is conducted; and finally, MGACO is compared with some common basic algorithms and some variants of algorithms. All the results fully support that MGACO has a certain faster convergence rate and its ability to escape from the local optimum is significantly enhanced.

#### 5.2.1. The impact of two novel enhanced strategies

The new movement strategy and the Cauchy-Gaussian fusion strategy construct the four different variant algorithms of ACOR shown in Table 2, where “NM” denotes the new movement strategy, “GCF” denotes the Cauchy-Gaussian fusion strategy, “1” denotes that the strategy is included in the variant, and “0” denotes that the strategy is not included in the variant. This section first conducted comparative experiments using the four constructed variant algorithms, and its results fully demonstrate that the best performance is obtained only when both strategies are used for the MGACO variants formed in ACOR. The new movement strategy and the Cauchy-Gaussian fusion strategy construct the four different variant algorithms of ACOR shown in Table 2, where “NM” denotes the new movement strategy, “GCF” denotes the Gaussian fusion strategy, “1” denotes that the strategy is included in the variant, and “0” denotes that the strategy is not included in the variant. This section first conducted comparative experiments using the four constructed variant algorithms, and its results fully demonstrate that the best performance is obtained only when both strategies are used for the MGACO variants formed in ACOR. Then, this section continues to analyze MGACO and AOCR not only in multi-perspective but also in balance and diversity.

**TABLE 2:** The constructed four variants with two novel enhanced strategies.

Method	NM	GCF
ACOR	0	0
GACO	0	1
MACO	1	0
MGACO	1	1

Supplementary Table 1 contains the findings from testing the four variations on IEEE CEC2014, where “AVG” and “STD” stand for the mean and variance of the variants obtained after 30 separate runs, respectively. The terms “Mean” and “Rank” refer to ranking results based on the overall mean. The terms “+” and “-” refer to the number of functions where MGACO performs better than its peers, respectively, while “ = ” signifies the number of functions where MGACO performs equally with its peers. As shown in Supplementary Table 1, MGACO achieves the least mean by a significant margin, indicating that the ACOR improved by the two improvement procedures is the best. MGACO is ranked first with a “Mean” value of 1.57, which is substantially higher than GACO, which is ranked second with a “Mean” value of 2.20.

**FIGURE 7:**
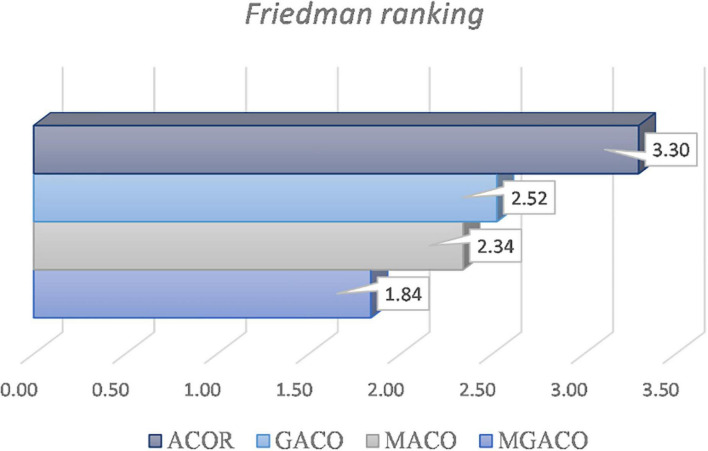
Some convergence curves on benchmark functions.

The convergence curves for the four versions are shown in Supplementary Figure 1, and the convergence processes for F9, F11, F16, and F18 demonstrate that MGACO has the strongest capacity to leap out of the local optimum as a slightly quicker convergence pace than the other variants. Figure 7 displays the findings from the Friedman test analysis of the four variants, where MGACO ranks first with a score of 1.84, outperforming the other three variants. This further demonstrates that the MGACO created using the new movement strategy and the Cauchy-Gaussian fusion strategy has the best outcomes.

Supplementary Figure 2 displays the findings from several viewpoints of research on MGACO. Supplementary Figure 2A shows the distribution of the benchmark functions from a 3D viewpoint. The ideal site is shown by a red dot in Supplementary Figure 2B, while black dots indicate investigated positions. Supplementary Figure 2B shows the 2D distribution of the searched locations. Supplementary Figure 2C displays the variation of the first dimension of the individual position over time. Supplementary Figure 2D illustrates how the average fitness of all subjects changed during the course of iteration. Supplementary Figure 2E shows the convergence curves for MGACO and ACOR. It can be demonstrated, through the distributions in two and three dimensions, that MGACO eventually locates the ideal solution after performing all potential iterations for benchmark functions of varying complexity. The initial oscillation of the individuals’ positions is large, but it gradually decreases and converges as they iterate through the search space, allowing them to avoid being stuck in a local optimum and improve their overall performance. According to the downward oscillation of the average fitness curve of all individuals, all persons may eventually sustain convergence to the optimum solution throughout the search process until they arrive at the perfect solution. The convergence curves from MGACO are more accurate overall than those from AOCR, and those from F9, F24, and F25 are also more accurate overall than those from AOCR. The convergence curves for the two approaches indicate that MGACO has superior final convergence accuracy than AOCR, and the convergence curves for F9, F24, and F25 also demonstrate that MGACO converges more rapidly than AOCR. The convergence curves on F6, F8, as well as MGACO’s higher potential to diverge from the local optimum, provide more evidence of this.

**FIGURE 9:**
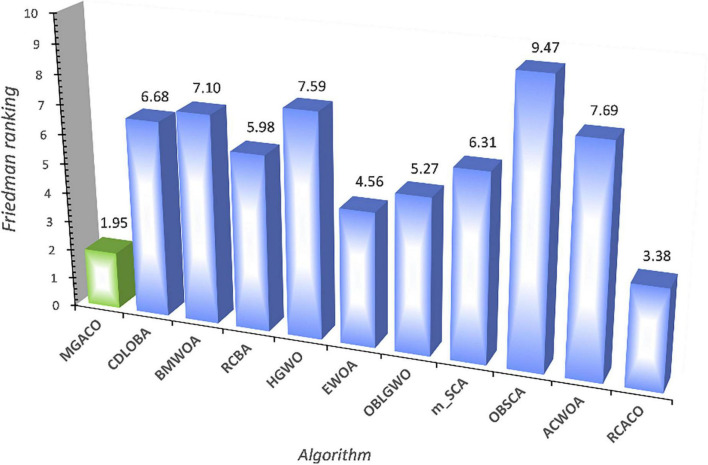
Friedman test results of MGACO and its 10 advanced peers.

Supplementary Figure 3 displays the analytical results for variety and balance on the pertinent benchmark functions. Supplementary Figure 3A displays the balance findings for MGACO, Supplementary Figure 3B displays the balance results for ACOR, and Supplementary Figure 3C displays the balance results for both MGACO and ACOR. It is evident by comparing the results of the exploration and exploitation balance between MGACO and ACOR that the unique movement strategy and Cauchy-Gaussian fusion technique aid MGACO in achieving a superior balance, enabling it to reach convergence more quickly and precisely than AOCR. Only on F8 and F9 do the diversity curves of MGACO converge slower than AOCR in the early stage and faster than ACOR in the late stage. This indicates that MGACO can more fully traverse the entire search space in the early stage and that the search individuals can approach the optimal position too quickly in the late stage.

#### 5.2.2. The scalability test for MGACO

To make a more comprehensive comparison of the performance of MGACO and ACOR, this subsection sets the problem dimensions to 10, 30, 50, and 100 based on IEEE CEC 2014, followed by a comparative analysis of MGACO and ACOR. The corresponding experimental results are given in Supplementary Table 2, where the mean values of MGACO on all problem functions are smaller than ACOR when the problem dimensions are 30 and 50, and the mean values of MGACO on all 28 problem functions are smaller than ACOR when the problem dimensions are 10 and 100, which indicates that MGACO completely outperforms ACOR when dealing with different problems. The results of the Wilcoxon signed-rank test are also presented in Supplementary Table 2, where MGACO completely outperforms ACOR on 24 functions at dimension 10, 26 functions at dimension 30, and 25 functions at dimension 50, and on 26 functions at dimension 100. As can be seen, the results of the Wilcoxon signed-rank test also further illustrate that the advantage of MGACO over AOCR is huge regardless of the problem’s dimensionality. Moreover, Supplementary Figures 3–7 give the convergence curves on functions F1, F3, F12, F20, F24, F27, F28, F29, F30 when the problem dimensions are 10, 30, 50, 100, respectively. In the given convergence curves, it is well demonstrated that MGACO not only has a certain acceleration in the convergence speed, but also its ability to jump out of the local optimum has been significantly enhanced.

The aforementioned experimental findings comparing MGACO and AOCR on 30 function problems demonstrate that MGACO is more stable and can produce better outcomes when the difficulty of the issues varies. Additionally, MGACO has a superior capacity to resist entering local optima throughout the problem-solving process, which aids in coming up with a better solution. Additionally, the convergence accuracy and speed of MGACO have been enhanced. In conclusion, the suggested MGACO outperforms AOCR in terms of benefits and problem-solving strength.

#### 5.2.3. Comparison with some conventional methods

**FIGURE 8:**
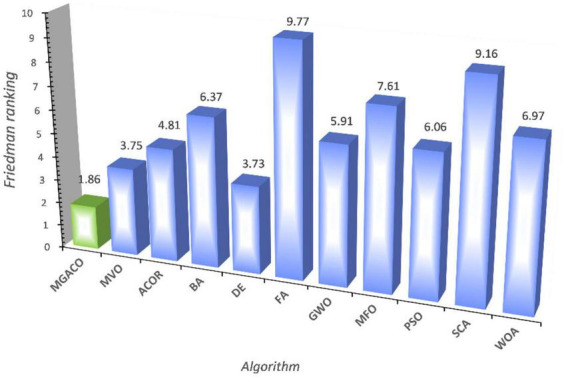
Friedman test results of MGACO and its 10 basic peers.

The major goal of this subsection is to contrast MGACO with a few relatively traditional basic algorithms in order to highlight the technology’s key benefits better. In this experiment, the fundamental algorithms up for comparison are MVO, ACOR, BA, DE, FA, GWO, MFO, PSO, SCA, and WOA, and the algorithms themselves choose the parameter values. The comparison between MGACO and the ten fundamental algorithms is shown in Supplementary Table 3, along with the mean, variance, and Wilcoxon signed-rank test analysis findings. According to the optimal values of the functions that were obtained, MGACO obtained the optimal value, or minimum mean value, on 20 function problems, whereas DE, MVO, and WOA only obtained the optimal value on 7 function problems, 2 function problems, and 1 function problem, respectively. This shows that MGACO still has a greater advantage over the fundamental algorithms. Additionally, according to the Wilcoxon signed-rank test findings, MGACO outperformed the DE, rated No. 2, on the 21 function problems to achieve the top spot with a mean value of 1.43. Figure 8 displays the results of the Friedman test for MGACO and the 10 fundamental techniques. MGACO ranks first with an attained value of 1.86, while DE ranks second with an obtained value of 3.73, further demonstrating that MGACO still has an edge over the fundamental algorithms. Supplementary Figure 8 compares the convergence curves of MGACO and 10 fundamental methods for a set of benchmark functions. The convergence curves F6, F23, F25, and F28 show how MGACO has improved in terms of convergence speed, while F1, F11, F16, and F17 demonstrate how MGACO outperforms other algorithms in terms of avoiding local optimums. Therefore, when MGACO is compared to other fundamental algorithms, its main benefits are also clearly shown.

#### 5.2.4. Comparison with some excellent variants

**FIGURE 10:**
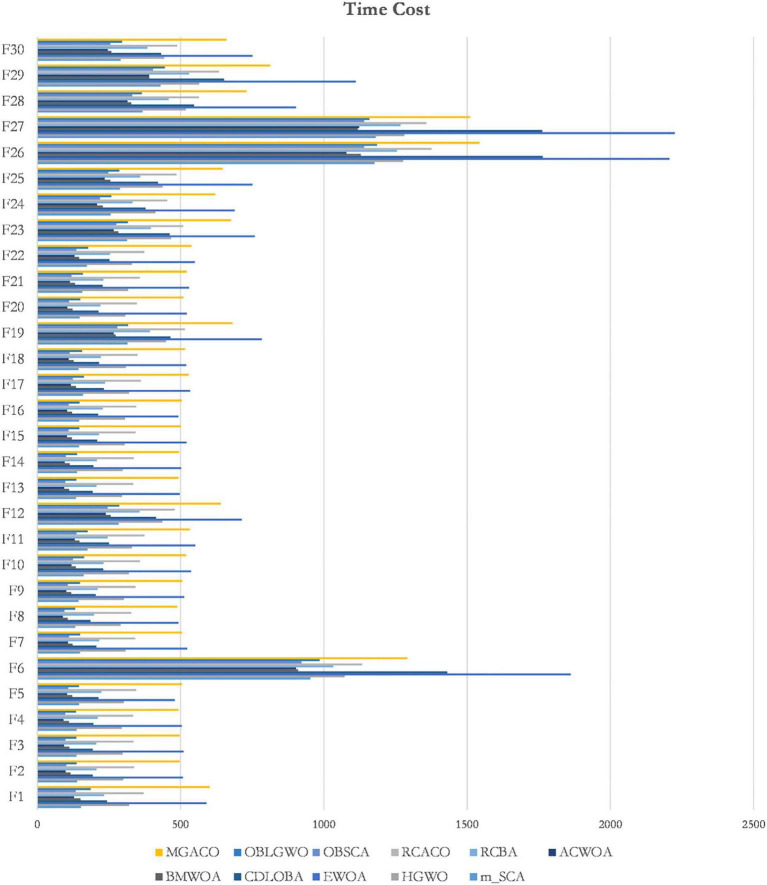
The total CPU time consumption of all methods for 30 independent runs.

This section compares MGACO with various newly suggested enhanced variations to better highlight the fundamental benefits of the algorithm. The comparison included the algorithms OBLGWO, OBSCA, RCACO, RCBA, ACWOA, BMWOA, CDLOBA, EWOA, HGWO, and m_SCA. Supplementary Table 4 displays the experimental results for each algorithm after 30 separate runs, including the mean, variance, and Wilcoxon signed-rank test analysis findings. In terms of finding the best solutions to function problems, MGACO finds the best solutions to 16 of them, RCACO, CDLOBA, RCBA, EWOA, and HGWO each find the best solutions to just 4 of them, MGACO finds the best solutions to 3 function problems, and MGACO finds the best solutions to just 1 function problem. As a result, MGACO outperforms all other advanced peers and has the best performance among the optimum values found for function issues. Additionally, we can observe that MGACO only outperforms RCACO, which is rated. No. 2, on two function problems, further proving that it outperforms other approaches. The Friedman test results are shown in Figure 9, where MGACO comes out on top with a score of 1.95 and RCACO comes in second with a score of 3.38. This further proves that MGACO outperforms other techniques. MGACO has the best convergence accuracy on all benchmark functions, the fastest convergence rate on F4, F20, and F29 than other techniques, and the strongest capacity to jump out of local optimum on F9, F11, and F16 in the convergence curves shown in Figure 9. As a result of the above correlation research, its primary benefits are well highlighted when comparing MGACO to other advanced variations. However, the complexity of the algorithm inevitably increases because of the clever integration of multiple mechanisms into ACO, which can also be illustrated in the total CPU time consumption for 30 independent runs given in Figure 10, where although the time consumption of MGACO is higher than that of some competing algorithms, it is still at the average of its peers. Therefore, we consider such a complexity level to be acceptable.

### 5.3. Experiment on COVID-19 X-ray image segmentation

In this part, we performed our research on the X-ray images of 8 COVID-19 patients to demonstrate the superior segmentation performance of MGACO-MIS. First, segmentation tests were performed at levels 4, 5, and 6 to reflect low threshold levels utilizing MGACO-MIS and another 8 equivalent methodologies. Second, segmentation studies were also performed using levels 15, 20, and 25 to represent high threshold levels.

#### 5.3.1. Performance measures indicators

In this study, three widely used evaluation measures are employed to more clearly show how well the algorithm performs and how well the picture segmentation is done. The three indicators utilized are PSNR, FSIM, and SSIM in the Table 3, with the following definitions for each.

**TABLE 3:** Indicators of the multi-level image segmentation techniques’ performance.

Indicators	Formulation	Remark
Peak signal to noise ratio (PSNR) (Huynh-Thu and Ghanbari, 2008)	$PSNR\;=\;20\cdot log_{10}\left(\frac{255}{RMSE}\right)$	Compare the divided picture to the original image and evaluate the differences.
Structural similarity index (SSIM) (Zhou et al., 2004)	$SSIM\;=\;\frac{\left(2{\text{μ}}_{I}{\text{μ}}_{Seg}+c_{1}\right)\left(2\sigma_{I,Seg}+c_{2}\right)}{\left({\text{μ}}_{I}{}^{2}+{\text{μ}}_{Seg}{}^{2}+c_{1}\right)\left(\sigma_{I}{}^{2}+\sigma_{Seg}{}^{2}+c_{2}\right)}$	Determines if a segmented picture and an uncompressed or distortion-free image are similar.
Feature similarity index (FSIM) (Zhang et al., 2011)	$FSIM\;=\;\frac{\sum_{I\in \Omega }S_{L}\left(X\right)PC_{m}\left(X\right)}{\sum_{I\in \Omega }PC_{m}\left(X\right)}$	Establishes the quality score that measures the importance of a local structure.

Furthermore, a higher value for any one of the three indices mentioned above indicates a better degree of segmented image quality. The SSIM and FSIM index values range is [0, 1]. Further in-depth analysis was performed on the obtained assessment results using the mean, variance, Wilcoxon signed-rank test, and Friedman test. Based on the study and comparison of those experimental outcomes, MGACO-superior MIS’s segmentation effect and its flexibility to different threshold levels are well shown when it is put up against other similar MIS approaches.

#### 5.3.2. Experimental result analyses

**FIGURE 11:**
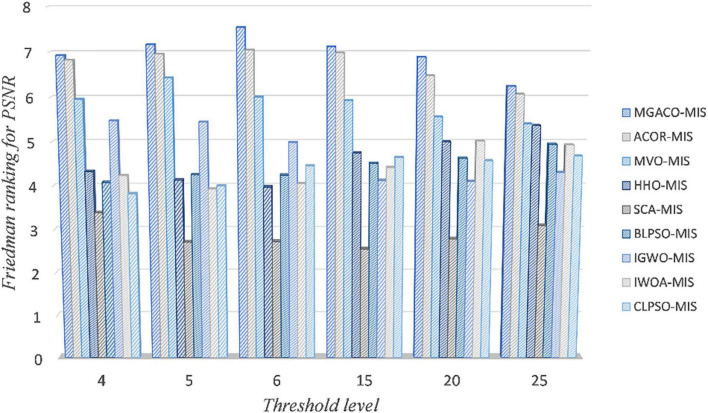
Friedman test results of MGACO-MIS and its peers for PSNR evaluation.

**FIGURE 12:**
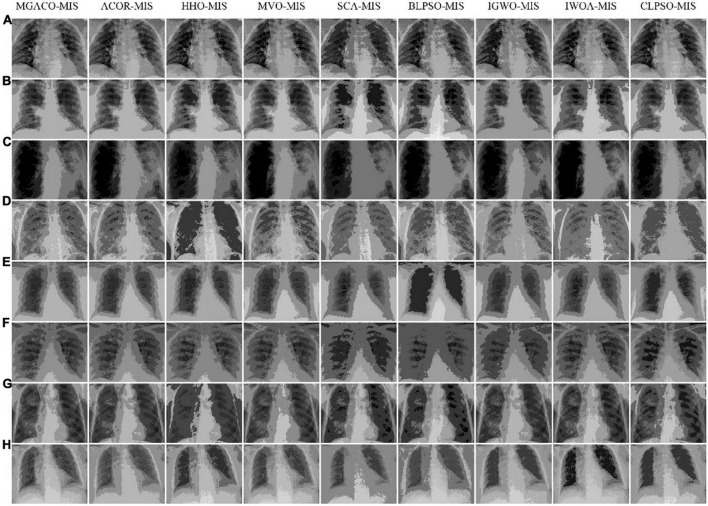
Segmentation results by using 2D Kapur’s entropy for all methods at threshold level 6.

In order to demonstrate that MGACO-MIS may yield better segmentation results on COVID-19 pathology images, this section compares MGACO-MIS with eight other peers at different threshold levels. These peers include the techniques ACOR-MIS, MVO-MIS, HHO-MIS, SCA-MIS, BLPSO-MIS, IGWO-MIS, IWOA-MIS, and CLPSO-MIS. The findings of the assessment using PSNR, FSIM, and SSIM are shown in Supplementary Tables 5–7, and they contain the mean and variance of PSNR, FSIM, and SSIM obtained using all segmentation techniques at threshold levels 4, 5, 6, 15, 20, and 25. It is clear that MGACO-MIS receives the maximum number of optimum evaluation values among the three evaluation results when looking at the number of optimal evaluation results achieved on PSNR, FSIM, and SSIM. This shows that MGACO-MIS is capable of producing extremely good segmentation results. Following additional examination of the PSNR, FSIM, and SSIM assessment findings using the Wilcoxon signed-rank test, the results are presented in Tables 4–6, where the bolded content indicates the first ranked algorithm. MGACO-MIS came in first at various threshold settings, and its performance on most pictures is comparable to that of other approaches. In order to further analyze the PSNR, FSIM, and SSIM assessment outcomes, the Friedman test results are presented in Figure 11 and Supplementary Figures 10, 11, 2. The MGACO-MIS was also able to achieve maximum values at various threshold levels. When the results of the Friedman test, Wilcoxon signed-rank test, Wilcoxon variance test, and PSNR, FSIM, and SSIM evaluations are combined, they show that MGACO-MIS can produce better segmentation results on pathological pictures of COVID-19. Supplementary Figures 12, 13 also show the 2D Kapur’s entropy convergence curves for all techniques used during level 6 and level 25 segmentation, where MGACO–MIS ultimately achieves the highest value of 2D Kapur’s entropy. It further demonstrates that MGACO-MIS can get superior segmentation results on the diseased pictures of COVID-19 when combined with examining the maximum 2D Kapur’s entropy and its convergence curves. Supplementary Table 8 lists the precise segmentation thresholds established using each approach on all pictures when the threshold levels were low. Figure 12 displays the precise segmentation results at threshold level 6, and Supplementary Figure 14 displays the results of the precise segmentation at threshold level 25. Combining the precise segmentation thresholds with the segmentation outcomes at threshold level 6, it is clear that MGACO-MIS produces better segmentation outcomes than other techniques regarding detail retention, local feature retention, and overall segmentation effect. This also suggests that the segmentation thresholds produced by MGACO-MIS are reasonable and capable of completing the segmentation task. Additionally, the precise segmentation outcomes at the threshold level of 25 further suggest that MGACO-MIS can achieve superior segmentation outcomes.

**TABLE 4:** The PSNR analysis results of MGACO and its peers at different threshold levels.

	4			5		
	+/-/=	Mean	Rank	+/-/=	Mean	Rank
MGACO-MIS	**∼**	**1.88**	**1.00**	**∼**	**1.50**	**1.00**
ACOR-MIS	0/0/8	2.00	2.00	0/0/8	1.88	2.00
MVO-MIS	4/0/4	3.13	3.00	3/0/5	3.00	3.00
HHO-MIS	7/0/1	6.00	5.00	8/0/0	6.88	8.00
SCA-MIS	7/0/1	8.00	9.00	8/0/0	9.00	9.00
BLPSO-MIS	7/0/1	6.38	7.00	8/0/0	5.88	5.00
IGWO-MIS	5/0/3	4.13	4.00	6/0/2	4.13	4.00
IWOA-MIS	7/0/1	6.00	5.00	8/0/0	6.63	7.00
CLPSO-MIS	7/0/1	7.50	8.00	8/0/0	6.13	6.00
	**6**			**15**		
	**+/-/=**	**Mean**	**Rank**	**+/-/=**	**Mean**	**Rank**
MGACO-MIS	**∼**	**1.25**	**1.00**	**∼**	**1.38**	**1.00**
ACOR-MIS	1/0/7	1.75	2.00	1/0/7	1.75	2.00
MVO-MIS	6/0/2	3.00	3.00	5/0/3	3.00	3.00
HHO-MIS	8/0/0	6.50	7.00	7/0/1	5.63	5.00
SCA-MIS	8/0/0	9.00	9.00	8/0/0	9.00	9.00
BLPSO-MIS	8/0/0	6.13	5.00	8/0/0	5.88	6.00
IGWO-MIS	7/0/1	4.50	4.00	8/0/0	7.00	8.00
IWOA-MIS	8/0/0	6.50	7.00	8/0/0	6.38	7.00
CLPSO-MIS	8/0/0	6.38	6.00	8/0/0	5.00	4.00
	**20**			**25**		
	**+/-/=**	**Mean**	**Rank**	**+/-/=**	**Mean**	**Rank**
MGACO-MIS	**∼**	**1.38**	**1.00**	**∼**	**1.50**	**1.00**
ACOR-MIS	1/0/7	1.63	2.00	1/0/7	1.63	2.00
MVO-MIS	4/0/4	3.50	3.00	1/0/7	4.13	4.00
HHO-MIS	6/0/2	5.88	6.00	2/0/6	3.63	3.00
SCA-MIS	8/0/0	9.00	9.00	8/0/0	9.00	9.00
BLPSO-MIS	8/0/0	6.00	7.00	3/0/5	5.75	6.00
IGWO-MIS	7/0/1	7.50	8.00	6/0/2	7.13	7.00
IWOA-MIS	5/0/3	4.50	4.00	5/0/3	5.00	5.00
CLPSO-MIS	7/0/1	5.63	5.00	7/0/1	7.25	8.00

The bolded content indicates the first ranked algorithm.

**TABLE 5:** The FSIM analysis results of MGACO and its peers at different threshold levels.

	4			5		
	+/-/=	Mean	Rank	+/-/=	Mean	Rank
MGACO-MIS	**∼**	**1.63**	**1.00**	**∼**	**1.50**	**1.00**
ACOR-MIS	0/0/8	2.00	2.00	0/0/8	2.13	2.00
MVO-MIS	2/0/6	3.25	3.00	3/0/5	2.88	3.00
HHO-MIS	7/0/1	7.25	9.00	7/0/1	6.00	6.00
SCA-MIS	7/0/1	6.38	5.00	8/0/0	8.88	9.00
BLPSO-MIS	8/0/0	7.00	8.00	8/0/0	6.63	7.00
IGWO-MIS	4/0/4	3.75	4.00	4/0/4	4.13	4.00
IWOA-MIS	8/0/0	6.88	6.00	7/0/1	7.13	8.00
CLPSO-MIS	7/0/1	6.88	6.00	8/0/0	5.75	5.00
	**6**			**15**		
	**+/-/=**	**Mean**	**Rank**	**+/-/=**	**Mean**	**Rank**
MGACO-MIS	**∼**	**1.00**	**1.00**	**∼**	**1.50**	**1.00**
ACOR-MIS	1/0/7	2.00	2.00	0/0/8	1.63	2.00
MVO-MIS	5/0/3	3.00	3.00	7/0/1	3.00	3.00
HHO-MIS	8/0/0	6.25	6.00	6/0/2	5.00	4.00
SCA-MIS	8/0/0	9.00	9.00	8/0/0	9.00	9.00
BLPSO-MIS	8/0/0	6.50	8.00	8/0/0	6.38	7.00
IGWO-MIS	8/0/0	4.75	4.00	8/0/0	7.25	8.00
IWOA-MIS	8/0/0	6.38	7.00	8/0/0	5.88	6.00
CLPSO-MIS	8/0/0	6.13	5.00	8/0/0	5.38	5.00
	**20**			**25**		
	**+/-/=**	**Mean**	**Rank**	**+/-/=**	**Mean**	**Rank**
MGACO-MIS	**∼**	**1.38**	**1.00**	**∼**	**1.38**	**1.00**
ACOR-MIS	1/0/7	1.63	2.00	2/0/6	1.88	2.00
MVO-MIS	4/0/4	3.50	3.00	1/0/7	3.75	3.00
HHO-MIS	8/0/0	5.50	5.00	3/0/5	4.25	4.00
SCA-MIS	8/0/0	9.00	9.00	8/0/0	9.00	9.00
BLPSO-MIS	8/0/0	6.25	7.00	7/0/1	5.88	6.00
IGWO-MIS	8/0/0	7.63	8.00	8/0/0	6.75	7.00
IWOA-MIS	6/0/2	4.25	4.00	4/0/4	4.88	5.00
CLPSO-MIS	8/0/0	5.88	6.00	8/0/0	7.25	8.00

The bolded content indicates the first ranked algorithm.

**TABLE 6:** The SSIM analysis results of MGACO and its peers at different threshold levels.

	4			5		
	+/-/=	Mean	Rank	+/-/=	Mean	Rank
MGACO-MIS	**∼**	**2.13**	**1.00**	**∼**	**2.00**	**1.00**
ACOR-MIS	0/0/8	3.00	2.00	0/0/8	2.75	2.00
MVO-MIS	2/0/6	4.13	4.00	3/0/5	3.75	4.00
HHO-MIS	6/0/2	7.38	9.00	5/0/3	7.13	8.00
SCA-MIS	37989.00	4.63	5.00	7/1/0	6.13	6.00
BLPSO-MIS	8/0/0	7.25	8.00	7/0/1	6.75	7.00
IGWO-MIS	37261.00	3.00	2.00	37261.00	3.25	3.00
IWOA-MIS	7/0/1	7.13	7.00	6/0/2	7.38	9.00
CLPSO-MIS	6/0/2	6.38	6.00	7/0/1	5.88	5.00
	**6**			**15**		
	**+/-/=**	**Mean**	**Rank**	**+/-/=**	**Mean**	**Rank**
MGACO-MIS	**∼**	**1.88**	**1.00**	**∼**	**1.25**	**1.00**
ACOR-MIS	1/0/7	2.50	2.00	0/0/8	2.00	2.00
MVO-MIS	4/0/4	4.25	3.00	5/0/3	2.75	3.00
HHO-MIS	7/0/1	7.13	9.00	7/0/1	5.25	4.00
SCA-MIS	7/1/0	7.00	8.00	8/0/0	9.00	9.00
BLPSO-MIS	7/0/1	6.13	7.00	8/0/0	6.50	7.00
IGWO-MIS	5/0/3	4.38	4.00	7/0/1	6.88	8.00
IWOA-MIS	7/0/1	5.88	5.00	8/0/0	6.00	6.00
CLPSO-MIS	5/0/3	5.88	5.00	7/0/1	5.38	5.00
	**20**			**25**		
	**+/-/=**	**Mean**	**Rank**	**+/-/=**	**Mean**	**Rank**
MGACO-MIS	**∼**	**1.50**	**1.00**	**∼**	**1.50**	**1.00**
ACOR-MIS	1/0/7	1.88	2.00	2/0/6	2.38	2.00
MVO-MIS	4/0/4	3.50	3.00	2/0/6	3.50	3.00
HHO-MIS	6/0/2	5.50	5.00	2/0/6	3.75	4.00
SCA-MIS	8/0/0	8.75	9.00	8/0/0	9.00	9.00
BLPSO-MIS	8/0/0	6.13	6.00	6/0/2	5.88	6.00
IGWO-MIS	7/0/1	7.25	8.00	7/0/1	7.00	7.00
IWOA-MIS	5/0/3	4.38	4.00	4/0/4	4.63	5.00
CLPSO-MIS	7/0/1	6.13	6.00	7/0/1	7.38	8.00

The bolded content indicates the first ranked algorithm.

## 6. Discussion

We first conducted a comparison experiment based on the 30 test functions of IEEE CEC2014 between the new moving strategy and the variants created by the Cauchy-Gaussian fusion strategy, where MGACO obtained the minimum mean value the majority of the time on all function problems. MGACO also outperformed the other three variants of the algorithm in the analysis results. Additionally, the effect of the two new mechanisms on ACOR is thoroughly examined from a variety of angles, with an emphasis on diversity and balance. This analysis demonstrates that MGACO further optimizes the balance of exploration and development based on ACOR, enabling MGACO to handle more challenging issues. Then, to analyze the effectiveness of MGACO and ACOR more thoroughly, a comparison of the two programs is made across several aspects. The findings of this comparison conclusively show that MGACO can provide better outcomes and is more stable. MGACO has a superior capacity to resist entering a local optimum throughout the problem-solving process, which aids in developing a better solution. Finally, we evaluate MGACO against certain established, fundamental algorithms and some enhanced variations. The results of the Friedman test analysis, Wilcoxon signed-rank test analysis, and associated convergence curves clearly show that MGACO not only produces high-quality solutions but also has a little improvement in convergence speed and the capacity to depart from the local optimum. In conclusion, this paper’s proposal for the MGACO offers more advantages over the AOCR and a higher capacity for problem-solving. Additionally, we conducted MIS experiments based on actual COVID-19 pathology images, the evaluation results of PSNR, SSIM, and FSIM, and the additional Wilcoxon signed-rank test and Friedman test results can show that MGACO-MIS can achieve better segmentation results on the pathological images of COVID-19. The particular segmentation findings further demonstrate that MGACO-MIS outperforms its competitors in terms of detail preservation, local feature preservation, and overall segmentation outcomes.

As a result, it is undeniably established that MGACO is a very good swarm intelligence optimization algorithm and that MGACO-MIS is an even better segmentation technique when MGACO is used to segment problematic pictures from COVID-19. In future work, the proposed method can also be applied to more cases, such as the optimization of machine learning models iris segmentation and recognition (Chen et al., 2023), fine-grained alignment (Wang et al., 2023), remote pulse extraction (Zhao et al., 2022), Alzheimer’s disease identification (Yan et al., 2022), MRI reconstruction (Lv et al., 2021), renewable energy generation (Sun et al., 2022), power distribution network (Cao et al., 2022), retinal vessel segmentation (Li et al., 2022), privacy protection of personalized information retrieval (Wu Z. et al., 2020; Wu et al., 2021c,d, 2023), and privacy protection of location-based services (Wu et al., 2021b,^2022^).

## 7. Conclusion and future works

An MIS approach (MGACO-MIS) based on an improved version of ACOR (MGACO) is developed in this study. Not only is a new move strategy included in MGACO, but also the Cauchy-Gaussian fusion approach. Due to the addition of the new movement strategy and the Cauchy-Gaussian fusion approach, MGACO’s convergence speed and capacity to jump out of the local optimum have been much improved. To highlight these fundamental benefits of MGACO, the 30 benchmark functions from IEEE CEC2014 are used to compare MGACO and ACOR with 10 conventional basic algorithms and 10 modifications. The outcomes show that MGACO increases convergence speed and considerably increases its capacity to exit the local optimum. In order to show that MGACO can produce high-quality segmentation results when performing image segmentation, a comparison experiment was carried out between MGACO-MIS and eight other comparable segmentation methods using real pathological images of COVID-19 at threshold levels 4, 5, 6, 15, and 25, where 4, 5, and 6 represent low threshold levels and 15, 20, and 25 represent high threshold levels. The final assessment and analysis findings conclusively show that the created MGACO-MIS can produce accurate image segmentation results. However, the proposed method achieves very good results, but the time consumption is still large. To better solve this problem, it will be solved by introducing parallel computing or high-performance computing in the future.

For future work, MGACO will be considered to be applied to more fields, such as bankruptcy prediction (Zhang et al., 2020), engineering design (Chen et al., 2019; Luo et al., 2019; Al-Betar et al., 2020; Tu et al., 2020; Wang et al., 2020), and financial stress prediction (Luo et al., 2018). Further, MGACO-MIS will be considered for the segmentation of more pathological images to achieve greater value and contribute to the advancement of medical diagnosis technology.

## Data availability statement

The original contributions presented in this study are included in the article/Supplementary material, further inquiries can be directed to the corresponding authors.

## Author contributions

XZ, AH, YC, and BM: writing—original draft, writing—review and editing, software, visualization, and investigation. LL and HC: conceptualization, methodology, formal analysis, investigation, writing—review and editing, funding acquisition, and supervision. All authors contributed to the article and approved the submitted version.

## References

[B1] Abd Elaziz M. Oliva D. Xiong S. (2017). An improved opposition-based sine cosine algorithm for global optimization. *Expert Syst. Appl.* 90 484–500. 10.1016/j.eswa.2017.07.043

[B2] Abd Elaziz M. Yousri D. Al-qaness M. A. A. AbdelAty A. M. Radwan A. G. Ewees A. A. (2021). A Grunwald–Letnikov based Manta ray foraging optimizer for global optimization and image segmentation. *Eng. Appl. Artif. Intell.* 98:104105. 10.1016/j.engappai.2020.104105

[B3] Abdel-Basset M. Chang V. Mohamed R. (2020). HSMA_WOA: A hybrid novel Slime mould algorithm with whale optimization algorithm for tackling the image segmentation problem of chest X-ray images. *Appl. Soft Comput.* 95:106642. 10.1016/j.asoc.2020.106642 32843887 PMC7439973

[B4] Al-Betar M. A. Awadallah M. A. Heidari A. A. Chen H. Al-khraisat H. Li C. (2020). Survival exploration strategies for harris hawks optimizer. *Expert Syst. Appl.* 168:114243. 10.1016/j.eswa.2020.114243

[B5] Aranguren I. Valdivia A. Morales-Castañeda B. Oliva D. Abd Elaziz M. Perez-Cisneros M. (2021). Improving the segmentation of magnetic resonance brain images using the LSHADE optimization algorithm. *Biomed. Signal Proc. Control* 64:102259. 10.1016/j.bspc.2020.102259

[B6] Ban Y. Wang Y. Liu S. Yang B. Liu M. Yin L. (2022). 2D/3D multimode medical image alignment based on spatial histograms. *Appl. Sci.* 12:8261. 10.3390/app12168261

[B7] Bernheim A. Mei X. Huang M. Yang Y. Fayad Z. Zhang N. (2020). Chest CT findings in coronavirus disease-19 (COVID-19): Relationship to duration of infection. *Radiology* 295:200463. 10.1148/radiol.2020200463 32077789 PMC7233369

[B8] Buades A. Coll B. Morel J. (2005). “A non-local algorithm for image denoising,” in *2005 IEEE computer society conference on computer vision and pattern recognition (CVPR’05), 20-25 June 2005*, (San Diego, CA: IEEE), 10.1109/CVPR.2005.38

[B9] Cai Z. Gu J. Luo J. Zhang Q. Chen H. Pan Z. (2019). Evolving an optimal kernel extreme learning machine by using an enhanced grey wolf optimization strategy. *Expert Syst. Appl.* 138:112814. 10.1016/j.eswa.2019.07.031

[B10] Cao X. Cao T. Xu Z. Zeng B. Gao F. Guan X. (2022). “Resilience constrained scheduling of mobile emergency resources in electricity-hydrogen distribution network,” in *IEEE transactions on sustainable energy*, (Piscataway, NJ: IEEE), 1–15. 10.1109/TSTE.2022.3217514

[B11] Chakraborty T. Banik S. K. Bhadra A. K. Nandi D. (2021). Dynamically learned PSO based neighborhood influenced fuzzy c-means for pre-treatment and post-treatment organ segmentation from CT images. *Comput. Methods Prog. Biomed.* 202:105971. 10.1016/j.cmpb.2021.105971 33611030

[B12] Chen C.-C. Shen L. P. (2018). Improve the accuracy of recurrent fuzzy system design using an efficient continuous ant colony optimization. *Int. J. Fuzzy Syst.* 20 817–834. 10.1007/s40815-018-0458-7

[B13] Chen H. Li C. Mafarja M. Heidari A. A. Chen Y. Cai Z. (2022). Slime mould algorithm: A comprehensive review of recent variants and applications. *Int. J. Syst. Sci.* 54 1–32. 10.1080/00207721.2022.2153635

[B14] Chen H. Xu Y. Wang M. Zhao X. (2019). A balanced whale optimization algorithm for constrained engineering design problems. *Appl. Math. Model.* 71 45–59. 10.1016/j.apm.2019.02.004

[B15] Chen P. Pei J. Lu W. Li M. deep A. (2022). reinforcement learning based method for real-time path planning and dynamic obstacle avoidance. *Neurocomputing* 497 64–75. 10.1016/j.neucom.2022.05.006

[B16] Chen X. Tianfield H. Mei C. Du W. Liu G. (2017). Biogeography-based learning particle swarm optimization. *Soft Comput.* 21 7519–7541. 10.1007/s00500-016-2307-7

[B17] Chen X. Yao L. Zhou T. Dong J. Zhang Y. (2021). Momentum contrastive learning for few-shot COVID-19 diagnosis from chest CT images. *Pattern Recognit.* 113:107826. 10.1016/j.patcog.2021.107826 33518813 PMC7833525

[B18] Chen Y. Gan H. Chen H. Zeng Y. Xu L. Heidari A. A. (2023). Accurate iris segmentation and recognition using an end-to-end unified framework based on MADNet and DSANet. *Neurocomputing* 517 264–278. 10.1016/j.neucom.2022.10.064

[B19] Chen Z. Wang R.-L. (2017). Ant colony optimization with different crossover schemes for global optimization. *Cluster Comput.* 20 1247–1257. 10.1007/s10586-017-0793-8

[B20] Chen Z. Zhou S. Luo J. (2017). A robust ant colony optimization for continuous functions. *Expert Syst. Appl.* 81 309–320. 10.1016/j.eswa.2017.03.036

[B21] Cohen J. P. Morrison P. Dao L. Roth K. Duong T. Q. Ghassemi M. (2020). COVID-19 image data collection: Prospective predictions are the future, [Online]. *arXiv* [Preprint]. 10.48550/arXiv.2006.11988 35895330

[B22] Deng W. Xu J. Gao X. Z. Zhao H. (2022). An Enhanced MSIQDE algorithm with novel multiple strategies for global optimization problems. *IEEE Trans. Syst. Man Cybern. Syst.* 52 1578–1587. 10.1109/TSMC.2020.3030792

[B23] Derrac J. García S. Molina D. Herrera F. (2011). A practical tutorial on the use of nonparametric statistical tests as a methodology for comparing evolutionary and swarm intelligence algorithms. *Swarm Evol. Comput.* 1 3–18. 10.1016/j.swevo.2011.02.002

[B24] Dinkar S. K. Deep K. Mirjalili S. Thapliyal S. (2021). Opposition-based laplacian equilibrium optimizer with application in image segmentation using multilevel thresholding. *Expert Syst. Appl.* 174:114766. 10.1016/j.eswa.2021.114766

[B25] Dong R. Chen H. Heidari A. A. Turabieh H. Mafarja M. Wang S. (2021). Boosted kernel search: Framework, analysis and case studies on the economic emission dispatch problem. *Knowl. Based Syst.* 233:107529. 10.1016/j.knosys.2021.107529

[B26] Elaziz M. A. Heidari A. A. Fujita H. Moayedi H. (2020). A competitive chain-based Harris Hawks Optimizer for global optimization and multi-level image thresholding problems. *Appl. Soft Comput.* 95:106347. 10.1016/j.asoc.2020.106347

[B27] Elhosseini M. A. Haikal A. Y. Badawy M. Khashan N. (2019). Biped robot stability based on an A–C parametric Whale Optimization Algorithm. *J. Comput. Sci.* 31 17–32. 10.1016/j.jocs.2018.12.005

[B28] Falcon-Cardona J. G. Coello C. A. C. (2017). A new indicator-based many-objective ant colony optimizer for continuous search spaces. *Swarm Intell.* 11 71–100. 10.1007/s11721-017-0133-x

[B29] Gao W. (2015). Identification of constitutive model for rock materials based on immune continuous ant colony algorithm. *Mater. Res. Innov.* 19 S5–S311. 10.1179/1432891714Z.0000000001100

[B30] García S. Fernández A. Luengo J. Herrera F. (2010). A nonparametric tests for multiple comparisons in the design of experiments in computational intelligence and data mining: Experimental analysis of power. *Inform. Sci.* 180 2044–2064. 10.1016/j.ins.2009.12.010

[B31] Heidari A. A. Mirjalili S. Faris H. Aljarah I. Mafarja M. Chen H. (2019c). Harris hawks optimization: Algorithm and applications. *Future Gener. Comput. Syst.* 97 849–872. 10.1016/j.future.2019.02.028

[B32] Heidari A. A. Ali Abbaspour R. Chen H. (2019a). Efficient boosted grey wolf optimizers for global search and kernel extreme learning machine training. *Appl. Soft Comput.* 81:105521. 10.1016/j.asoc.2019.105521

[B33] Heidari A. A. Aljarah I. Faris H. Chen H. Luo J. Mirjalili S. (2019b). An enhanced associative learning-based exploratory whale optimizer for global optimization. *Neural Comput. Appl.* 32 1–27. 10.1007/s00521-019-04015-0

[B34] Hilali-Jaghdam I. Ben Ishak A. Abdel-Khalek S. Jamal A. (2020). Quantum and classical genetic algorithms for multilevel segmentation of medical images: A comparative study. *Comput. Commun.* 162 83–93. 10.1016/j.comcom.2020.08.010

[B35] Hu F. Huang M. Sun J. Zhang X. Liu J. (2021). An analysis model of diagnosis and treatment for COVID-19 pandemic based on medical information fusion. *Inform. Fusion* 73 11–21. 10.1016/j.inffus.2021.02.016 33679271 PMC7919532

[B36] Huang C. Li X. Wen Y. (2021). A OSU image segmentation based on fruitfly optimization algorithm. *Alex. Eng. J.* 60 183–188. 10.1016/j.aej.2020.06.054

[B37] Huang C. Zhou X. Ran X. Liu Y. Deng W. Deng W. (2023). Co-evolutionary competitive swarm optimizer with three-phase for large-scale complex optimization problem. *Inform. Sci.* 619 2–18. 10.1016/j.ins.2022.11.019

[B38] Huang X. (2016). Ant colony optimization algorithm model based on the continuous space. *Int. J. Online Eng.* 12 27–31. 10.3991/ijoe.v12i12.6451

[B39] Huynh-Thu Q. Ghanbari M. (2008). Scope of validity of PSNR in image/video quality assessment. *Electron. Lett.* 44 800–801. 10.1049/el:20080522

[B40] Jin K. Huang X. Zhou J. Li Y. Yan Y. Sun Y. (2022). Fives: A fundus image dataset for artificial Intelligence based vessel segmentation. *Sci. Data* 9:475. 10.1038/s41597-022-01564-3 35927290 PMC9352679

[B41] Jin Q. Cui H. Sun C. Meng Z. Wei L. Su R. (2021). Domain adaptation based self-correction model for COVID-19 infection segmentation in CT images. *Expert Syst. Appl.* 176:114848. 10.1016/j.eswa.2021.114848 33746369 PMC7954643

[B42] Juang C.-F. Hung C.-W. Hsu C.-H. (2013). Rule-based cooperative continuous ant colony optimization to improve the accuracy of fuzzy system design. *IEEE Trans. Fuzzy Syst.* 22 723–735. 10.1109/TFUZZ.2013.2272480

[B43] Kalyani R. Sathya P. D. Sakthivel V. P. (2020). Trading strategies for image segmentation using multilevel thresholding aided with minimum cross entropy. *Eng. Sci. Technol. Int. J.* 23 1327–1341. 10.1016/j.jestch.2020.07.007

[B44] Kanne J. P. (2020). Chest CT findings in 2019 novel coronavirus (2019-nCoV) infections from Wuhan. China: Key points for the radiologist. *Radiology* 295 16–17. 10.1148/radiol.2020200241 32017662 PMC7233362

[B45] Kapur J. N. Sahoo P. K. Wong A. K. V. (1985). A new method for gray-level picture thresholding using the entropy of the histogram. *Comput. Vis. Graph. Image Proc.* 29 273–285. 10.1016/0734-189X(85)90125-2

[B46] Karakonstantis I. Vlachos A. (2018). Hybrid ant colony optimization for continuous domains for solving emission and economic dispatch problems. *J. Inform. Optim. Sci.* 39 651–671. 10.1080/02522667.2017.1385162

[B47] Kennedy J. Eberhart R. (1995). “Particle swarm optimization,” in *Proceedings of ICNN’95 - international conference on neural networks, 27 Nov.-1 Dec. 1995 1995, 4, 1942-1948*, Vol. 4 (Perth, WA: IEEE), 10.1109/ICNN.1995.488968

[B48] Kotte S. Pullakura R. K. Injeti S. K. (2018). Optimal multilevel thresholding selection for brain MRI image segmentation based on adaptive wind driven optimization. *Measurement* 130 340–361. 10.1016/j.measurement.2018.08.007

[B49] Kumar A. Thakur M. Mittal G. (2018). A new ants interaction scheme for continuous optimization problems. *Int. J. Syst. Assur. Eng. Manag.* 9 784–801. 10.1007/s13198-017-0651-3

[B50] Kumar N. Hussain I. Singh B. Panigrahi B. K. (2017). Single sensor-based mppt of partially shaded pv system for battery charging by using cauchy and gaussian sine cosine optimization. *IEEE Trans. Energy Convers.* 32 983–992. 10.1109/tec.2017.2669518

[B51] Li Y. Bai X. Jiao L. Xue Y. (2017). Partitioned-cooperative quantum-behaved particle swarm optimization based on multilevel thresholding applied to medical image segmentation. *Appl. Soft Comput.* 56 345–356. 10.1016/j.asoc.2017.03.018

[B52] Li Y. Zhang Y. Cui W. Lei B. Kuang X. Zhang T. (2022). Dual encoder-based dynamic-channel graph convolutional network with edge enhancement for retinal vessel segmentation. *IEEE Trans. Med. Imaging* 41 1975–1989. 10.1109/TMI.2022.3151666 35167444

[B53] Liang H. Liu Y. Shen Y. Li F. Man Y. (2018). A Hybrid bat algorithm for economic dispatch with random wind power. *IEEE Trans. Power Syst.* 33 5052–5061. 10.1109/TPWRS.2018.2812711

[B54] Liang J. J. Qin A. K. Suganthan P. N. Baskar S. (2006). Comprehensive learning particle swarm optimizer for global optimization of multimodal functions. *IEEE Trans. Evol. Comput.* 10 281–295. 10.1109/TEVC.2005.857610

[B55] Liang J. Qiao K. J. Yu K. J. Qu B. Y. Yue C. T. Guo W. F. (2022). “Utilizing the relationship between unconstrained and constrained pareto fronts for constrained multiobjective optimization,” in *IEEE transactions on cybernetics.* (Piscataway, NJ: IEEE), 1–14. 10.1109/TCYB.2022.3163759 35427231

[B56] Liu L. Dai Y. Gao J. (2014). Ant colony optimization algorithm for continuous domains based on position distribution model of ant colony foraging. *Sci. World J.* 2014:8539. 10.1155/2014/428539 24955402 PMC4037618

[B57] Liu Y. Heidari A. Cai Z. Liang G. Chen H. Pan Z. (2022b). Simulated annealing-based dynamic step shuffled frog leaping algorithm: Optimal performance design and feature selection. *Neurocomputing* 503 325–362. 10.1016/j.neucom.2022.06.075

[B58] Liu H. Liu M. Li D. Zheng W. Yin L. Wang R. (2022a). Recent advances in pulse-coupled neural networks with applications in image processing. *Electronics* 11:3264. 10.3390/electronics11203264

[B59] Liu Z. Su W. Ao J. Wang M. Jiang Q. He J. (2022c). Instant diagnosis of gastroscopic biopsy via deep-learned single-shot femtosecond stimulated Raman histology. *Nat. Commun.* 13:4050. 10.1038/s41467-022-31339-8 35831299 PMC9279377

[B60] Luo J. Chen H. Heidari A. A. Xu Y. Zhang Q. Li C. (2019). Multi-strategy boosted mutative whale-inspired optimization approaches. *Appl. Math. Model.* 73 109–123. 10.1016/j.apm.2019.03.046

[B61] Luo J. Chen H. Xu Y. Huang H. Zhao X. (2018). An improved grasshopper optimization algorithm with application to financial stress prediction. *Appl. Math. Model.* 64 654–668. 10.1016/j.apm.2018.07.044

[B62] Lv J. Li G. Tong X. Chen W. Huang J. Wang C. (2021). Transfer learning enhanced generative adversarial networks for multi-channel MRI reconstruction. *Comput. Biol. Med.* 134:104504. 10.1016/j.compbiomed.2021.104504 34062366

[B63] Manikandan S. Ramar K. Willjuice Iruthayarajan M. Srinivasagan K. G. (2014). Multilevel thresholding for segmentation of medical brain images using real coded genetic algorithm. *Measurement* 47 558–568. 10.1016/j.measurement.2013.09.031

[B64] Mirjalili S. (2015). Moth-flame optimization algorithm: A novel nature-inspired heuristic paradigm. *Knowl. Based Syst.* 89 228–249. 10.1016/j.knosys.2015.07.006

[B65] Mirjalili S. (2016). SCA: A Sine Cosine Algorithm for solving optimization problems. *Knowl. Based Syst.* 96 120–133. 10.1016/j.knosys.2015.12.022

[B66] Mirjalili S. Lewis A. (2016). The whale optimization algorithm. *Adv. Eng. Softw.* 95 51–67. 10.1016/j.advengsoft.2016.01.008

[B67] Mirjalili S. Mirjalili S. M. Hatamlou A. (2016). Multi-Verse Optimizer: A nature-inspired algorithm for global optimization. *Neural Comput. Appl.* 27 495–513. 10.1007/s00521-015-1870-7

[B68] Mirjalili S. Mirjalili S. M. Lewis A. (2014). Grey wolf optimizer. *Adv. Eng. Softw.* 69 46–61. 10.1016/j.advengsoft.2013.12.007

[B69] Pham T. X. Siarry P. Oulhadj H. (2019). A multi-objective optimization approach for brain MRI segmentation using fuzzy entropy clustering and region-based active contour methods. *Magn. Reson. Imaging* 61 41–65. 10.1016/j.mri.2019.05.009 31108153

[B70] Pun T. (1981). Entropic thresholding, a new approach. *Comput. Graph. Image Proc.* 16 210–239. 10.1016/0146-664X(81)90038-1

[B71] Qin X. Ban Y. Wu P. Yang B. Liu S. Yin L. (2022). Improved image fusion method based on sparse decomposition. *Electronics* 11:2321. 10.3390/electronics11152321

[B72] Qu C. Zeng Z. Dai J. Yi Z. He W. (2018). A modified sine-cosine algorithm based on neighborhood search and greedy levy mutation, (in eng). *Comput. Intell. Neurosci.* 2018:4231647. 10.1155/2018/4231647 30073023 PMC6057408

[B73] Radha R. Gopalakrishnan R. (2020). A medical analytical system using intelligent fuzzy level set brain image segmentation based on improved quantum particle swarm optimization. *Microproc. Microsyst.* 79:103283. 10.1016/j.micpro.2020.103283

[B74] Rakesh P. S. Mahesh D. S. (2021). Nodule segmentation of lung CT image for medical applications. *Glob. Trans. Proc.* 2 80–83. 10.1016/j.gltp.2021.01.011

[B75] Rodríguez-Esparza E. Zanella-Calzada L. A. Oliva D. Heidari A. A. Zaldívar D. Cisneros M. A. P. (2020). An efficient Harris hawks-inspired image segmentation method. *Expert Syst. Appl.* 155:113428. 10.1016/j.eswa.2020.113428

[B76] Shahraki H. Zahiri S.-H. (2017). Ant colony optimization and decision function estimation. *Intell. Decis. Technol.* 11 71–78. 10.3233/idt-160278

[B77] Sheela C. J. J. Suganthi G. (2019). Automatic brain tumor segmentation from MRI using greedy snake model and fuzzy c-means optimization. *J. King Saud Univ.* 34 557–566. 10.1016/j.jksuci.2019.04.006

[B78] Shorfuzzaman M. Hossain M. S. (2021). Meta COVID: A Siamese neural network framework with contrastive loss for n-shot diagnosis of COVID-19 patients. *Pattern Recognit.* 113:107700. 10.1016/j.patcog.2020.107700 33100403 PMC7568501

[B79] Socha K. Dorigo M. (2008). Ant colony optimization for continuous domains. *Eur. J. Operat. Res.* 185 1155–1173. 10.1016/j.ejor.2006.06.046

[B80] Storn R. Price K. (1997). Differential evolution – a simple and efficient heuristic for global optimization over continuous spaces. *J. Glob. Optim.* 11 341–359. 10.1023/a:1008202821328

[B81] Sun X. Cao X. Zeng B. Zhai Q. Guan X. (2022). “Multistage dynamic planning of integrated hydrogen-electrical microgrids under multiscale uncertainties,” in *IEEE transactions on smart grid*, (Piscataway, NJ: IEEE), 1–1. 10.1109/TSG.2022.3232545

[B82] Tarkhaneh O. Shen H. (2019). An adaptive differential evolution algorithm to optimal multi-level thresholding for MRI brain image segmentation. *Expert Syst. Appl.* 138:112820. 10.1016/j.eswa.2019.07.037

[B83] Tu J. Chen H. Liu J. Asghar Heidari A. Zhang X. Wang M. (2020). Evolutionary biogeography-based Whale optimization methods with communication structure: Towards measuring the balance. *Knowl. Based Sys.* 212:106642. 10.1016/j.knosys.2020.106642

[B84] Tu J. Chen H. Liu J. Asghar Heidari A. Zhang X. Wang M. (2021). Evolutionary biogeography-based whale optimization methods with communication structure: Towards measuring the balance. *Knowl. Based Syst.* 212:06642.

[B85] Tubishat M. Abushariah M. A. M. Idris N. Aljarah I. (2019). Improved whale optimization algorithm for feature selection in Arabic sentiment analysis. *Appl. Intell.* 49 1688–1707. 10.1007/s10489-018-1334-8

[B86] Vaze R. Deshmukh N. Kumar R. Saxena A. (2021). Development and application of Quantum Entanglement inspired Particle Swarm Optimization. *Knowl. Based Syst.* 219:106859. 10.1016/j.knosys.2021.106859

[B87] Verma H. Verma D. Tiwari P. K. (2021). A population based hybrid FCM-PSO algorithm for clustering analysis and segmentation of brain image. *Expert Syst. Appl.* 167:114121. 10.1016/j.eswa.2020.114121

[B88] Wang S. Chen Z. You S. Wang B. Shen Y. Lei B. (2022). Brain stroke lesion segmentation using consistent perception generative adversarial network. *Neural Comput. Appl.* 34 8657–8669. 10.1007/s00521-021-06816-8

[B89] Wang S. Wang B. Zhang Z. Heidari A. A. Chen H. (2023). Class-aware sample reweighting optimal transport for multi-source domain adaptation. *Neurocomputing* 523 213–223. 10.1016/j.neucom.2022.12.048

[B90] Wang X. Chen H. L. Heidari A. A. Zhang X. Xu J. Xu Y. T. (2020). Multi-population following behavior-driven fruit fly optimization: A Markov chain convergence proof and comprehensive analysis. *Knowl. Based Syst.* 210:106437. 10.1016/j.knosys.2020.106437

[B91] Wu B. Zhou J. Ji X. Yin Y. Shen X. (2020). An ameliorated teaching–learning-based optimization algorithm based study of image segmentation for multilevel thresholding using Kapur’s entropy and Otsu’s between class variance. *Inform. Sci.* 533 72–107. 10.1016/j.ins.2020.05.033

[B92] Wu M.-T. Hong T.-P. Lee C.-N. (2017). A dynamic-edge ACS algorithm for continuous variables problems. *Nat. Comput.* 16 339–352. 10.1007/s11047-015-9537-y

[B93] Wu Y. Ma W. Miao Q. Wang S. (2019). Multimodal continuous ant colony optimization for multisensor remote sensing image registration with local search. *Swarm Evol. Comput.* 47 89–95. 10.1016/j.swevo.2017.07.004

[B94] Wu X. Chen C. Zhong M. Wang J. Shi J. (2021a). The diagnosis of COVID-19 with deep active learning. *Med. Image Anal.* 68:101913. 10.1016/j.media.2020.101913 33285482 PMC7689310

[B95] Wu Z. Shen S. Li H. Zhou H. Lu C. (2021c). A basic framework for privacy protection in personalized information retrieval: An effective framework for user privacy protection. *J. Organ. End User Comput.* 33 1–26.

[B96] Wu Z. Shen S. Zhou H. Li H. Lu C. Zou D. (2021d). An effective approach for the protection of user commodity viewing privacy in e-commerce website. *Knowl. Based Syst.* 220:106952. 10.1016/j.knosys.2021.106952

[B97] Wu Z. Li G. Shen S. Lian X. Chen E. Xu G. (2021b). Constructing dummy query sequences to protect location privacy and query privacy in location-based services. *World Wide Web* 24 25–49. 10.1007/s11280-020-00830-x

[B98] Wu Z. Shen S. Lian X. Su X. Chen E. (2020). A dummy-based user privacy protection approach for text information retrieval. *Knowl. Based Syst.* 195:105679. 10.1016/j.knosys.2020.105679

[B99] Wu Z. Xie J. Shen S. Lin C. Xu G. Chen E. (2023). “A confusion method for the protection of user topic privacy in chinese keyword based book retrieval,” in *ACM transactions on asian and low-resource language information processing*, (New York, NY: ACM).

[B100] Wu Z. Xuan S. Xie J. Lin C. Lu C. (2022). How to ensure the confidentiality of electronic medical records on the cloud: A technical perspective. *Comput. Biol. Med.* 147:105726. 10.1016/j.compbiomed.2022.105726 35759991

[B101] Xie X. Zhong Z. Zhao W. Zheng C. Wang F. Liu J. (2020). Chest CT for typical coronavirus disease 2019 (COVID-19) pneumonia: Relationship to negative RT-PCR testing. *Radiology* 296 E41–E45. 32049601 10.1148/radiol.2020200343 PMC7233363

[B102] Xing Z. (2020). An improved emperor penguin optimization based multilevel thresholding for color image segmentation. *Knowl. Based Syst.* 194:105570. 10.1016/j.knosys.2020.105570

[B103] Xue Y. Cai X. Neri F. (2022a). A multi-objective evolutionary algorithm with interval based initialization and self-adaptive crossover operator for large-scale feature selection in classification. *Appl. Soft Comput.* 127:109420.

[B104] Xue Y. Tong Y. Neri F. (2022b). An ensemble of differential evolution and Adam for training feed-forward neural networks. *Inform. Sci.* 608 453–471. 10.1016/j.ins.2022.06.036

[B105] Xue Y. Xue B. Zhang M. (2019). Self-adaptive particle swarm optimization for large-scale feature selection in classification. *ACM Trans. Knowl. Discov. Data* 13 1–27.

[B106] Yan B. Li Y. Li L. Yang X. Li T. Yang G. (2022). Quantifying the impact of Pyramid Squeeze Attention mechanism and filtering approaches on Alzheimer’s disease classification. *Comput. Biol. Med.* 148:105944. 10.1016/j.compbiomed.2022.105944 35969934

[B107] Yang D. Zhu T. Wang S. Wang S. Xiong Z. (2022). LFRSNet: A robust light field semantic segmentation network combining contextual and geometric features. *Front. Environ. Sci.* 10:996513. 10.3389/fenvs.2022.996513

[B108] Yang X.-S. (2009). “Firefly algorithms for multimodal optimization,” in *Stochastic Algorithms: Foundations and Applications*, eds Watanabe O. Zeugmann T. (Berlin: Springer), 169–178.

[B109] Yang X.-S. (2010). “A new metaheuristic bat-inspired algorithm,” in *Nature inspired cooperative strategies for optimization (NICSO 2010)*, eds González J. R. Pelta D. A. Cruz C. Terrazas G. Krasnogor N. (Berlin: Springer), 65–74.

[B110] Yong J. He F. Li H. Zhou W. (2018). “A novel bat algorithm based on collaborative and Dynamic learning of opposite population,” in *2018 IEEE 22nd International Conference on Computer Supported Cooperative Work in Design ((CSCWD))*, (Nanjing: IEEE), 541–546.

[B111] You S. Lei B. Wang S. Chui C. K. Cheung A. C. Liu Y. (2022). Fine Perceptive GANs for Brain MR Image Super-Resolution in Wavelet Domain. *IEEE Trans. Neural Netw. Learn. Syst.* 10.1109/TNNLS.2022.3153088 [Epub ahead of print]. 35254996

[B112] Yu K. Zhang D. Liang J. Chen K. Yue C. Qiao K. (2022). “A correlation-guided layered prediction approach for evolutionary dynamic multiobjective optimization,” in *IEEE transactions on evolutionary computation* (Piscataway, NJ: IEEE), 1–1. 10.1109/TEVC.2022.3193287

[B113] Zhang B. Qi H. Sun S.-C. Ruan L.-M. Tan H.-P. (2016). A novel hybrid ant colony optimization and particle swarm optimization algorithm for inverse problems of coupled radiative and conductive heat transfer. *Therm. Sci.* 20 461–472. 10.2298/tsci131124023z

[B114] Zhang L. Zhang L. Mou X. Zhang D. (2011). FSIM: A feature similarity index for image quality assessment. *IEEE Trans. Image Proc.* 20 2378–2386. 10.1109/TIP.2011.2109730 21292594

[B115] Zhang Y. Liu R. Heidari A. A. Wang X. Chen Y. Wang M. (2021). Towards augmented kernel extreme learning models for bankruptcy prediction: Algorithmic behavior and comprehensive analysis. *Neurocomputing* 430 185–212.

[B116] Zhang Y. Liu R. Heidari A. Wang X. Chen Y. Wang M. (2020). Towards augmented kernel extreme learning models for bankruptcy prediction: Algorithmic behavior and comprehensive analysis. *Neurocomputing* 430 185–212. 10.1016/j.neucom.2020.10.038

[B117] Zhao C. Wang H. Chen H. Shi W. Feng Y. (2022). “JAMSNet: A remote pulse extraction network based on joint attention and multi-scale fusion,” in *IEEE Transactions on Circuits and Systems for Video Technology*, (Piscataway, NJ: IEEE), 1–1. 10.1109/TCSVT.2022.3227348

[B118] Zhao D. Liu L. Yu F. Heidari A. Wang M. Liang G. (2021a). Chaotic random spare ant colony optimization for multi-threshold image segmentation of 2D Kapur entropy. *Knowl. Based Syst.* 216:106510. 10.1016/j.knosys.2020.106510

[B119] Zhao D. Liu L. Yu F. Heidari A. A. Wang M. Oliva D. (2021b). Ant colony optimization with horizontal and vertical crossover search: Fundamental visions for multi-threshold image segmentation. *Expert Syst. Appl.* 167:114122. 10.1016/j.eswa.2020.114122

[B120] Zhou W. Bovik A. C. Sheikh H. R. Simoncelli E. P. (2004). Image quality assessment: From error visibility to structural similarity. *IEEE Trans. Image Proc.* 13 600–612. 10.1109/TIP.2003.819861 15376593

[B121] Zhu A. Xu C. Li Z. Wu J. Liu Z. (2015). Hybridizing grey wolf optimization with differential evolution for global optimization and test scheduling for 3D stacked SoC. *J. Syst. Eng. Electron.* 26 317–328. 10.1109/jsee.2015.00037

