# Supplementary material for: An enhanced ant colony optimizer with Cauchy-Gaussian fusion and novel movement strategy for multi-threshold COVID-19 X-ray image segmentation
Source: Front Neuroinform. 2023 Mar 17;17:1126783. doi: 10.3389/fninf.2023.1126783 (PMC10064065; doi:10.3389/fninf.2023.1126783)
Supplement: Supplementary file 1 [file Data_Sheet_1.docx]

# Appendix A

**Table A. 1.** The relevant comparison results of four variants

| Fun | Item | MGACO | MACO | GACO | ACOR |
| --- | --- | --- | --- | --- | --- |
| F1 | AVG | 4.892E+05 | 1.774E+06 | **3.869E+05** | 6.289E+06 |
|  | STD | 3.372E+05 | 1.555E+06 | **3.027E+05** | 7.571E+06 |
| F2 | AVG | 1.128E+04 | 1.018E+04 | **9.318E+03** | 8.627E+07 |
|  | STD | **1.086E+04** | 1.136E+04 | 1.089E+04 | 3.998E+08 |
| F3 | AVG | 3.609E+02 | **3.451E+02** | 1.070E+03 | 8.474E+03 |
|  | STD | 5.594E+01 | **2.295E+01** | 1.091E+03 | 8.153E+03 |
| F4 | AVG | **4.144E+02** | 4.487E+02 | 5.218E+02 | 4.843E+02 |
|  | STD | **3.191E+01** | 4.227E+01 | 6.116E+01 | 4.868E+01 |
| F5 | AVG | 5.201E+02 | **5.201E+02** | 5.209E+02 | 5.209E+02 |
|  | STD | 5.884E-02 | 7.505E-02 | **5.310E-02** | 6.666E-02 |
| F6 | AVG | 6.101E+02 | 6.119E+02 | **6.075E+02** | 6.121E+02 |
|  | STD | 3.529E+00 | **3.394E+00** | 3.480E+00 | 3.420E+00 |
| F7 | AVG | 7.000E+02 | 7.000E+02 | **7.000E+02** | 7.043E+02 |
|  | STD | 1.202E-02 | 2.549E-02 | **4.561E-03** | 6.719E+00 |
| F8 | AVG | 8.362E+02 | **8.273E+02** | 8.457E+02 | 8.648E+02 |
|  | STD | 9.227E+00 | **6.534E+00** | 2.737E+01 | 2.318E+01 |
| F9 | AVG | **9.470E+02** | 9.530E+02 | 1.080E+03 | 1.021E+03 |
|  | STD | 1.683E+01 | **1.316E+01** | 2.868E+01 | 5.764E+01 |
| F10 | AVG | 2.049E+03 | **1.890E+03** | 2.965E+03 | 3.113E+03 |
|  | STD | 3.673E+02 | **2.972E+02** | 5.797E+02 | 4.967E+02 |
| F11 | AVG | **3.172E+03** | 3.434E+03 | 7.295E+03 | 5.119E+03 |
|  | STD | **6.455E+02** | 7.145E+02 | 1.026E+03 | 2.057E+03 |
| F12 | AVG | 1.200E+03 | **1.200E+03** | 1.202E+03 | 1.202E+03 |
|  | STD | 3.035E-01 | **2.327E-01** | 2.675E-01 | 3.968E-01 |
| F13 | AVG | **1.300E+03** | 1.300E+03 | 1.300E+03 | 1.300E+03 |
|  | STD | **6.764E-02** | 8.889E-02 | 7.204E-02 | 1.265E-01 |
| F14 | AVG | 1.400E+03 | 1.401E+03 | **1.400E+03** | 1.401E+03 |
|  | STD | 1.595E-01 | 2.240E-01 | **1.485E-01** | 2.904E-01 |
| F15 | AVG | 1.506E+03 | **1.506E+03** | 1.515E+03 | 1.528E+03 |
|  | STD | 1.585E+00 | 1.813E+00 | **1.548E+00** | 3.130E+01 |
| F16 | AVG | **1.611E+03** | 1.611E+03 | 1.612E+03 | 1.612E+03 |
|  | STD | 8.653E-01 | 6.838E-01 | 5.040E-01 | **4.903E-01** |
| F17 | AVG | 7.405E+04 | 1.249E+05 | **6.881E+04** | 1.238E+05 |
|  | STD | **3.193E+04** | 9.858E+04 | 6.138E+04 | 2.453E+05 |
| F18 | AVG | **6.636E+03** | 7.331E+03 | 6.856E+05 | 1.251E+04 |
|  | STD | 6.187E+03 | **6.180E+03** | 3.723E+06 | 8.794E+03 |
| F19 | AVG | **1.911E+03** | 1.915E+03 | 1.917E+03 | 1.918E+03 |
|  | STD | **3.388E+00** | 2.090E+01 | 2.361E+01 | 3.653E+01 |
| F20 | AVG | 2.188E+03 | **2.168E+03** | 2.672E+03 | 1.368E+04 |
|  | STD | 7.316E+01 | **6.921E+01** | 5.967E+02 | 3.888E+04 |
| F21 | AVG | 6.364E+04 | 6.660E+04 | **4.810E+04** | 7.855E+04 |
|  | STD | 4.558E+04 | 5.095E+04 | **2.969E+04** | 5.180E+04 |
| F22 | AVG | 2.515E+03 | 2.551E+03 | **2.478E+03** | 2.552E+03 |
|  | STD | 1.962E+02 | 1.749E+02 | **1.634E+02** | 2.392E+02 |
| F23 | AVG | **2.500E+03** | 2.615E+03 | 2.500E+03 | 2.617E+03 |
|  | STD | **0.000E+00** | 1.683E-12 | 0.000E+00 | 2.577E+00 |
| F24 | AVG | **2.600E+03** | 2.637E+03 | 2.600E+03 | 2.642E+03 |
|  | STD | **0.000E+00** | 6.990E+00 | 0.000E+00 | 4.737E+00 |
| F25 | AVG | **2.700E+03** | 2.706E+03 | 2.700E+03 | 2.707E+03 |
|  | STD | **0.000E+00** | 2.068E+00 | 0.000E+00 | 2.676E+00 |
| F26 | AVG | **2.700E+03** | 2.740E+03 | 2.724E+03 | 2.757E+03 |
|  | STD | **6.639E-02** | 8.959E+01 | 4.288E+01 | 1.045E+02 |
| F27 | AVG | 2.911E+03 | 3.316E+03 | **2.900E+03** | 3.410E+03 |
|  | STD | 6.129E+01 | 1.137E+02 | **0.000E+00** | 1.163E+02 |
| F28 | AVG | **3.000E+03** | 3.777E+03 | 3.000E+03 | 3.840E+03 |
|  | STD | 2.533E-13 | 1.149E+02 | **0.000E+00** | 1.923E+02 |
| F29 | AVG | **3.123E+03** | 2.246E+06 | 1.979E+05 | 9.223E+05 |
|  | STD | **3.657E+01** | 4.158E+06 | 8.112E+04 | 2.797E+06 |
| F30 | AVG | **3.864E+03** | 7.712E+03 | 2.324E+04 | 8.783E+03 |
|  | STD | **2.273E+02** | 6.571E+03 | 2.300E+04 | 4.549E+03 |
|  | +/-/= | ~ | 12/2/16 | 15/2/13 | 24/0/6 |
|  | Mean | **1.57** | 2.33 | 2.20 | 3.77 |
|  | Rank | **1** | 3 | 2 | 4 |

**Table A. 2.** Scalability test results at dimensions 10, 30, 50 and 100

|  |  | 10 | | 30 | | 50 | | 100 | |
| --- | --- | --- | --- | --- | --- | --- | --- | --- | --- |
| Fun | Item | MGACO | ACOR | MGACO | ACOR | MGACO | ACOR | MGACO | ACOR |
| F1 | AVG | **1.462E+06** | 4.078E+06 | **3.989E+05** | 6.179E+06 | **2.702E+05** | 4.692E+06 | **1.241E+05** | 5.956E+06 |
|  | STD | **7.412E+05** | 4.158E+06 | **2.321E+05** | 8.703E+06 | **1.572E+05** | 5.754E+06 | **8.892E+04** | 9.093E+06 |
| F2 | AVG | **1.347E+04** | 7.601E+07 | **9.979E+03** | 1.212E+08 | **9.186E+03** | 6.409E+07 | 5.787E+03 | **4.466E+03** |
|  | STD | **1.407E+04** | 4.162E+08 | **1.149E+04** | 6.638E+08 | **9.986E+03** | 3.510E+08 | **7.329E+03** | 7.436E+03 |
| F3 | AVG | **8.707E+02** | 1.550E+04 | **3.440E+02** | 1.068E+04 | **3.178E+02** | 1.510E+04 | **3.035E+02** | 1.268E+04 |
|  | STD | **2.961E+02** | 1.476E+04 | **3.400E+01** | 1.554E+04 | **2.549E+01** | 2.531E+04 | **3.791E+00** | 1.826E+04 |
| F4 | AVG | **4.617E+02** | 4.992E+02 | **4.224E+02** | 4.812E+02 | **4.066E+02** | 4.703E+02 | **4.165E+02** | 4.841E+02 |
|  | STD | **4.072E+01** | 6.455E+01 | **3.133E+01** | 5.991E+01 | **1.692E+01** | 4.208E+01 | **3.035E+01** | 9.251E+01 |
| F5 | AVG | **5.204E+02** | 5.210E+02 | **5.201E+02** | 5.209E+02 | **5.200E+02** | 5.209E+02 | **5.200E+02** | 5.209E+02 |
|  | STD | 1.330E-01 | **6.494E-02** | 6.399E-02 | **6.336E-02** | **2.814E-02** | 6.184E-02 | **8.583E-03** | 5.835E-02 |
| F6 | AVG | **6.094E+02** | 6.126E+02 | **6.100E+02** | 6.124E+02 | **6.089E+02** | 6.125E+02 | **6.093E+02** | 6.132E+02 |
|  | STD | 3.682E+00 | **2.956E+00** | 4.047E+00 | **2.584E+00** | 3.614E+00 | **3.037E+00** | 3.535E+00 | **2.953E+00** |
| F7 | AVG | **7.000E+02** | 7.059E+02 | **7.000E+02** | 7.066E+02 | **7.000E+02** | 7.032E+02 | **7.000E+02** | 7.032E+02 |
|  | STD | **2.989E-02** | 1.454E+01 | **1.197E-02** | 1.880E+01 | **7.963E-03** | 7.636E+00 | **1.329E-02** | 5.960E+00 |
| F8 | AVG | **8.442E+02** | 8.652E+02 | **8.383E+02** | 8.616E+02 | **8.308E+02** | 8.687E+02 | **8.249E+02** | 8.601E+02 |
|  | STD | **1.158E+01** | 1.759E+01 | **9.344E+00** | 1.908E+01 | **6.890E+00** | 1.869E+01 | **4.678E+00** | 1.543E+01 |
| F9 | AVG | **9.556E+02** | 1.080E+03 | **9.454E+02** | 1.019E+03 | **9.453E+02** | 1.013E+03 | **9.400E+02** | 9.737E+02 |
|  | STD | **1.368E+01** | 5.718E+01 | **1.391E+01** | 5.888E+01 | **1.342E+01** | 6.713E+01 | **9.780E+00** | 3.889E+01 |
| F10 | AVG | **2.479E+03** | 3.288E+03 | **2.203E+03** | 2.988E+03 | **1.977E+03** | 3.063E+03 | **1.879E+03** | 3.204E+03 |
|  | STD | 5.276E+02 | **5.081E+02** | **3.863E+02** | 5.307E+02 | **3.339E+02** | 4.190E+02 | **2.378E+02** | 5.738E+02 |
| F11 | AVG | **3.465E+03** | 6.231E+03 | **3.163E+03** | 4.520E+03 | **3.131E+03** | 4.159E+03 | **2.955E+03** | 3.657E+03 |
|  | STD | **6.506E+02** | 2.113E+03 | **6.790E+02** | 1.966E+03 | **5.276E+02** | 1.896E+03 | **5.249E+02** | 1.136E+03 |
| F12 | AVG | **1.201E+03** | 1.203E+03 | **1.200E+03** | 1.202E+03 | **1.200E+03** | 1.202E+03 | **1.200E+03** | 1.202E+03 |
|  | STD | 4.750E-01 | **2.965E-01** | **2.612E-01** | 3.282E-01 | **2.043E-01** | 3.693E-01 | **1.851E-01** | 2.844E-01 |
| F13 | AVG | **1.300E+03** | 1.301E+03 | **1.300E+03** | 1.300E+03 | **1.300E+03** | 1.300E+03 | **1.300E+03** | 1.300E+03 |
|  | STD | **1.077E-01** | 3.920E-01 | **6.113E-02** | 1.104E-01 | **6.077E-02** | 1.395E-01 | **6.229E-02** | 1.401E-01 |
| F14 | AVG | **1.400E+03** | 1.401E+03 | **1.400E+03** | 1.401E+03 | **1.400E+03** | 1.401E+03 | **1.400E+03** | 1.401E+03 |
|  | STD | **1.990E-01** | 7.139E-01 | **1.553E-01** | 2.357E-01 | **1.160E-01** | 7.533E-01 | **1.297E-01** | 2.606E-01 |
| F15 | AVG | **1.508E+03** | 1.547E+03 | **1.506E+03** | 1.995E+03 | **1.505E+03** | 1.572E+03 | **1.504E+03** | 1.675E+03 |
|  | STD | **2.330E+00** | 9.766E+01 | **1.704E+00** | 2.422E+03 | **1.155E+00** | 1.340E+02 | **1.253E+00** | 4.096E+02 |
| F16 | AVG | **1.611E+03** | 1.612E+03 | **1.610E+03** | 1.612E+03 | **1.610E+03** | 1.612E+03 | **1.610E+03** | 1.611E+03 |
|  | STD | 5.862E-01 | **3.759E-01** | 6.673E-01 | **4.751E-01** | 8.871E-01 | **4.093E-01** | 6.988E-01 | **5.962E-01** |
| F17 | AVG | **2.240E+05** | 4.048E+05 | **7.415E+04** | 1.973E+05 | **3.390E+04** | 2.177E+05 | **2.127E+04** | 4.207E+05 |
|  | STD | **1.847E+05** | 5.367E+05 | **3.566E+04** | 4.309E+05 | **2.168E+04** | 3.636E+05 | **1.571E+04** | 9.052E+05 |
| F18 | AVG | **7.218E+03** | 7.641E+03 | **5.954E+03** | 4.965E+04 | **6.543E+03** | 7.728E+03 | 9.037E+03 | **6.978E+03** |
|  | STD | 7.090E+03 | **5.766E+03** | **3.850E+03** | 2.133E+05 | **5.614E+03** | 6.199E+03 | 7.984E+03 | **4.291E+03** |
| F19 | AVG | **1.913E+03** | 1.933E+03 | **1.909E+03** | 1.923E+03 | **1.909E+03** | 1.915E+03 | **1.911E+03** | 1.914E+03 |
|  | STD | **1.342E+01** | 4.093E+01 | **2.772E+00** | 2.613E+01 | **3.132E+00** | 1.864E+01 | **1.420E+01** | 1.463E+01 |
| F20 | AVG | **2.844E+03** | 1.371E+04 | **2.173E+03** | 4.849E+03 | **2.108E+03** | 3.536E+03 | **2.053E+03** | 5.843E+03 |
|  | STD | **8.877E+02** | 1.567E+04 | **7.356E+01** | 7.414E+03 | **4.583E+01** | 1.923E+03 | **2.158E+01** | 8.194E+03 |
| F21 | AVG | 1.767E+05 | **1.678E+05** | **5.610E+04** | 1.342E+05 | **4.212E+04** | 5.976E+04 | **2.796E+04** | 5.028E+04 |
|  | STD | **1.407E+05** | 1.425E+05 | **3.646E+04** | 2.291E+05 | **3.368E+04** | 1.166E+05 | **1.680E+04** | 7.539E+04 |
| F22 | AVG | 2.556E+03 | **2.552E+03** | **2.486E+03** | 2.564E+03 | **2.514E+03** | 2.529E+03 | **2.481E+03** | 2.595E+03 |
|  | STD | **2.113E+02** | 2.459E+02 | **1.983E+02** | 2.351E+02 | **1.840E+02** | 2.177E+02 | **1.454E+02** | 2.026E+02 |
| F23 | AVG | **2.500E+03** | 2.618E+03 | **2.500E+03** | 2.620E+03 | **2.500E+03** | 2.617E+03 | **2.500E+03** | 2.618E+03 |
|  | STD | **8.444E-14** | 4.940E+00 | **0.000E+00** | 6.707E+00 | **0.000E+00** | 3.803E+00 | **0.000E+00** | 4.612E+00 |
| F24 | AVG | **2.600E+03** | 2.643E+03 | **2.600E+03** | 2.642E+03 | **2.600E+03** | 2.641E+03 | **2.600E+03** | 2.640E+03 |
|  | STD | **8.444E-14** | 6.175E+00 | **0.000E+00** | 6.678E+00 | **0.000E+00** | 6.166E+00 | **0.000E+00** | 7.269E+00 |
| F25 | AVG | **2.700E+03** | 2.707E+03 | **2.700E+03** | 2.707E+03 | **2.700E+03** | 2.708E+03 | **2.700E+03** | 2.707E+03 |
|  | STD | **0.000E+00** | 3.135E+00 | **0.000E+00** | 2.982E+00 | **0.000E+00** | 4.199E+00 | **0.000E+00** | 3.338E+00 |
| F26 | AVG | **2.704E+03** | 2.734E+03 | **2.700E+03** | 2.766E+03 | **2.700E+03** | 2.722E+03 | **2.700E+03** | 2.759E+03 |
|  | STD | **1.819E+01** | 7.481E+01 | **5.381E-02** | 1.045E+02 | **6.568E-02** | 5.353E+01 | **4.769E-02** | 9.793E+01 |
| F27 | AVG | **2.912E+03** | 3.407E+03 | **2.900E+03** | 3.424E+03 | **2.900E+03** | 3.427E+03 | **2.900E+03** | 3.390E+03 |
|  | STD | **6.673E+01** | 7.960E+01 | **0.000E+00** | 9.854E+01 | **0.000E+00** | 7.169E+01 | **0.000E+00** | 1.026E+02 |
| F28 | AVG | **3.027E+03** | 3.886E+03 | **3.013E+03** | 3.821E+03 | **3.000E+03** | 3.895E+03 | **3.000E+03** | 3.887E+03 |
|  | STD | **6.940E+01** | 2.055E+02 | **4.827E+01** | 1.701E+02 | **0.000E+00** | 2.015E+02 | **0.000E+00** | 1.810E+02 |
| F29 | AVG | **3.125E+03** | 3.976E+06 | **3.117E+03** | 2.743E+06 | **3.113E+03** | 1.358E+06 | **3.117E+03** | 1.878E+06 |
|  | STD | **2.496E+01** | 5.027E+06 | **1.821E+01** | 4.269E+06 | **1.991E+00** | 3.171E+06 | **1.861E+01** | 3.852E+06 |
| F30 | AVG | **3.966E+03** | 8.398E+03 | **3.947E+03** | 9.460E+03 | **3.899E+03** | 1.033E+04 | **3.843E+03** | 8.678E+03 |
|  | STD | **5.091E+02** | 2.954E+03 | **3.242E+02** | 5.942E+03 | **2.792E+02** | 8.081E+03 | **1.650E+02** | 2.905E+03 |
|  | +/-/= | ~ | 24/0/6 | ~ | 26/0/4 | ~ | 25/0/5 | ~ | 26/0/4 |
|  | Mean | 1.07 | 1.93 | 1 | 2 | 1 | 2 | 1.07 | 1.93 |
|  | Rank | 1 | 2 | 1 | 2 | 1 | 2 | 1 | 2 |

**Table A. 3.** The comparison results of MGACO with other conventional methods

| Fun | Item | MGACO | MVO | ACOR | BA | DE | FA | GWO | MFO | PSO | SCA | WOA |
| --- | --- | --- | --- | --- | --- | --- | --- | --- | --- | --- | --- | --- |
| F1 | AVG | **3.769E+05** | 2.356E+06 | 6.495E+06 | 7.155E+05 | 1.917E+07 | 2.509E+08 | 7.500E+07 | 7.707E+07 | 8.246E+06 | 2.279E+08 | 3.147E+07 |
|  | STD | **3.047E+05** | 7.287E+05 | 7.568E+06 | 4.188E+05 | 4.876E+06 | 5.709E+07 | 6.932E+07 | 1.314E+08 | 2.406E+06 | 6.810E+07 | 1.532E+07 |
| F2 | AVG | 1.527E+04 | 1.666E+04 | 3.478E+08 | 5.792E+05 | **2.856E+03** | 1.514E+10 | 2.432E+09 | 1.199E+10 | 1.446E+08 | 1.601E+10 | 2.324E+06 |
|  | STD | 1.049E+04 | 1.206E+04 | 9.666E+08 | 3.679E+05 | **6.817E+03** | 2.242E+09 | 1.938E+09 | 7.873E+09 | 1.890E+07 | 2.778E+09 | 1.355E+06 |
| F3 | AVG | **3.449E+02** | 4.010E+02 | 9.414E+03 | 5.186E+02 | 4.077E+02 | 6.512E+04 | 2.761E+04 | 1.048E+05 | 9.756E+02 | 3.654E+04 | 4.024E+04 |
|  | STD | **2.633E+01** | 3.557E+01 | 1.346E+04 | 3.567E+02 | 1.131E+02 | 9.492E+03 | 8.341E+03 | 4.706E+04 | 1.658E+02 | 6.516E+03 | 1.973E+04 |
| F4 | AVG | **4.208E+02** | 4.879E+02 | 4.764E+02 | 4.253E+02 | 5.022E+02 | 1.556E+03 | 6.204E+02 | 1.564E+03 | 4.664E+02 | 1.375E+03 | 5.903E+02 |
|  | STD | 3.056E+01 | 2.901E+01 | 3.560E+01 | 3.773E+01 | **2.724E+01** | 1.550E+02 | 8.745E+01 | 1.025E+03 | 3.482E+01 | 2.155E+02 | 5.613E+01 |
| F5 | AVG | 5.201E+02 | **5.201E+02** | 5.209E+02 | 5.210E+02 | 5.206E+02 | 5.210E+02 | 5.209E+02 | 5.203E+02 | 5.209E+02 | 5.209E+02 | 5.204E+02 |
|  | STD | 7.166E-02 | 4.827E-02 | 5.395E-02 | 6.485E-02 | 3.825E-02 | 5.557E-02 | 1.248E-01 | 1.869E-01 | **3.542E-02** | 4.858E-02 | 1.833E-01 |
| F6 | AVG | **6.093E+02** | 6.104E+02 | 6.116E+02 | 6.344E+02 | 6.194E+02 | 6.339E+02 | 6.141E+02 | 6.250E+02 | 6.233E+02 | 6.336E+02 | 6.347E+02 |
|  | STD | 2.951E+00 | 3.242E+00 | 2.714E+00 | 3.387E+00 | 1.576E+00 | **1.073E+00** | 2.739E+00 | 3.813E+00 | 3.852E+00 | 2.577E+00 | 3.126E+00 |
| F7 | AVG | 7.000E+02 | 7.000E+02 | 7.035E+02 | 7.006E+02 | **7.000E+02** | 8.347E+02 | 7.202E+02 | 7.975E+02 | 7.023E+02 | 8.293E+02 | 7.010E+02 |
|  | STD | 2.437E-02 | 2.290E-02 | 7.757E+00 | 1.661E-01 | **2.993E-13** | 1.317E+01 | 1.452E+01 | 6.423E+01 | 1.572E-01 | 2.411E+01 | 6.094E-02 |
| F8 | AVG | 8.343E+02 | 8.732E+02 | 8.597E+02 | 1.012E+03 | **8.011E+02** | 1.026E+03 | 8.814E+02 | 9.487E+02 | 9.761E+02 | 1.039E+03 | 9.939E+02 |
|  | STD | 9.808E+00 | 1.400E+01 | 1.631E+01 | 4.052E+01 | **1.046E+00** | 7.033E+00 | 2.102E+01 | 4.074E+01 | 2.308E+01 | 1.660E+01 | 4.447E+01 |
| F9 | AVG | **9.490E+02** | 9.909E+02 | 1.013E+03 | 1.180E+03 | 1.010E+03 | 1.164E+03 | 1.008E+03 | 1.120E+03 | 1.107E+03 | 1.169E+03 | 1.123E+03 |
|  | STD | 1.386E+01 | 2.911E+01 | 6.631E+01 | 6.596E+01 | 9.062E+00 | **8.489E+00** | 2.240E+01 | 4.490E+01 | 2.144E+01 | 1.927E+01 | 5.164E+01 |
| F10 | AVG | 2.204E+03 | 3.729E+03 | 3.123E+03 | 5.480E+03 | **1.029E+03** | 7.566E+03 | 3.196E+03 | 4.459E+03 | 5.267E+03 | 6.847E+03 | 4.952E+03 |
|  | STD | 3.883E+02 | 5.863E+02 | 6.002E+02 | 6.430E+02 | **3.488E+01** | 2.082E+02 | 5.424E+02 | 9.304E+02 | 5.126E+02 | 4.749E+02 | 7.206E+02 |
| F11 | AVG | **3.332E+03** | 4.116E+03 | 4.899E+03 | 5.405E+03 | 5.797E+03 | 8.054E+03 | 3.943E+03 | 5.215E+03 | 5.943E+03 | 8.004E+03 | 6.188E+03 |
|  | STD | 6.213E+02 | 6.115E+02 | 1.943E+03 | 6.105E+02 | **2.421E+02** | 2.614E+02 | 7.849E+02 | 8.623E+02 | 5.750E+02 | 3.807E+02 | 8.141E+02 |
| F12 | AVG | 1.200E+03 | **1.200E+03** | 1.202E+03 | 1.201E+03 | 1.201E+03 | 1.202E+03 | 1.202E+03 | 1.200E+03 | 1.202E+03 | 1.203E+03 | 1.202E+03 |
|  | STD | 3.099E-01 | **1.283E-01** | 3.354E-01 | 3.490E-01 | 1.343E-01 | 2.603E-01 | 1.028E+00 | 2.046E-01 | 2.949E-01 | 2.332E-01 | 5.016E-01 |
| F13 | AVG | **1.300E+03** | 1.300E+03 | 1.301E+03 | 1.300E+03 | 1.300E+03 | 1.303E+03 | 1.300E+03 | 1.302E+03 | 1.300E+03 | 1.303E+03 | 1.301E+03 |
|  | STD | 5.009E-02 | 9.263E-02 | 1.066E-01 | 1.132E-01 | **3.600E-02** | 2.767E-01 | 3.638E-01 | 1.251E+00 | 7.576E-02 | 2.470E-01 | 1.285E-01 |
| F14 | AVG | 1.400E+03 | 1.400E+03 | 1.401E+03 | 1.400E+03 | 1.400E+03 | 1.442E+03 | 1.407E+03 | 1.429E+03 | 1.400E+03 | 1.442E+03 | **1.400E+03** |
|  | STD | 1.976E-01 | 2.790E-01 | 2.686E-01 | 1.208E-01 | 8.449E-02 | 4.534E+00 | 9.791E+00 | 2.360E+01 | 1.258E-01 | 8.197E+00 | **6.504E-02** |
| F15 | AVG | **1.505E+03** | 1.507E+03 | 2.044E+03 | 1.527E+03 | 1.512E+03 | 1.427E+04 | 1.564E+03 | 1.531E+05 | 1.517E+03 | 4.904E+03 | 1.570E+03 |
|  | STD | 1.828E+00 | 2.141E+00 | 2.698E+03 | 4.549E+00 | **6.752E-01** | 6.146E+03 | 8.776E+01 | 4.515E+05 | 1.107E+00 | 3.099E+03 | 2.058E+01 |
| F16 | AVG | **1.610E+03** | 1.612E+03 | 1.612E+03 | 1.613E+03 | 1.611E+03 | 1.613E+03 | 1.611E+03 | 1.613E+03 | 1.612E+03 | 1.613E+03 | 1.612E+03 |
|  | STD | 6.121E-01 | 6.184E-01 | 3.473E-01 | 3.381E-01 | 2.506E-01 | **1.987E-01** | 7.232E-01 | 5.274E-01 | 4.023E-01 | 2.943E-01 | 4.975E-01 |
| F17 | AVG | **5.866E+04** | 1.717E+05 | 2.764E+05 | 1.073E+05 | 1.842E+06 | 6.420E+06 | 1.918E+06 | 2.437E+06 | 2.663E+05 | 6.275E+06 | 4.261E+06 |
|  | STD | **4.015E+04** | 1.402E+05 | 5.557E+05 | 6.954E+04 | 8.316E+05 | 2.011E+06 | 1.712E+06 | 3.570E+06 | 1.236E+05 | 3.110E+06 | 2.761E+06 |
| F18 | AVG | 8.742E+03 | 1.030E+04 | 9.845E+03 | 9.316E+04 | **8.386E+03** | 3.010E+08 | 5.408E+06 | 8.658E+06 | 2.060E+06 | 1.660E+08 | 1.407E+04 |
|  | STD | 8.137E+03 | 7.916E+03 | 8.716E+03 | 5.079E+04 | **4.200E+03** | 7.839E+07 | 1.536E+07 | 4.715E+07 | 7.189E+05 | 9.343E+07 | 3.793E+04 |
| F19 | AVG | 1.909E+03 | 1.910E+03 | 1.919E+03 | 1.934E+03 | **1.908E+03** | 2.005E+03 | 1.939E+03 | 1.978E+03 | 1.917E+03 | 1.990E+03 | 1.940E+03 |
|  | STD | 3.150E+00 | 2.154E+00 | 2.300E+01 | 3.609E+01 | **7.180E-01** | 1.297E+01 | 2.887E+01 | 6.299E+01 | 2.685E+00 | 2.497E+01 | 3.275E+01 |
| F20 | AVG | **2.201E+03** | 2.280E+03 | 5.014E+03 | 2.376E+03 | 5.347E+03 | 2.070E+04 | 1.578E+04 | 7.444E+04 | 2.338E+03 | 1.593E+04 | 2.360E+04 |
|  | STD | **6.664E+01** | 9.164E+01 | 7.787E+03 | 1.081E+02 | 1.797E+03 | 6.367E+03 | 9.656E+03 | 5.404E+04 | 7.450E+01 | 4.531E+03 | 1.533E+04 |
| F21 | AVG | **5.506E+04** | 6.351E+04 | 9.988E+04 | 6.117E+04 | 2.992E+05 | 1.894E+06 | 1.047E+06 | 8.683E+05 | 1.115E+05 | 1.257E+06 | 1.393E+06 |
|  | STD | 4.545E+04 | 3.136E+04 | 1.217E+05 | **3.127E+04** | 2.029E+05 | 5.348E+05 | 2.625E+06 | 1.372E+06 | 6.178E+04 | 8.266E+05 | 1.257E+06 |
| F22 | AVG | 2.508E+03 | 2.642E+03 | 2.648E+03 | 3.286E+03 | **2.361E+03** | 3.008E+03 | 2.595E+03 | 2.961E+03 | 2.890E+03 | 2.937E+03 | 2.985E+03 |
|  | STD | 1.460E+02 | 1.747E+02 | 2.404E+02 | 3.027E+02 | **7.618E+01** | 1.095E+02 | 1.472E+02 | 2.238E+02 | 2.032E+02 | 1.298E+02 | 2.270E+02 |
| F23 | AVG | **2.500E+03** | 2.616E+03 | 2.617E+03 | 2.615E+03 | 2.615E+03 | 2.729E+03 | 2.640E+03 | 2.696E+03 | 2.616E+03 | 2.668E+03 | 2.625E+03 |
|  | STD | **0.000E+00** | 5.266E-01 | 4.098E+00 | 3.549E-03 | 1.388E-12 | 1.618E+01 | 1.344E+01 | 7.120E+01 | 6.791E-01 | 1.386E+01 | 3.463E+01 |
| F24 | AVG | **2.600E+03** | 2.626E+03 | 2.644E+03 | 2.659E+03 | 2.626E+03 | 2.705E+03 | 2.600E+03 | 2.678E+03 | 2.627E+03 | 2.600E+03 | 2.609E+03 |
|  | STD | **0.000E+00** | 1.260E+01 | 1.007E+01 | 2.475E+01 | 2.091E+00 | 3.610E+00 | 4.498E-04 | 3.086E+01 | 6.774E+00 | 4.442E-02 | 2.369E+01 |
| F25 | AVG | **2.700E+03** | 2.706E+03 | 2.708E+03 | 2.733E+03 | 2.708E+03 | 2.732E+03 | 2.711E+03 | 2.714E+03 | 2.715E+03 | 2.726E+03 | 2.712E+03 |
|  | STD | **0.000E+00** | 1.987E+00 | 4.099E+00 | 1.152E+01 | 1.212E+00 | 3.760E+00 | 4.143E+00 | 5.906E+00 | 6.795E+00 | 7.318E+00 | 1.757E+01 |
| F26 | AVG | **2.700E+03** | 2.746E+03 | 2.734E+03 | 2.711E+03 | 2.700E+03 | 2.702E+03 | 2.737E+03 | 2.711E+03 | 2.769E+03 | 2.702E+03 | 2.704E+03 |
|  | STD | 6.349E-02 | 7.256E+01 | 8.038E+01 | 5.524E+01 | **3.841E-02** | 3.158E-01 | 4.876E+01 | 4.491E+01 | 6.138E+01 | 7.232E-01 | 1.817E+01 |
| F27 | AVG | **2.900E+03** | 3.256E+03 | 3.414E+03 | 3.784E+03 | 3.190E+03 | 3.805E+03 | 3.337E+03 | 3.636E+03 | 3.364E+03 | 3.482E+03 | 3.804E+03 |
|  | STD | **0.000E+00** | 1.259E+02 | 1.006E+02 | 4.683E+02 | 7.632E+01 | 2.234E+01 | 1.300E+02 | 2.268E+02 | 2.853E+02 | 3.286E+02 | 3.712E+02 |
| F28 | AVG | **3.000E+03** | 3.795E+03 | 3.823E+03 | 5.341E+03 | 3.629E+03 | 4.274E+03 | 4.008E+03 | 3.918E+03 | 7.219E+03 | 4.854E+03 | 4.969E+03 |
|  | STD | **0.000E+00** | 1.643E+02 | 1.694E+02 | 7.132E+02 | 1.979E+01 | 1.760E+02 | 2.826E+02 | 2.007E+02 | 9.513E+02 | 3.128E+02 | 7.913E+02 |
| F29 | AVG | **3.123E+03** | 1.358E+06 | 3.959E+06 | 3.923E+07 | 8.695E+03 | 3.224E+06 | 1.256E+06 | 2.712E+06 | 4.027E+04 | 1.239E+07 | 5.895E+06 |
|  | STD | **2.468E+01** | 3.482E+06 | 4.739E+06 | 3.901E+07 | 1.232E+04 | 1.453E+06 | 2.106E+06 | 3.773E+06 | 6.565E+04 | 7.043E+06 | 4.563E+06 |
| F30 | AVG | **3.895E+03** | 8.804E+03 | 9.892E+03 | 1.059E+04 | 5.969E+03 | 1.796E+05 | 4.303E+04 | 4.434E+04 | 1.464E+04 | 2.655E+05 | 7.404E+04 |
|  | STD | **2.360E+02** | 2.095E+03 | 6.700E+03 | 2.690E+03 | 1.041E+03 | 5.266E+04 | 2.790E+04 | 3.889E+04 | 5.271E+03 | 1.001E+05 | 5.795E+04 |
|  | +/-/= | **~** | 22/1/7 | 25/0/5 | 27/0/3 | 21/5/4 | 30/0/0 | 29/0/1 | 29/0/1 | 29/0/1 | 30/0/0 | 28/0/2 |
|  | Mean | **1.433** | 3.467 | 5.467 | 6.533 | 3.267 | 9.833 | 5.900 | 7.867 | 5.933 | 9.033 | 7.267 |
|  | Rank | **1** | 3 | 4 | 7 | 2 | 11 | 5 | 9 | 6 | 10 | 8 |

**Table A. 4.** The comparison results of MGACO with other advanced methods

| Fun | Item | MGACO | CDLOBA | BMWOA | RCBA | HGWO | EWOA | OBLGWO | m_SCA | OBSCA | ACWOA | RCACO |
| --- | --- | --- | --- | --- | --- | --- | --- | --- | --- | --- | --- | --- |
| F1 | AVG | 5.260E+05 | 4.947E+05 | 1.147E+08 | 1.283E+06 | 1.743E+08 | 3.969E+06 | **1.960E+07** | 5.857E+07 | 4.111E+08 | 1.305E+08 | 1.496E+06 |
|  | STD | 3.930E+05 | 3.092E+05 | 3.923E+07 | 6.942E+05 | 3.506E+07 | 2.999E+06 | **1.177E+07** | 2.991E+07 | 1.064E+08 | 5.767E+07 | 2.739E+06 |
| F2 | AVG | **1.183E+04** | 1.454E+04 | 2.106E+08 | 2.347E+04 | 8.682E+09 | 1.317E+04 | 1.639E+07 | 6.227E+09 | 2.520E+10 | 6.257E+09 | 1.293E+04 |
|  | STD | 1.159E+04 | 1.245E+04 | 1.099E+08 | **8.182E+03** | 1.631E+09 | 1.185E+04 | 1.128E+07 | 3.490E+09 | 4.812E+09 | 2.632E+09 | 1.272E+04 |
| F3 | AVG | 3.595E+02 | 7.550E+04 | 5.556E+04 | **3.313E+02** | 6.728E+04 | 4.773E+03 | 8.268E+03 | 2.484E+04 | 5.129E+04 | 4.730E+04 | 3.229E+03 |
|  | STD | 5.500E+01 | 2.490E+04 | 7.506E+03 | **1.331E+01** | 4.639E+03 | 3.600E+03 | 2.606E+03 | 6.189E+03 | 8.277E+03 | 9.753E+03 | 3.558E+03 |
| F4 | AVG | **4.099E+02** | 4.748E+02 | 6.913E+02 | 4.712E+02 | 9.191E+02 | 5.187E+02 | 5.248E+02 | 7.956E+02 | 2.363E+03 | 1.215E+03 | 4.298E+02 |
|  | STD | **2.043E+01** | 4.169E+01 | 7.492E+01 | 4.204E+01 | 5.778E+01 | 3.284E+01 | 3.799E+01 | 1.565E+02 | 5.827E+02 | 3.769E+02 | 3.298E+01 |
| F5 | AVG | 5.201E+02 | 5.209E+02 | 5.210E+02 | **5.201E+02** | 5.208E+02 | 5.201E+02 | 5.210E+02 | 5.206E+02 | 5.210E+02 | 5.208E+02 | 5.207E+02 |
|  | STD | 8.645E-02 | 1.899E-01 | 6.497E-02 | 9.532E-02 | 8.439E-02 | 1.099E-01 | **5.314E-02** | 1.199E-01 | 5.347E-02 | 1.335E-01 | 7.192E-02 |
| F6 | AVG | 6.084E+02 | 6.356E+02 | 6.326E+02 | 6.394E+02 | 6.262E+02 | 6.224E+02 | 6.189E+02 | 6.222E+02 | 6.323E+02 | 6.352E+02 | **6.083E+02** |
|  | STD | 2.666E+00 | 2.874E+00 | 3.006E+00 | 2.969E+00 | 2.367E+00 | 3.877E+00 | 3.145E+00 | 3.084E+00 | **1.281E+00** | 2.755E+00 | 2.742E+00 |
| F7 | AVG | 7.000E+02 | 7.000E+02 | 7.030E+02 | 7.001E+02 | 7.426E+02 | 7.000E+02 | 7.012E+02 | 7.436E+02 | 8.965E+02 | 7.423E+02 | **7.000E+02** |
|  | STD | 9.291E-03 | 9.936E-03 | 8.902E-01 | 2.459E-02 | 7.488E+00 | 4.506E-02 | 9.877E-02 | 2.452E+01 | 3.987E+01 | 2.300E+01 | **8.865E-03** |
| F8 | AVG | 8.354E+02 | 1.047E+03 | 9.666E+02 | 1.010E+03 | 1.007E+03 | 8.318E+02 | 9.303E+02 | 9.312E+02 | 1.066E+03 | 9.897E+02 | **8.270E+02** |
|  | STD | 9.534E+00 | 3.677E+01 | 2.193E+01 | 5.087E+01 | 1.202E+01 | 8.435E+00 | 4.038E+01 | 2.928E+01 | 1.817E+01 | 2.471E+01 | **7.494E+00** |
| F9 | AVG | **9.485E+02** | 1.234E+03 | 1.124E+03 | 1.173E+03 | 1.137E+03 | 1.069E+03 | 1.067E+03 | 1.046E+03 | 1.204E+03 | 1.127E+03 | 1.008E+03 |
|  | STD | 1.367E+01 | 5.629E+01 | 3.114E+01 | 5.405E+01 | **1.210E+01** | 4.482E+01 | 2.940E+01 | 2.418E+01 | 1.491E+01 | 1.735E+01 | 2.387E+01 |
| F10 | AVG | 2.033E+03 | 5.613E+03 | 4.892E+03 | 5.638E+03 | 5.693E+03 | **1.639E+03** | 3.945E+03 | 4.092E+03 | 6.214E+03 | 4.860E+03 | 1.872E+03 |
|  | STD | 3.459E+02 | 6.972E+02 | 6.429E+02 | 7.169E+02 | **2.452E+02** | 3.283E+02 | 8.244E+02 | 6.497E+02 | 3.603E+02 | 8.994E+02 | 3.310E+02 |
| F11 | AVG | **3.139E+03** | 5.545E+03 | 7.216E+03 | 5.918E+03 | 6.613E+03 | 4.670E+03 | 5.221E+03 | 4.648E+03 | 7.276E+03 | 6.214E+03 | 5.753E+03 |
|  | STD | 6.681E+02 | 6.650E+02 | 7.411E+02 | 8.426E+02 | 4.413E+02 | 6.642E+02 | 7.706E+02 | 8.916E+02 | **3.913E+02** | 1.028E+03 | 4.957E+02 |
| F12 | AVG | 1.200E+03 | 1.200E+03 | 1.203E+03 | 1.201E+03 | 1.201E+03 | 1.200E+03 | **1.202E+03** | 1.201E+03 | 1.202E+03 | 1.202E+03 | 1.201E+03 |
|  | STD | 3.184E-01 | 1.962E-01 | 5.671E-01 | 2.704E-01 | 2.177E-01 | **1.893E-01** | 7.075E-01 | 2.316E-01 | 3.513E-01 | 6.191E-01 | 2.609E-01 |
| F13 | AVG | **1.300E+03** | 1.300E+03 | 1.301E+03 | 1.300E+03 | 1.302E+03 | 1.300E+03 | 1.301E+03 | 1.301E+03 | 1.304E+03 | 1.302E+03 | 1.300E+03 |
|  | STD | **6.397E-02** | 1.277E-01 | 1.490E-01 | 9.947E-02 | 4.040E-01 | 1.224E-01 | 1.175E-01 | 6.444E-01 | 4.004E-01 | 9.197E-01 | 7.399E-02 |
| F14 | AVG | 1.400E+03 | 1.400E+03 | 1.400E+03 | 1.400E+03 | 1.422E+03 | **1.400E+03** | 1.400E+03 | 1.417E+03 | 1.470E+03 | 1.418E+03 | 1.400E+03 |
|  | STD | 1.355E-01 | 1.330E-01 | **5.098E-02** | 1.059E-01 | 5.037E+00 | 5.393E-02 | 2.496E-01 | 9.272E+00 | 1.175E+01 | 1.204E+01 | 2.095E-01 |
| F15 | AVG | **1.506E+03** | 1.704E+03 | 1.579E+03 | 1.536E+03 | 1.854E+03 | 1.519E+03 | 1.516E+03 | 2.439E+03 | 1.600E+04 | 1.872E+03 | 1.514E+03 |
|  | STD | **1.505E+00** | 6.862E+01 | 3.088E+01 | 9.004E+00 | 2.052E+02 | 7.391E+00 | 5.561E+00 | 1.321E+03 | 1.042E+04 | 4.512E+02 | 4.518E+00 |
| F16 | AVG | **1.611E+03** | 1.613E+03 | 1.613E+03 | 1.613E+03 | 1.613E+03 | 1.612E+03 | 1.612E+03 | 1.612E+03 | 1.613E+03 | 1.612E+03 | 1.611E+03 |
|  | STD | 5.787E-01 | 3.327E-01 | 2.172E-01 | 4.789E-01 | 2.337E-01 | 6.185E-01 | 3.113E-01 | 4.121E-01 | **1.453E-01** | 4.491E-01 | 5.544E-01 |
| F17 | AVG | 6.830E+04 | 4.161E+04 | 6.067E+06 | 1.423E+05 | 5.922E+06 | 9.725E+05 | **1.332E+06** | 1.493E+06 | 9.353E+06 | 1.538E+07 | 1.544E+05 |
|  | STD | 4.000E+04 | 3.165E+04 | 4.324E+06 | 7.741E+04 | 2.888E+06 | 7.286E+05 | **7.610E+05** | 1.160E+06 | 4.559E+06 | 1.029E+07 | 1.157E+05 |
| F18 | AVG | 7.207E+03 | 9.339E+03 | 1.386E+05 | **6.604E+03** | 1.329E+08 | 7.624E+03 | 3.003E+04 | 1.571E+07 | 1.778E+08 | 4.142E+07 | 7.558E+03 |
|  | STD | 6.567E+03 | 7.543E+03 | 1.480E+05 | 5.939E+03 | 3.193E+07 | 5.528E+03 | 2.841E+04 | 2.237E+07 | 1.242E+08 | 3.980E+07 | **5.353E+03** |
| F19 | AVG | **1.911E+03** | 1.983E+03 | 1.945E+03 | 1.922E+03 | 1.992E+03 | 1.914E+03 | 1.922E+03 | 1.946E+03 | 2.010E+03 | 2.011E+03 | 1.913E+03 |
|  | STD | 1.204E+01 | 4.791E+01 | 4.422E+01 | 1.852E+01 | **9.745E+00** | 1.243E+01 | 2.424E+01 | 2.826E+01 | 1.766E+01 | 3.620E+01 | 2.105E+01 |
| F20 | AVG | **2.200E+03** | 2.634E+04 | 4.308E+04 | 2.420E+03 | 5.945E+04 | 4.515E+03 | 6.109E+03 | 1.360E+04 | 3.031E+04 | 3.736E+04 | 2.585E+03 |
|  | STD | **7.954E+01** | 1.319E+04 | 2.487E+04 | 1.393E+02 | 2.799E+04 | 3.237E+03 | 2.598E+03 | 5.242E+03 | 1.061E+04 | 1.764E+04 | 4.435E+02 |
| F21 | AVG | 4.322E+04 | 3.739E+04 | 1.459E+06 | 6.113E+04 | 2.297E+06 | 5.035E+05 | **6.275E+05** | 6.710E+05 | 2.328E+06 | 4.989E+06 | 1.398E+05 |
|  | STD | 3.188E+04 | 2.122E+04 | 1.229E+06 | 3.613E+04 | 1.750E+06 | 3.197E+05 | **3.992E+05** | 1.211E+06 | 1.194E+06 | 4.383E+06 | 1.375E+05 |
| F22 | AVG | **2.456E+03** | 3.261E+03 | 2.901E+03 | 3.313E+03 | 3.027E+03 | 2.739E+03 | 2.751E+03 | 2.530E+03 | 3.142E+03 | 3.085E+03 | 2.459E+03 |
|  | STD | 1.660E+02 | 3.108E+02 | 2.304E+02 | 3.536E+02 | 1.382E+02 | 1.946E+02 | 1.623E+02 | 2.117E+02 | **1.365E+02** | 2.644E+02 | 1.563E+02 |
| F23 | AVG | **2.500E+03** | 2.616E+03 | 2.501E+03 | 2.615E+03 | 2.517E+03 | 2.615E+03 | 2.618E+03 | 2.637E+03 | 2.689E+03 | 2.527E+03 | 2.500E+03 |
|  | STD | **0.000E+00** | 1.658E+00 | 4.820E-01 | 7.366E-03 | 5.284E+01 | 2.732E-01 | 1.250E+00 | 6.503E+00 | 1.618E+01 | 6.902E+01 | 1.323E-02 |
| F24 | AVG | **2.600E+03** | 2.713E+03 | 2.600E+03 | 2.675E+03 | 2.600E+03 | 2.604E+03 | 2.600E+03 | 2.600E+03 | 2.600E+03 | 2.600E+03 | 2.600E+03 |
|  | STD | **0.000E+00** | 4.512E+01 | 1.907E-01 | 3.034E+01 | 0.000E+00 | 8.943E+00 | 0.000E+00 | 6.715E-04 | 4.200E-04 | 3.294E-06 | 3.607E-02 |
| F25 | AVG | **2.700E+03** | 2.721E+03 | 2.700E+03 | 2.729E+03 | 2.700E+03 | 2.715E+03 | 2.700E+03 | 2.714E+03 | 2.700E+03 | 2.700E+03 | 2.700E+03 |
|  | STD | **0.000E+00** | 1.312E+01 | 1.287E-02 | 1.157E+01 | 0.000E+00 | 1.008E+01 | 0.000E+00 | 2.688E+00 | 2.334E-08 | 0.000E+00 | 2.855E-04 |
| F26 | AVG | **2.700E+03** | 2.714E+03 | 2.701E+03 | 2.727E+03 | 2.746E+03 | 2.710E+03 | 2.701E+03 | 2.701E+03 | 2.704E+03 | 2.757E+03 | 2.714E+03 |
|  | STD | **6.607E-02** | 5.452E+01 | 1.426E-01 | 7.911E+01 | 4.762E+01 | 3.041E+01 | 1.165E-01 | 3.555E-01 | 4.510E-01 | 4.997E+01 | 3.446E+01 |
| F27 | AVG | **2.900E+03** | 3.846E+03 | 2.900E+03 | 4.050E+03 | 3.603E+03 | 3.621E+03 | 3.045E+03 | 3.176E+03 | 3.252E+03 | 3.679E+03 | 2.900E+03 |
|  | STD | **0.000E+00** | 4.215E+02 | 2.324E-01 | 3.878E+02 | 1.986E+02 | 2.209E+02 | 2.804E+02 | 1.295E+02 | 3.798E+01 | 3.602E+02 | 3.082E-03 |
| F28 | AVG | 3.008E+03 | 5.574E+03 | 3.000E+03 | 5.870E+03 | 4.279E+03 | 4.233E+03 | 3.587E+03 | 3.901E+03 | 5.483E+03 | 3.871E+03 | **3.000E+03** |
|  | STD | 4.164E+01 | 6.763E+02 | 2.759E-01 | 1.075E+03 | 2.897E+02 | 4.300E+02 | 5.955E+02 | 2.308E+02 | 2.858E+02 | 1.198E+03 | **8.178E-03** |
| F29 | AVG | **3.114E+03** | 1.981E+07 | 1.431E+06 | 1.162E+07 | 4.360E+06 | 3.756E+06 | 3.474E+06 | 8.782E+05 | 2.065E+07 | 1.981E+07 | 5.977E+05 |
|  | STD | **2.333E+00** | 1.770E+07 | 3.088E+06 | 1.551E+07 | 3.215E+06 | 4.366E+06 | 4.299E+06 | 1.295E+06 | 1.133E+07 | 1.832E+07 | 2.258E+06 |
| F30 | AVG | 4.002E+03 | 6.771E+04 | 5.478E+04 | 1.033E+04 | **3.200E+03** | 9.888E+03 | 1.997E+04 | 6.086E+04 | 4.151E+05 | 3.160E+05 | 7.836E+03 |
|  | STD | 3.270E+02 | 1.022E+05 | 4.237E+04 | 3.106E+03 | **1.005E-04** | 3.284E+03 | 8.579E+03 | 3.358E+04 | 1.926E+05 | 1.919E+05 | 5.531E+03 |
|  | +/-/= | **~** | 22/1/7 | 28/1/1 | 26/2/2 | 27/1/2 | 24/1/5 | 28/0/2 | 29/0/1 | 29/0/1 | 28/0/2 | 20/2/8 |
|  | Mean | **1.633** | 6.967 | 6.767 | 6.233 | 7.700 | 4.567 | 5.033 | 6.133 | 9.467 | 8.000 | 3.200 |
|  | Rank | **1** | 8 | 7 | 6 | 9 | 3 | 4 | 5 | 11 | 10 | 2 |

**Table A. 5.** The PSNR comparison results of all methods

| Image | Level | Item | MGACO-MIS | ACOR-MIS | MVO-MIS | HHO-MIS | SCA-MIS | BLPSO-MIS | IGWO-MIS | IWOA-MIS | CLPSO-MIS |
| --- | --- | --- | --- | --- | --- | --- | --- | --- | --- | --- | --- |
| A | 4 | AVG | 1.939E+01 | 1.928E+01 | **1.940E+01** | 1.695E+01 | 1.628E+01 | 1.746E+01 | 1.899E+01 | 1.705E+01 | 1.759E+01 |
|  |  | STD | 1.131E+00 | **1.120E+00** | 1.208E+00 | 1.846E+00 | 1.854E+00 | 1.253E+00 | 1.316E+00 | 1.761E+00 | 1.355E+00 |
|  | 5 | AVG | 2.042E+01 | 2.021E+01 | **2.053E+01** | 1.863E+01 | 1.727E+01 | 1.899E+01 | 2.013E+01 | 1.820E+01 | 1.905E+01 |
|  |  | STD | 1.318E+00 | 1.255E+00 | **1.157E+00** | 2.197E+00 | 2.309E+00 | 1.304E+00 | 1.157E+00 | 2.174E+00 | 1.459E+00 |
|  | 6 | AVG | **2.156E+01** | 2.141E+01 | 2.118E+01 | 1.968E+01 | 1.832E+01 | 1.992E+01 | 2.019E+01 | 1.955E+01 | 1.949E+01 |
|  |  | STD | 1.067E+00 | **9.963E-01** | 1.372E+00 | 1.829E+00 | 2.626E+00 | 1.021E+00 | 1.424E+00 | 1.758E+00 | 1.815E+00 |
|  | 15 | AVG | 2.706E+01 | **2.710E+01** | 2.649E+01 | 2.522E+01 | 2.338E+01 | 2.557E+01 | 2.468E+01 | 2.550E+01 | 2.529E+01 |
|  |  | STD | 1.250E+00 | **1.101E+00** | 1.872E+00 | 1.988E+00 | 1.938E+00 | 1.833E+00 | 2.071E+00 | 1.545E+00 | 1.747E+00 |
|  | 20 | AVG | **2.873E+01** | 2.854E+01 | 2.767E+01 | 2.743E+01 | 2.555E+01 | 2.764E+01 | 2.680E+01 | 2.771E+01 | 2.706E+01 |
|  |  | STD | **1.186E+00** | 1.306E+00 | 1.624E+00 | 2.386E+00 | 2.412E+00 | 1.310E+00 | 1.976E+00 | 1.646E+00 | 1.657E+00 |
|  | 25 | AVG | 2.984E+01 | **3.026E+01** | 2.886E+01 | 2.906E+01 | 2.783E+01 | 2.904E+01 | 2.882E+01 | 2.910E+01 | 2.888E+01 |
|  |  | STD | **9.663E-01** | 1.268E+00 | 1.667E+00 | 2.112E+00 | 2.193E+00 | 1.343E+00 | 1.242E+00 | 1.673E+00 | 1.816E+00 |
| B | 4 | AVG | 1.707E+01 | 1.736E+01 | 1.715E+01 | **1.748E+01** | 1.397E+01 | 1.666E+01 | 1.638E+01 | 1.678E+01 | 1.623E+01 |
|  |  | STD | **9.308E-01** | 1.306E+00 | 1.119E+00 | 1.430E+00 | 1.691E+00 | 1.383E+00 | 1.218E+00 | 1.597E+00 | 1.904E+00 |
|  | 5 | AVG | **1.960E+01** | 1.952E+01 | 1.922E+01 | 1.796E+01 | 1.525E+01 | 1.808E+01 | 1.825E+01 | 1.825E+01 | 1.785E+01 |
|  |  | STD | **6.953E-01** | 9.366E-01 | 1.486E+00 | 1.724E+00 | 2.564E+00 | 1.420E+00 | 1.563E+00 | 1.457E+00 | 1.698E+00 |
|  | 6 | AVG | 2.094E+01 | **2.100E+01** | 2.006E+01 | 1.940E+01 | 1.639E+01 | 1.922E+01 | 1.919E+01 | 1.908E+01 | 1.898E+01 |
|  |  | STD | **9.069E-01** | 9.610E-01 | 1.963E+00 | 2.043E+00 | 2.218E+00 | 1.340E+00 | 1.829E+00 | 2.181E+00 | 1.836E+00 |
|  | 15 | AVG | **2.729E+01** | 2.728E+01 | 2.599E+01 | 2.502E+01 | 2.248E+01 | 2.490E+01 | 2.476E+01 | 2.452E+01 | 2.566E+01 |
|  |  | STD | **1.007E+00** | 1.199E+00 | 2.381E+00 | 1.855E+00 | 2.318E+00 | 1.490E+00 | 1.566E+00 | 1.960E+00 | 1.538E+00 |
|  | 20 | AVG | **2.920E+01** | 2.882E+01 | 2.803E+01 | 2.686E+01 | 2.540E+01 | 2.707E+01 | 2.650E+01 | 2.726E+01 | 2.716E+01 |
|  |  | STD | **1.105E+00** | 1.178E+00 | 1.439E+00 | 2.134E+00 | 2.038E+00 | 1.248E+00 | 1.403E+00 | 1.595E+00 | 1.341E+00 |
|  | 25 | AVG | **2.997E+01** | 2.945E+01 | 2.939E+01 | 2.856E+01 | 2.724E+01 | 2.842E+01 | 2.853E+01 | 2.853E+01 | 2.845E+01 |
|  |  | STD | 1.692E+00 | 1.982E+00 | 1.745E+00 | 1.817E+00 | 1.931E+00 | **1.402E+00** | 1.985E+00 | 1.601E+00 | 1.528E+00 |
| C | 4 | AVG | 1.930E+01 | 1.920E+01 | 1.877E+01 | 1.838E+01 | 1.824E+01 | 1.815E+01 | **1.938E+01** | 1.835E+01 | 1.823E+01 |
|  |  | STD | 6.977E-01 | **5.785E-01** | 1.834E+00 | 1.606E+00 | 2.763E+00 | 1.080E+00 | 1.179E+00 | 1.561E+00 | 1.168E+00 |
|  | 5 | AVG | **2.067E+01** | 2.042E+01 | 2.027E+01 | 1.900E+01 | 1.864E+01 | 1.953E+01 | 2.054E+01 | 1.873E+01 | 1.901E+01 |
|  |  | STD | **5.192E-01** | 6.877E-01 | 1.715E+00 | 2.153E+00 | 2.078E+00 | 1.163E+00 | 1.470E+00 | 1.731E+00 | 1.490E+00 |
|  | 6 | AVG | **2.191E+01** | 2.138E+01 | 2.115E+01 | 2.009E+01 | 1.974E+01 | 2.053E+01 | 2.114E+01 | 1.986E+01 | 2.056E+01 |
|  |  | STD | 1.042E+00 | 1.140E+00 | 1.546E+00 | 1.676E+00 | 1.983E+00 | 1.216E+00 | 1.536E+00 | 1.570E+00 | **1.038E+00** |
|  | 15 | AVG | **2.774E+01** | 2.710E+01 | 2.645E+01 | 2.677E+01 | 2.423E+01 | 2.634E+01 | 2.609E+01 | 2.560E+01 | 2.544E+01 |
|  |  | STD | **1.241E+00** | 1.793E+00 | 1.475E+00 | 1.769E+00 | 2.137E+00 | 1.295E+00 | 2.358E+00 | 2.324E+00 | 1.425E+00 |
|  | 20 | AVG | **2.929E+01** | 2.898E+01 | 2.860E+01 | 2.779E+01 | 2.614E+01 | 2.824E+01 | 2.781E+01 | 2.701E+01 | 2.829E+01 |
|  |  | STD | **9.464E-01** | 1.100E+00 | 2.137E+00 | 2.809E+00 | 2.474E+00 | 1.441E+00 | 1.501E+00 | 2.789E+00 | 1.618E+00 |
|  | 25 | AVG | **3.031E+01** | 2.982E+01 | 2.978E+01 | 2.975E+01 | 2.704E+01 | 2.945E+01 | 2.928E+01 | 2.900E+01 | 2.876E+01 |
|  |  | STD | 1.812E+00 | 1.604E+00 | 1.840E+00 | 2.469E+00 | 2.449E+00 | 1.723E+00 | **1.478E+00** | 2.077E+00 | 2.019E+00 |
| D | 4 | AVG | 1.890E+01 | **1.913E+01** | 1.787E+01 | 1.556E+01 | 1.599E+01 | 1.688E+01 | 1.606E+01 | 1.652E+01 | 1.665E+01 |
|  |  | STD | **1.467E+00** | 1.509E+00 | 1.688E+00 | 2.904E+00 | 2.990E+00 | 1.928E+00 | 2.821E+00 | 2.150E+00 | 1.985E+00 |
|  | 5 | AVG | **2.041E+01** | 2.031E+01 | 1.971E+01 | 1.758E+01 | 1.667E+01 | 1.853E+01 | 1.783E+01 | 1.805E+01 | 1.821E+01 |
|  |  | STD | **1.092E+00** | 1.330E+00 | 1.744E+00 | 2.329E+00 | 2.196E+00 | 1.735E+00 | 2.678E+00 | 1.861E+00 | 2.123E+00 |
|  | 6 | AVG | **2.158E+01** | 2.105E+01 | 2.028E+01 | 1.918E+01 | 1.761E+01 | 1.902E+01 | 1.954E+01 | 1.915E+01 | 1.891E+01 |
|  |  | STD | **9.249E-01** | 1.027E+00 | 1.809E+00 | 2.183E+00 | 1.878E+00 | 1.610E+00 | 2.228E+00 | 2.045E+00 | 2.443E+00 |
|  | 15 | AVG | **2.738E+01** | 2.664E+01 | 2.679E+01 | 2.561E+01 | 2.387E+01 | 2.547E+01 | 2.464E+01 | 2.567E+01 | 2.582E+01 |
|  |  | STD | **1.392E+00** | 1.730E+00 | 1.774E+00 | 1.985E+00 | 2.280E+00 | 1.723E+00 | 1.615E+00 | 1.569E+00 | 1.487E+00 |
|  | 20 | AVG | 2.833E+01 | **2.880E+01** | 2.773E+01 | 2.733E+01 | 2.606E+01 | 2.727E+01 | 2.733E+01 | 2.771E+01 | 2.734E+01 |
|  |  | STD | 1.386E+00 | 1.627E+00 | 2.665E+00 | 2.686E+00 | 2.518E+00 | **1.244E+00** | 1.838E+00 | 1.729E+00 | 1.798E+00 |
|  | 25 | AVG | 2.977E+01 | **2.984E+01** | 2.948E+01 | 2.983E+01 | 2.800E+01 | 2.956E+01 | 2.892E+01 | 2.939E+01 | 2.871E+01 |
|  |  | STD | 1.546E+00 | 1.419E+00 | 1.806E+00 | 1.794E+00 | 2.210E+00 | **1.383E+00** | 1.619E+00 | 1.818E+00 | 1.978E+00 |
| E | 4 | AVG | 1.858E+01 | **1.899E+01** | 1.786E+01 | 1.689E+01 | 1.559E+01 | 1.727E+01 | 1.736E+01 | 1.720E+01 | 1.607E+01 |
|  |  | STD | **1.052E+00** | 1.085E+00 | 1.396E+00 | 1.475E+00 | 1.957E+00 | 1.891E+00 | 1.355E+00 | 1.338E+00 | 1.853E+00 |
|  | 5 | AVG | 2.066E+01 | **2.083E+01** | 1.990E+01 | 1.756E+01 | 1.639E+01 | 1.870E+01 | 1.901E+01 | 1.749E+01 | 1.850E+01 |
|  |  | STD | **7.258E-01** | 7.593E-01 | 1.226E+00 | 2.081E+00 | 2.606E+00 | 1.881E+00 | 1.203E+00 | 2.460E+00 | 1.545E+00 |
|  | 6 | AVG | **2.203E+01** | 2.182E+01 | 2.101E+01 | 1.731E+01 | 1.718E+01 | 1.910E+01 | 1.952E+01 | 1.866E+01 | 1.990E+01 |
|  |  | STD | **6.842E-01** | 9.649E-01 | 1.612E+00 | 2.757E+00 | 2.160E+00 | 1.518E+00 | 1.628E+00 | 2.234E+00 | 1.674E+00 |
|  | 15 | AVG | **2.730E+01** | 2.717E+01 | 2.613E+01 | 2.494E+01 | 2.318E+01 | 2.504E+01 | 2.482E+01 | 2.519E+01 | 2.520E+01 |
|  |  | STD | **9.773E-01** | 1.242E+00 | 2.189E+00 | 2.302E+00 | 2.458E+00 | 1.489E+00 | 1.971E+00 | 1.517E+00 | 1.762E+00 |
|  | 20 | AVG | **2.919E+01** | 2.850E+01 | 2.771E+01 | 2.672E+01 | 2.492E+01 | 2.713E+01 | 2.644E+01 | 2.775E+01 | 2.757E+01 |
|  |  | STD | **1.033E+00** | 1.813E+00 | 1.816E+00 | 2.094E+00 | 2.080E+00 | 1.822E+00 | 1.639E+00 | 1.984E+00 | 1.817E+00 |
|  | 25 | AVG | **3.016E+01** | 2.954E+01 | 2.923E+01 | 2.924E+01 | 2.732E+01 | 2.894E+01 | 2.819E+01 | 2.885E+01 | 2.882E+01 |
|  |  | STD | 1.446E+00 | **1.131E+00** | 2.011E+00 | 1.761E+00 | 1.719E+00 | 1.670E+00 | 1.622E+00 | 1.965E+00 | 1.789E+00 |
| F | 4 | AVG | **2.036E+01** | 2.031E+01 | 1.940E+01 | 1.856E+01 | 1.706E+01 | 1.824E+01 | 2.029E+01 | 1.839E+01 | 1.817E+01 |
|  |  | STD | **2.581E-01** | 4.842E-01 | 1.930E+00 | 2.202E+00 | 2.976E+00 | 1.745E+00 | 7.450E-01 | 2.276E+00 | 1.475E+00 |
|  | 5 | AVG | 2.150E+01 | **2.151E+01** | 2.144E+01 | 1.927E+01 | 1.767E+01 | 1.887E+01 | 2.037E+01 | 1.933E+01 | 1.893E+01 |
|  |  | STD | **4.987E-01** | 9.481E-01 | 9.421E-01 | 2.638E+00 | 2.166E+00 | 2.513E+00 | 1.577E+00 | 2.205E+00 | 1.402E+00 |
|  | 6 | AVG | **2.263E+01** | 2.262E+01 | 2.129E+01 | 2.034E+01 | 1.811E+01 | 2.002E+01 | 2.062E+01 | 2.076E+01 | 2.060E+01 |
|  |  | STD | 1.042E+00 | **1.012E+00** | 2.005E+00 | 1.693E+00 | 2.329E+00 | 1.539E+00 | 2.032E+00 | 1.614E+00 | 1.693E+00 |
|  | 15 | AVG | 2.695E+01 | **2.769E+01** | 2.686E+01 | 2.615E+01 | 2.356E+01 | 2.556E+01 | 2.557E+01 | 2.540E+01 | 2.588E+01 |
|  |  | STD | 1.487E+00 | 1.436E+00 | 2.075E+00 | 2.113E+00 | 2.188E+00 | 1.854E+00 | 1.700E+00 | 2.103E+00 | **1.205E+00** |
|  | 20 | AVG | 2.926E+01 | **2.926E+01** | 2.899E+01 | 2.823E+01 | 2.603E+01 | 2.778E+01 | 2.683E+01 | 2.790E+01 | 2.747E+01 |
|  |  | STD | 1.434E+00 | 1.711E+00 | 1.700E+00 | 2.463E+00 | 2.352E+00 | **1.418E+00** | 2.689E+00 | 2.247E+00 | 1.671E+00 |
|  | 25 | AVG | **3.030E+01** | 3.004E+01 | 2.956E+01 | 2.975E+01 | 2.748E+01 | 2.928E+01 | 2.837E+01 | 2.934E+01 | 2.908E+01 |
|  |  | STD | 1.467E+00 | **1.328E+00** | 1.646E+00 | 2.078E+00 | 2.494E+00 | 1.747E+00 | 2.078E+00 | 2.161E+00 | 1.606E+00 |
| G | 4 | AVG | **1.979E+01** | 1.937E+01 | 1.878E+01 | 1.708E+01 | 1.704E+01 | 1.715E+01 | 1.905E+01 | 1.774E+01 | 1.700E+01 |
|  |  | STD | 1.085E+00 | 1.453E+00 | 1.684E+00 | 2.671E+00 | 1.562E+00 | 1.645E+00 | **8.037E-01** | 1.505E+00 | 1.940E+00 |
|  | 5 | AVG | **2.122E+01** | 2.055E+01 | 1.999E+01 | 1.817E+01 | 1.770E+01 | 1.861E+01 | 2.012E+01 | 1.822E+01 | 1.843E+01 |
|  |  | STD | **7.145E-01** | 1.602E+00 | 2.018E+00 | 2.407E+00 | 1.272E+00 | 1.912E+00 | 8.561E-01 | 2.201E+00 | 1.975E+00 |
|  | 6 | AVG | **2.245E+01** | 2.207E+01 | 2.119E+01 | 1.912E+01 | 1.809E+01 | 1.962E+01 | 2.077E+01 | 1.994E+01 | 1.987E+01 |
|  |  | STD | **8.752E-01** | 1.114E+00 | 1.590E+00 | 2.276E+00 | 2.205E+00 | 1.887E+00 | 1.799E+00 | 1.971E+00 | 1.747E+00 |
|  | 15 | AVG | **2.732E+01** | 2.681E+01 | 2.628E+01 | 2.503E+01 | 2.331E+01 | 2.580E+01 | 2.510E+01 | 2.479E+01 | 2.523E+01 |
|  |  | STD | **1.225E+00** | 1.445E+00 | 1.869E+00 | 2.248E+00 | 1.825E+00 | 1.487E+00 | 2.005E+00 | 2.321E+00 | 1.596E+00 |
|  | 20 | AVG | **2.939E+01** | 2.853E+01 | 2.776E+01 | 2.850E+01 | 2.594E+01 | 2.759E+01 | 2.744E+01 | 2.760E+01 | 2.770E+01 |
|  |  | STD | 1.497E+00 | 1.544E+00 | 2.002E+00 | **1.386E+00** | 2.066E+00 | 1.425E+00 | 1.845E+00 | 2.082E+00 | 1.502E+00 |
|  | 25 | AVG | **3.007E+01** | 2.959E+01 | 2.945E+01 | 2.940E+01 | 2.742E+01 | 2.920E+01 | 2.927E+01 | 2.953E+01 | 2.890E+01 |
|  |  | STD | 1.577E+00 | 1.548E+00 | 2.556E+00 | 2.139E+00 | 2.328E+00 | **1.503E+00** | 1.529E+00 | 1.728E+00 | 1.598E+00 |
| H | 4 | AVG | **1.929E+01** | 1.905E+01 | 1.847E+01 | 1.668E+01 | 1.704E+01 | 1.630E+01 | 1.753E+01 | 1.658E+01 | 1.586E+01 |
|  |  | STD | 1.524E+00 | **1.387E+00** | 1.718E+00 | 2.596E+00 | 2.456E+00 | 2.219E+00 | 2.067E+00 | 1.813E+00 | 2.037E+00 |
|  | 5 | AVG | 2.051E+01 | **2.064E+01** | 1.950E+01 | 1.786E+01 | 1.685E+01 | 1.756E+01 | 1.939E+01 | 1.759E+01 | 1.779E+01 |
|  |  | STD | 1.470E+00 | **1.363E+00** | 1.696E+00 | 2.822E+00 | 2.435E+00 | 2.107E+00 | 1.935E+00 | 2.006E+00 | 1.978E+00 |
|  | 6 | AVG | 2.180E+01 | **2.189E+01** | 2.087E+01 | 1.884E+01 | 1.836E+01 | 1.978E+01 | 2.011E+01 | 1.880E+01 | 1.961E+01 |
|  |  | STD | **1.108E+00** | 1.205E+00 | 1.723E+00 | 2.342E+00 | 2.268E+00 | 1.814E+00 | 1.558E+00 | 2.130E+00 | 1.522E+00 |
|  | 15 | AVG | 2.755E+01 | **2.765E+01** | 2.603E+01 | 2.525E+01 | 2.384E+01 | 2.465E+01 | 2.509E+01 | 2.527E+01 | 2.583E+01 |
|  |  | STD | **1.288E+00** | 1.670E+00 | 2.041E+00 | 2.586E+00 | 2.156E+00 | 1.782E+00 | 2.316E+00 | 2.047E+00 | 1.532E+00 |
|  | 20 | AVG | 2.897E+01 | **2.919E+01** | 2.843E+01 | 2.735E+01 | 2.572E+01 | 2.748E+01 | 2.708E+01 | 2.854E+01 | 2.728E+01 |
|  |  | STD | 1.633E+00 | **1.346E+00** | 2.267E+00 | 2.986E+00 | 2.721E+00 | 1.367E+00 | 2.141E+00 | 1.913E+00 | 1.401E+00 |
|  | 25 | AVG | 3.012E+01 | **3.031E+01** | 2.968E+01 | 2.940E+01 | 2.764E+01 | 2.927E+01 | 2.852E+01 | 2.934E+01 | 2.920E+01 |
|  |  | STD | **1.549E+00** | 1.684E+00 | 1.671E+00 | 2.001E+00 | 2.583E+00 | 1.733E+00 | 2.174E+00 | 2.333E+00 | 1.675E+00 |

**Table A. 6.** The FSIM comparison results of all methods

| Image | Level | Item | MGACO-MIS | ACOR-MIS | MVO-MIS | HHO-MIS | SCA-MIS | BLPSO-MIS | IGWO-MIS | IWOA-MIS | CLPSO-MIS |
| --- | --- | --- | --- | --- | --- | --- | --- | --- | --- | --- | --- |
| A | 4 | AVG | **8.426E-01** | 8.380E-01 | 8.421E-01 | 7.681E-01 | 7.367E-01 | 7.754E-01 | 8.279E-01 | 7.630E-01 | 7.816E-01 |
|  |  | STD | 4.290E-02 | **4.188E-02** | 4.508E-02 | 5.976E-02 | 5.286E-02 | 4.195E-02 | 4.460E-02 | 6.041E-02 | 4.705E-02 |
|  | 5 | AVG | 8.730E-01 | 8.675E-01 | **8.766E-01** | 8.137E-01 | 7.658E-01 | 8.246E-01 | 8.617E-01 | 7.983E-01 | 8.288E-01 |
|  |  | STD | 4.122E-02 | 4.114E-02 | **3.691E-02** | 6.616E-02 | 6.953E-02 | 4.294E-02 | 3.695E-02 | 6.855E-02 | 4.570E-02 |
|  | 6 | AVG | **9.045E-01** | 9.013E-01 | 8.904E-01 | 8.403E-01 | 7.931E-01 | 8.504E-01 | 8.583E-01 | 8.368E-01 | 8.385E-01 |
|  |  | STD | **2.628E-02** | 2.669E-02 | 3.573E-02 | 5.089E-02 | 7.307E-02 | 3.148E-02 | 4.208E-02 | 5.372E-02 | 5.310E-02 |
|  | 15 | AVG | 9.705E-01 | **9.734E-01** | 9.578E-01 | 9.367E-01 | 9.055E-01 | 9.464E-01 | 9.270E-01 | 9.430E-01 | 9.385E-01 |
|  |  | STD | 1.553E-02 | **1.055E-02** | 2.480E-02 | 3.315E-02 | 4.377E-02 | 3.118E-02 | 4.168E-02 | 2.837E-02 | 2.926E-02 |
|  | 20 | AVG | 9.750E-01 | **9.756E-01** | 9.641E-01 | 9.554E-01 | 9.364E-01 | 9.634E-01 | 9.521E-01 | 9.635E-01 | 9.567E-01 |
|  |  | STD | **1.039E-02** | 1.169E-02 | 2.157E-02 | 3.102E-02 | 4.092E-02 | 1.590E-02 | 3.032E-02 | 2.110E-02 | 2.213E-02 |
|  | 25 | AVG | 9.810E-01 | **9.818E-01** | 9.699E-01 | 9.700E-01 | 9.602E-01 | 9.718E-01 | 9.722E-01 | 9.730E-01 | 9.700E-01 |
|  |  | STD | **5.788E-03** | 9.628E-03 | 1.808E-02 | 2.024E-02 | 2.213E-02 | 1.146E-02 | 1.087E-02 | 1.389E-02 | 1.803E-02 |
| B | 4 | AVG | **7.317E-01** | 7.292E-01 | 7.182E-01 | 7.140E-01 | 6.806E-01 | 6.901E-01 | 7.160E-01 | 7.052E-01 | 6.960E-01 |
|  |  | STD | **1.306E-02** | 1.983E-02 | 2.428E-02 | 2.859E-02 | 2.663E-02 | 2.915E-02 | 1.639E-02 | 3.033E-02 | 3.269E-02 |
|  | 5 | AVG | **7.630E-01** | 7.625E-01 | 7.479E-01 | 7.363E-01 | 7.036E-01 | 7.183E-01 | 7.327E-01 | 7.296E-01 | 7.169E-01 |
|  |  | STD | **1.886E-02** | 2.624E-02 | 2.283E-02 | 3.005E-02 | 2.827E-02 | 2.956E-02 | 2.932E-02 | 3.220E-02 | 3.373E-02 |
|  | 6 | AVG | **7.951E-01** | 7.925E-01 | 7.841E-01 | 7.535E-01 | 7.147E-01 | 7.470E-01 | 7.527E-01 | 7.565E-01 | 7.482E-01 |
|  |  | STD | **1.660E-02** | 2.399E-02 | 2.545E-02 | 4.025E-02 | 3.764E-02 | 2.694E-02 | 3.183E-02 | 4.026E-02 | 3.177E-02 |
|  | 15 | AVG | **9.072E-01** | 9.039E-01 | 8.821E-01 | 8.664E-01 | 8.220E-01 | 8.473E-01 | 8.561E-01 | 8.576E-01 | 8.747E-01 |
|  |  | STD | **1.955E-02** | 2.297E-02 | 4.335E-02 | 3.508E-02 | 4.065E-02 | 3.179E-02 | 3.215E-02 | 3.385E-02 | 2.438E-02 |
|  | 20 | AVG | **9.320E-01** | 9.271E-01 | 9.133E-01 | 8.960E-01 | 8.620E-01 | 8.858E-01 | 8.858E-01 | 9.028E-01 | 8.968E-01 |
|  |  | STD | **1.796E-02** | 1.910E-02 | 2.578E-02 | 3.638E-02 | 4.064E-02 | 2.639E-02 | 2.965E-02 | 3.148E-02 | 2.272E-02 |
|  | 25 | AVG | **9.380E-01** | 9.298E-01 | 9.288E-01 | 9.179E-01 | 8.981E-01 | 9.115E-01 | 9.128E-01 | 9.165E-01 | 9.126E-01 |
|  |  | STD | **2.260E-02** | 2.749E-02 | 2.385E-02 | 2.906E-02 | 3.123E-02 | 2.281E-02 | 3.087E-02 | 2.720E-02 | 2.717E-02 |
| C | 4 | AVG | **7.910E-01** | 7.838E-01 | 7.790E-01 | 7.483E-01 | 7.671E-01 | 7.484E-01 | 7.704E-01 | 7.637E-01 | 7.553E-01 |
|  |  | STD | **1.793E-02** | 2.366E-02 | 4.318E-02 | 3.888E-02 | 4.401E-02 | 4.269E-02 | 3.169E-02 | 4.498E-02 | 3.437E-02 |
|  | 5 | AVG | **7.995E-01** | 7.914E-01 | 7.847E-01 | 7.716E-01 | 7.582E-01 | 7.705E-01 | 7.856E-01 | 7.671E-01 | 7.786E-01 |
|  |  | STD | **1.671E-02** | 2.413E-02 | 3.566E-02 | 3.868E-02 | 4.904E-02 | 3.708E-02 | 3.737E-02 | 4.982E-02 | 3.683E-02 |
|  | 6 | AVG | **8.198E-01** | 8.109E-01 | 8.081E-01 | 7.968E-01 | 7.709E-01 | 7.888E-01 | 8.040E-01 | 7.843E-01 | 7.793E-01 |
|  |  | STD | **1.942E-02** | 2.566E-02 | 3.720E-02 | 4.043E-02 | 4.971E-02 | 3.047E-02 | 3.946E-02 | 4.463E-02 | 4.424E-02 |
|  | 15 | AVG | **9.159E-01** | 9.110E-01 | 8.945E-01 | 9.004E-01 | 8.616E-01 | 8.900E-01 | 8.873E-01 | 8.791E-01 | 8.824E-01 |
|  |  | STD | 2.238E-02 | 2.406E-02 | 3.056E-02 | 3.182E-02 | 3.139E-02 | **2.175E-02** | 4.132E-02 | 4.291E-02 | 3.559E-02 |
|  | 20 | AVG | **9.391E-01** | 9.311E-01 | 9.266E-01 | 9.132E-01 | 8.883E-01 | 9.149E-01 | 9.041E-01 | 9.043E-01 | 9.209E-01 |
|  |  | STD | **1.385E-02** | 1.700E-02 | 3.069E-02 | 4.659E-02 | 3.750E-02 | 2.408E-02 | 2.762E-02 | 3.926E-02 | 2.107E-02 |
|  | 25 | AVG | **9.462E-01** | 9.348E-01 | 9.381E-01 | 9.374E-01 | 9.090E-01 | 9.328E-01 | 9.289E-01 | 9.291E-01 | 9.203E-01 |
|  |  | STD | 1.907E-02 | 2.313E-02 | 2.080E-02 | 2.335E-02 | 3.816E-02 | **1.606E-02** | 1.982E-02 | 2.512E-02 | 2.821E-02 |
| D | 4 | AVG | 7.579E-01 | **7.586E-01** | 7.224E-01 | 6.730E-01 | 6.996E-01 | 6.970E-01 | 6.837E-01 | 6.936E-01 | 6.952E-01 |
|  |  | STD | **3.302E-02** | 3.405E-02 | 4.741E-02 | 5.102E-02 | 5.262E-02 | 4.134E-02 | 6.803E-02 | 4.676E-02 | 3.983E-02 |
|  | 5 | AVG | **7.889E-01** | 7.851E-01 | 7.783E-01 | 7.236E-01 | 7.010E-01 | 7.377E-01 | 7.226E-01 | 7.284E-01 | 7.383E-01 |
|  |  | STD | **3.633E-02** | 3.866E-02 | 4.213E-02 | 5.184E-02 | 4.805E-02 | 3.989E-02 | 6.870E-02 | 4.774E-02 | 4.631E-02 |
|  | 6 | AVG | **8.251E-01** | 8.095E-01 | 7.908E-01 | 7.687E-01 | 7.203E-01 | 7.539E-01 | 7.765E-01 | 7.671E-01 | 7.544E-01 |
|  |  | STD | **2.466E-02** | 2.881E-02 | 4.927E-02 | 4.436E-02 | 4.426E-02 | 4.735E-02 | 5.044E-02 | 4.670E-02 | 6.362E-02 |
|  | 15 | AVG | **9.413E-01** | 9.293E-01 | 9.288E-01 | 9.061E-01 | 8.657E-01 | 9.000E-01 | 8.855E-01 | 9.077E-01 | 9.061E-01 |
|  |  | STD | **2.185E-02** | 3.513E-02 | 3.048E-02 | 3.200E-02 | 5.093E-02 | 3.496E-02 | 3.164E-02 | 2.908E-02 | 3.023E-02 |
|  | 20 | AVG | 9.489E-01 | **9.528E-01** | 9.313E-01 | 9.284E-01 | 9.085E-01 | 9.269E-01 | 9.275E-01 | 9.335E-01 | 9.244E-01 |
|  |  | STD | **1.932E-02** | 2.222E-02 | 4.637E-02 | 3.684E-02 | 4.925E-02 | 2.216E-02 | 3.043E-02 | 2.400E-02 | 3.086E-02 |
|  | 25 | AVG | 9.592E-01 | **9.598E-01** | 9.524E-01 | 9.554E-01 | 9.304E-01 | 9.545E-01 | 9.463E-01 | 9.513E-01 | 9.426E-01 |
|  |  | STD | 1.858E-02 | **1.563E-02** | 2.046E-02 | 2.197E-02 | 3.477E-02 | 1.734E-02 | 2.328E-02 | 2.264E-02 | 2.874E-02 |
| E | 4 | AVG | 7.441E-01 | **7.462E-01** | 7.313E-01 | 7.151E-01 | 7.184E-01 | 7.249E-01 | 7.433E-01 | 7.187E-01 | 7.284E-01 |
|  |  | STD | **2.177E-02** | 2.263E-02 | 3.261E-02 | 3.192E-02 | 2.924E-02 | 2.583E-02 | 2.336E-02 | 3.073E-02 | 4.302E-02 |
|  | 5 | AVG | **7.737E-01** | 7.706E-01 | 7.528E-01 | 7.363E-01 | 7.285E-01 | 7.434E-01 | 7.588E-01 | 7.362E-01 | 7.392E-01 |
|  |  | STD | 2.108E-02 | **2.067E-02** | 3.561E-02 | 4.177E-02 | 4.547E-02 | 3.506E-02 | 3.015E-02 | 3.854E-02 | 2.865E-02 |
|  | 6 | AVG | **7.965E-01** | 7.943E-01 | 7.783E-01 | 7.457E-01 | 7.370E-01 | 7.504E-01 | 7.612E-01 | 7.423E-01 | 7.719E-01 |
|  |  | STD | **2.259E-02** | 2.792E-02 | 3.728E-02 | 4.880E-02 | 3.747E-02 | 3.056E-02 | 3.441E-02 | 4.282E-02 | 2.660E-02 |
|  | 15 | AVG | **9.120E-01** | 9.091E-01 | 8.893E-01 | 8.710E-01 | 8.326E-01 | 8.650E-01 | 8.592E-01 | 8.700E-01 | 8.701E-01 |
|  |  | STD | **1.992E-02** | 2.332E-02 | 3.816E-02 | 3.415E-02 | 4.267E-02 | 3.464E-02 | 3.960E-02 | 2.946E-02 | 3.792E-02 |
|  | 20 | AVG | **9.332E-01** | 9.241E-01 | 9.110E-01 | 8.953E-01 | 8.647E-01 | 8.946E-01 | 8.862E-01 | 9.112E-01 | 9.082E-01 |
|  |  | STD | **1.915E-02** | 2.750E-02 | 2.954E-02 | 3.600E-02 | 4.001E-02 | 3.026E-02 | 3.817E-02 | 2.729E-02 | 2.964E-02 |
|  | 25 | AVG | **9.442E-01** | 9.356E-01 | 9.323E-01 | 9.287E-01 | 8.983E-01 | 9.238E-01 | 9.128E-01 | 9.250E-01 | 9.222E-01 |
|  |  | STD | 2.221E-02 | **1.824E-02** | 2.901E-02 | 2.761E-02 | 3.153E-02 | 2.439E-02 | 2.679E-02 | 2.992E-02 | 2.692E-02 |
| F | 4 | AVG | 7.454E-01 | 7.463E-01 | 7.337E-01 | 7.302E-01 | 7.228E-01 | 7.163E-01 | **7.530E-01** | 7.226E-01 | 7.205E-01 |
|  |  | STD | **4.463E-03** | 1.181E-02 | 3.326E-02 | 4.351E-02 | 5.414E-02 | 3.746E-02 | 1.965E-02 | 4.693E-02 | 4.061E-02 |
|  | 5 | AVG | 7.768E-01 | 7.789E-01 | **7.827E-01** | 7.577E-01 | 7.289E-01 | 7.353E-01 | 7.638E-01 | 7.525E-01 | 7.367E-01 |
|  |  | STD | **1.431E-02** | 2.712E-02 | 2.436E-02 | 5.439E-02 | 5.466E-02 | 4.458E-02 | 4.243E-02 | 4.688E-02 | 3.214E-02 |
|  | 6 | AVG | **8.056E-01** | 8.034E-01 | 7.823E-01 | 7.652E-01 | 7.313E-01 | 7.578E-01 | 7.681E-01 | 7.721E-01 | 7.756E-01 |
|  |  | STD | **2.565E-02** | 2.652E-02 | 4.555E-02 | 4.647E-02 | 5.135E-02 | 3.771E-02 | 4.930E-02 | 3.831E-02 | 4.277E-02 |
|  | 15 | AVG | 9.039E-01 | **9.150E-01** | 9.058E-01 | 8.864E-01 | 8.340E-01 | 8.720E-01 | 8.756E-01 | 8.728E-01 | 8.815E-01 |
|  |  | STD | 3.213E-02 | **2.488E-02** | 3.424E-02 | 4.702E-02 | 4.927E-02 | 4.381E-02 | 3.423E-02 | 4.547E-02 | 3.195E-02 |
|  | 20 | AVG | **9.362E-01** | 9.333E-01 | 9.322E-01 | 9.164E-01 | 8.757E-01 | 9.085E-01 | 8.943E-01 | 9.103E-01 | 9.032E-01 |
|  |  | STD | **2.366E-02** | 2.840E-02 | 2.872E-02 | 4.075E-02 | 4.769E-02 | 2.613E-02 | 4.798E-02 | 4.018E-02 | 3.164E-02 |
|  | 25 | AVG | **9.437E-01** | 9.422E-01 | 9.355E-01 | 9.335E-01 | 8.958E-01 | 9.280E-01 | 9.170E-01 | 9.312E-01 | 9.233E-01 |
|  |  | STD | 2.403E-02 | **2.116E-02** | 2.494E-02 | 3.080E-02 | 4.155E-02 | 2.948E-02 | 3.078E-02 | 3.772E-02 | 2.703E-02 |
| G | 4 | AVG | 7.327E-01 | 7.266E-01 | 7.197E-01 | 6.914E-01 | 6.927E-01 | 6.934E-01 | **7.361E-01** | 6.921E-01 | 6.870E-01 |
|  |  | STD | **1.717E-02** | 2.947E-02 | 3.260E-02 | 4.300E-02 | 4.025E-02 | 2.990E-02 | 2.190E-02 | 3.292E-02 | 3.681E-02 |
|  | 5 | AVG | **7.643E-01** | 7.508E-01 | 7.439E-01 | 7.151E-01 | 7.004E-01 | 7.165E-01 | 7.513E-01 | 7.090E-01 | 7.276E-01 |
|  |  | STD | **1.966E-02** | 3.778E-02 | 4.703E-02 | 4.318E-02 | 4.160E-02 | 2.815E-02 | 2.603E-02 | 4.352E-02 | 3.045E-02 |
|  | 6 | AVG | **7.943E-01** | 7.835E-01 | 7.747E-01 | 7.362E-01 | 7.116E-01 | 7.415E-01 | 7.739E-01 | 7.498E-01 | 7.422E-01 |
|  |  | STD | **2.460E-02** | 3.089E-02 | 3.422E-02 | 4.221E-02 | 4.625E-02 | 2.965E-02 | 4.092E-02 | 3.907E-02 | 4.591E-02 |
|  | 15 | AVG | **9.085E-01** | 8.945E-01 | 8.874E-01 | 8.657E-01 | 8.230E-01 | 8.697E-01 | 8.599E-01 | 8.655E-01 | 8.645E-01 |
|  |  | STD | **2.202E-02** | 3.310E-02 | 3.370E-02 | 4.053E-02 | 3.588E-02 | 3.324E-02 | 4.271E-02 | 3.472E-02 | 3.298E-02 |
|  | 20 | AVG | **9.342E-01** | 9.205E-01 | 9.094E-01 | 9.203E-01 | 8.748E-01 | 9.015E-01 | 8.986E-01 | 9.070E-01 | 9.037E-01 |
|  |  | STD | 2.683E-02 | 2.649E-02 | 2.922E-02 | **2.120E-02** | 3.065E-02 | 2.645E-02 | 3.446E-02 | 3.345E-02 | 2.320E-02 |
|  | 25 | AVG | **9.387E-01** | 9.322E-01 | 9.321E-01 | 9.290E-01 | 9.007E-01 | 9.243E-01 | 9.258E-01 | 9.276E-01 | 9.206E-01 |
|  |  | STD | 2.099E-02 | **2.027E-02** | 3.243E-02 | 2.691E-02 | 4.018E-02 | 2.329E-02 | 2.266E-02 | 2.677E-02 | 2.777E-02 |
| H | 4 | AVG | **7.774E-01** | 7.705E-01 | 7.547E-01 | 7.261E-01 | 7.482E-01 | 7.159E-01 | 7.437E-01 | 7.223E-01 | 7.180E-01 |
|  |  | STD | 4.041E-02 | 3.839E-02 | 5.113E-02 | 5.044E-02 | 4.159E-02 | 4.171E-02 | 5.447E-02 | 3.916E-02 | **3.643E-02** |
|  | 5 | AVG | 8.022E-01 | **8.096E-01** | 7.774E-01 | 7.566E-01 | 7.412E-01 | 7.395E-01 | 7.697E-01 | 7.420E-01 | 7.438E-01 |
|  |  | STD | 4.084E-02 | **3.629E-02** | 4.522E-02 | 4.994E-02 | 4.982E-02 | 5.201E-02 | 4.876E-02 | 4.327E-02 | 3.883E-02 |
|  | 6 | AVG | **8.324E-01** | 8.320E-01 | 8.103E-01 | 7.822E-01 | 7.635E-01 | 7.955E-01 | 7.933E-01 | 7.811E-01 | 7.822E-01 |
|  |  | STD | **3.135E-02** | 3.443E-02 | 3.614E-02 | 5.436E-02 | 5.317E-02 | 4.216E-02 | 3.617E-02 | 4.618E-02 | 3.392E-02 |
|  | 15 | AVG | 9.401E-01 | **9.421E-01** | 9.142E-01 | 8.977E-01 | 8.709E-01 | 8.851E-01 | 8.924E-01 | 8.986E-01 | 9.053E-01 |
|  |  | STD | **2.177E-02** | 2.675E-02 | 3.271E-02 | 4.122E-02 | 4.296E-02 | 3.264E-02 | 4.372E-02 | 3.366E-02 | 2.777E-02 |
|  | 20 | AVG | 9.508E-01 | **9.556E-01** | 9.420E-01 | 9.204E-01 | 8.985E-01 | 9.279E-01 | 9.180E-01 | 9.428E-01 | 9.224E-01 |
|  |  | STD | 2.760E-02 | **1.799E-02** | 2.909E-02 | 5.241E-02 | 4.492E-02 | 2.197E-02 | 3.792E-02 | 2.952E-02 | 2.320E-02 |
|  | 25 | AVG | 9.583E-01 | **9.587E-01** | 9.526E-01 | 9.468E-01 | 9.223E-01 | 9.467E-01 | 9.361E-01 | 9.511E-01 | 9.465E-01 |
|  |  | STD | **1.806E-02** | 1.997E-02 | 2.372E-02 | 2.634E-02 | 4.036E-02 | 2.320E-02 | 3.486E-02 | 2.276E-02 | 2.227E-02 |

**Table A. 7.** The SSIM comparison results of all methods

| Image | Level | Item | MGACO-MIS | ACOR-MIS | MVO-MIS | HHO-MIS | SCA-MIS | BLPSO-MIS | IGWO-MIS | IWOA-MIS | CLPSO-MIS |
| --- | --- | --- | --- | --- | --- | --- | --- | --- | --- | --- | --- |
| A | 4 | AVG | 5.702E-01 | 5.665E-01 | 5.736E-01 | 4.982E-01 | 5.090E-01 | 5.138E-01 | **5.835E-01** | 5.094E-01 | 5.165E-01 |
|  |  | STD | 4.994E-02 | 4.811E-02 | 5.236E-02 | 6.125E-02 | 5.668E-02 | **4.015E-02** | 4.937E-02 | 5.853E-02 | 4.414E-02 |
|  | 5 | AVG | 6.170E-01 | 6.097E-01 | **6.240E-01** | 5.772E-01 | 5.407E-01 | 5.762E-01 | 6.207E-01 | 5.595E-01 | 5.805E-01 |
|  |  | STD | 5.441E-02 | 5.034E-02 | 4.872E-02 | 7.855E-02 | 7.142E-02 | **4.370E-02** | 4.565E-02 | 7.464E-02 | 4.981E-02 |
|  | 6 | AVG | **6.672E-01** | 6.610E-01 | 6.557E-01 | 6.203E-01 | 5.769E-01 | 6.150E-01 | 6.279E-01 | 6.087E-01 | 6.103E-01 |
|  |  | STD | 4.383E-02 | 4.055E-02 | 5.611E-02 | 6.638E-02 | 8.408E-02 | **3.497E-02** | 5.137E-02 | 6.564E-02 | 5.280E-02 |
|  | 15 | AVG | **8.581E-01** | 8.576E-01 | 8.414E-01 | 8.079E-01 | 7.520E-01 | 8.103E-01 | 7.909E-01 | 8.133E-01 | 8.075E-01 |
|  |  | STD | 3.121E-02 | **2.966E-02** | 4.765E-02 | 5.231E-02 | 5.438E-02 | 4.879E-02 | 5.525E-02 | 4.211E-02 | 4.211E-02 |
|  | 20 | AVG | **8.887E-01** | 8.857E-01 | 8.673E-01 | 8.554E-01 | 8.088E-01 | 8.610E-01 | 8.441E-01 | 8.674E-01 | 8.489E-01 |
|  |  | STD | **2.751E-02** | 2.860E-02 | 3.824E-02 | 5.552E-02 | 6.133E-02 | 3.410E-02 | 4.791E-02 | 3.913E-02 | 3.926E-02 |
|  | 25 | AVG | 9.059E-01 | **9.129E-01** | 8.892E-01 | 8.886E-01 | 8.605E-01 | 8.893E-01 | 8.868E-01 | 8.913E-01 | 8.862E-01 |
|  |  | STD | **1.797E-02** | 2.325E-02 | 3.710E-02 | 3.980E-02 | 4.699E-02 | 2.391E-02 | 2.660E-02 | 3.502E-02 | 3.640E-02 |
| B | 4 | AVG | **7.250E-01** | 7.135E-01 | 6.959E-01 | 6.822E-01 | 6.769E-01 | 6.570E-01 | 7.125E-01 | 6.571E-01 | 6.499E-01 |
|  |  | STD | **1.911E-02** | 3.015E-02 | 5.110E-02 | 4.641E-02 | 3.705E-02 | 4.715E-02 | 4.355E-02 | 5.938E-02 | 7.499E-02 |
|  | 5 | AVG | 7.258E-01 | **7.280E-01** | 7.049E-01 | 6.806E-01 | 6.947E-01 | 6.934E-01 | 7.069E-01 | 6.847E-01 | 6.822E-01 |
|  |  | STD | **2.917E-02** | 3.235E-02 | 4.240E-02 | 6.838E-02 | 3.268E-02 | 3.998E-02 | 5.461E-02 | 4.735E-02 | 4.593E-02 |
|  | 6 | AVG | **7.544E-01** | 7.491E-01 | 7.425E-01 | 6.999E-01 | 6.991E-01 | 7.060E-01 | 7.113E-01 | 7.028E-01 | 7.027E-01 |
|  |  | STD | **1.998E-02** | 2.857E-02 | 3.695E-02 | 6.100E-02 | 5.960E-02 | 2.918E-02 | 4.706E-02 | 5.790E-02 | 5.559E-02 |
|  | 15 | AVG | **8.463E-01** | 8.399E-01 | 8.300E-01 | 8.121E-01 | 7.978E-01 | 8.048E-01 | 8.055E-01 | 8.129E-01 | 8.200E-01 |
|  |  | STD | **1.365E-02** | 2.223E-02 | 2.647E-02 | 2.810E-02 | 2.986E-02 | 2.473E-02 | 2.587E-02 | 2.548E-02 | 2.099E-02 |
|  | 20 | AVG | **8.778E-01** | 8.697E-01 | 8.592E-01 | 8.488E-01 | 8.239E-01 | 8.385E-01 | 8.373E-01 | 8.553E-01 | 8.512E-01 |
|  |  | STD | **1.460E-02** | 1.731E-02 | 2.241E-02 | 3.029E-02 | 3.120E-02 | 1.950E-02 | 2.148E-02 | 2.493E-02 | 1.531E-02 |
|  | 25 | AVG | **8.904E-01** | 8.800E-01 | 8.834E-01 | 8.739E-01 | 8.549E-01 | 8.670E-01 | 8.668E-01 | 8.733E-01 | 8.712E-01 |
|  |  | STD | **1.692E-02** | 2.559E-02 | 2.174E-02 | 2.416E-02 | 3.009E-02 | 1.715E-02 | 2.271E-02 | 2.286E-02 | 2.078E-02 |
| C | 4 | AVG | 5.631E-01 | 5.550E-01 | 5.472E-01 | 5.612E-01 | **6.426E-01** | 5.304E-01 | 5.627E-01 | 5.873E-01 | 5.569E-01 |
|  |  | STD | 2.594E-02 | 2.936E-02 | 7.525E-02 | 7.568E-02 | 1.197E-01 | **2.350E-02** | 4.750E-02 | 7.216E-02 | 6.743E-02 |
|  | 5 | AVG | 6.120E-01 | 5.995E-01 | 5.826E-01 | 6.074E-01 | **6.891E-01** | 5.912E-01 | 6.730E-01 | 5.958E-01 | 6.002E-01 |
|  |  | STD | 7.011E-02 | 7.286E-02 | 9.277E-02 | 1.086E-01 | 7.386E-02 | 8.223E-02 | 9.499E-02 | 8.863E-02 | **6.794E-02** |
|  | 6 | AVG | 6.521E-01 | 6.429E-01 | 6.326E-01 | 6.643E-01 | **7.133E-01** | 6.478E-01 | 6.755E-01 | 6.701E-01 | 6.606E-01 |
|  |  | STD | 7.428E-02 | 7.161E-02 | 8.293E-02 | 9.831E-02 | 7.961E-02 | 7.377E-02 | 8.851E-02 | 8.720E-02 | **6.627E-02** |
|  | 15 | AVG | **8.643E-01** | 8.473E-01 | 8.397E-01 | 8.313E-01 | 8.033E-01 | 8.337E-01 | 8.346E-01 | 8.167E-01 | 8.347E-01 |
|  |  | STD | **2.037E-02** | 3.933E-02 | 4.194E-02 | 5.507E-02 | 6.168E-02 | 3.622E-02 | 4.694E-02 | 6.435E-02 | 3.560E-02 |
|  | 20 | AVG | **8.896E-01** | 8.835E-01 | 8.777E-01 | 8.462E-01 | 8.504E-01 | 8.741E-01 | 8.590E-01 | 8.346E-01 | 8.664E-01 |
|  |  | STD | 2.241E-02 | **1.792E-02** | 4.251E-02 | 6.945E-02 | 3.809E-02 | 1.853E-02 | 2.847E-02 | 7.092E-02 | 4.426E-02 |
|  | 25 | AVG | **9.043E-01** | 8.976E-01 | 8.863E-01 | 9.003E-01 | 8.585E-01 | 8.861E-01 | 8.874E-01 | 8.828E-01 | 8.726E-01 |
|  |  | STD | 1.945E-02 | **1.898E-02** | 3.905E-02 | 2.328E-02 | 4.994E-02 | 2.289E-02 | 2.319E-02 | 3.798E-02 | 4.055E-02 |
| D | 4 | AVG | 6.462E-01 | **6.477E-01** | 6.072E-01 | 5.449E-01 | 5.994E-01 | 5.863E-01 | 5.838E-01 | 5.723E-01 | 5.854E-01 |
|  |  | STD | 2.662E-02 | **2.236E-02** | 5.107E-02 | 9.469E-02 | 6.770E-02 | 5.102E-02 | 7.295E-02 | 5.083E-02 | 5.350E-02 |
|  | 5 | AVG | **6.558E-01** | 6.554E-01 | 6.396E-01 | 6.057E-01 | 6.004E-01 | 6.238E-01 | 6.067E-01 | 6.126E-01 | 6.143E-01 |
|  |  | STD | 3.327E-02 | **3.136E-02** | 4.119E-02 | 4.925E-02 | 5.489E-02 | 3.528E-02 | 5.856E-02 | 4.804E-02 | 4.772E-02 |
|  | 6 | AVG | **6.791E-01** | 6.667E-01 | 6.580E-01 | 6.432E-01 | 6.265E-01 | 6.393E-01 | 6.460E-01 | 6.445E-01 | 6.367E-01 |
|  |  | STD | 3.127E-02 | **2.374E-02** | 4.537E-02 | 4.651E-02 | 3.957E-02 | 4.065E-02 | 5.838E-02 | 3.646E-02 | 5.458E-02 |
|  | 15 | AVG | **8.280E-01** | 8.145E-01 | 8.241E-01 | 8.006E-01 | 7.603E-01 | 7.894E-01 | 7.755E-01 | 8.010E-01 | 7.966E-01 |
|  |  | STD | **3.114E-02** | 3.506E-02 | 3.312E-02 | 4.560E-02 | 4.850E-02 | 3.620E-02 | 3.689E-02 | 3.601E-02 | 3.582E-02 |
|  | 20 | AVG | 8.520E-01 | **8.602E-01** | 8.405E-01 | 8.389E-01 | 8.112E-01 | 8.312E-01 | 8.327E-01 | 8.394E-01 | 8.318E-01 |
|  |  | STD | 2.698E-02 | 3.050E-02 | 5.189E-02 | 4.137E-02 | 4.054E-02 | **2.152E-02** | 3.497E-02 | 3.547E-02 | 3.548E-02 |
|  | 25 | AVG | 8.774E-01 | **8.794E-01** | 8.739E-01 | 8.786E-01 | 8.449E-01 | 8.718E-01 | 8.607E-01 | 8.708E-01 | 8.606E-01 |
|  |  | STD | 2.660E-02 | 2.546E-02 | 2.960E-02 | 2.975E-02 | 4.211E-02 | **2.446E-02** | 2.852E-02 | 3.261E-02 | 3.390E-02 |
| E | 4 | AVG | 6.944E-01 | **7.084E-01** | 6.818E-01 | 6.334E-01 | 6.644E-01 | 6.539E-01 | 7.040E-01 | 6.502E-01 | 6.549E-01 |
|  |  | STD | 4.206E-02 | **2.874E-02** | 5.366E-02 | 6.413E-02 | 4.109E-02 | 4.960E-02 | 3.646E-02 | 5.456E-02 | 8.436E-02 |
|  | 5 | AVG | **7.226E-01** | 7.149E-01 | 6.924E-01 | 6.700E-01 | 6.700E-01 | 6.801E-01 | 7.081E-01 | 6.647E-01 | 6.846E-01 |
|  |  | STD | **2.041E-02** | 2.310E-02 | 3.604E-02 | 6.621E-02 | 6.504E-02 | 4.828E-02 | 4.637E-02 | 6.402E-02 | 4.094E-02 |
|  | 6 | AVG | **7.261E-01** | 7.258E-01 | 7.077E-01 | 6.690E-01 | 6.957E-01 | 6.926E-01 | 6.874E-01 | 6.891E-01 | 7.103E-01 |
|  |  | STD | **2.208E-02** | 2.593E-02 | 3.366E-02 | 8.513E-02 | 4.372E-02 | 3.626E-02 | 5.186E-02 | 3.907E-02 | 3.913E-02 |
|  | 15 | AVG | **8.313E-01** | 8.253E-01 | 8.168E-01 | 8.068E-01 | 7.760E-01 | 7.955E-01 | 7.881E-01 | 7.998E-01 | 8.035E-01 |
|  |  | STD | 2.550E-02 | **2.019E-02** | 3.003E-02 | 2.772E-02 | 3.546E-02 | 2.543E-02 | 3.270E-02 | 2.175E-02 | 2.867E-02 |
|  | 20 | AVG | **8.634E-01** | 8.570E-01 | 8.416E-01 | 8.334E-01 | 8.060E-01 | 8.357E-01 | 8.268E-01 | 8.499E-01 | 8.431E-01 |
|  |  | STD | **1.743E-02** | 2.900E-02 | 2.900E-02 | 3.551E-02 | 3.387E-02 | 2.476E-02 | 3.324E-02 | 2.197E-02 | 2.686E-02 |
|  | 25 | AVG | **8.846E-01** | 8.694E-01 | 8.698E-01 | 8.680E-01 | 8.409E-01 | 8.657E-01 | 8.534E-01 | 8.681E-01 | 8.633E-01 |
|  |  | STD | 1.952E-02 | **1.780E-02** | 2.792E-02 | 2.616E-02 | 2.761E-02 | 2.051E-02 | 2.451E-02 | 2.255E-02 | 2.443E-02 |
| F | 4 | AVG | 6.901E-01 | 6.859E-01 | 6.755E-01 | 6.705E-01 | 6.606E-01 | 6.581E-01 | **6.973E-01** | 6.468E-01 | 6.592E-01 |
|  |  | STD | **8.531E-03** | 1.441E-02 | 5.575E-02 | 4.478E-02 | 8.001E-02 | 4.835E-02 | 1.871E-02 | 8.164E-02 | 5.279E-02 |
|  | 5 | AVG | 7.084E-01 | 7.081E-01 | **7.129E-01** | 6.818E-01 | 6.739E-01 | 6.619E-01 | 7.064E-01 | 6.897E-01 | 6.810E-01 |
|  |  | STD | **1.369E-02** | 2.547E-02 | 2.002E-02 | 7.287E-02 | 5.923E-02 | 8.571E-02 | 4.030E-02 | 5.809E-02 | 3.118E-02 |
|  | 6 | AVG | 7.234E-01 | **7.243E-01** | 7.094E-01 | 6.941E-01 | 6.829E-01 | 6.930E-01 | 7.054E-01 | 7.019E-01 | 7.096E-01 |
|  |  | STD | 2.497E-02 | **1.977E-02** | 3.998E-02 | 4.739E-02 | 6.255E-02 | 3.447E-02 | 5.395E-02 | 3.565E-02 | 4.482E-02 |
|  | 15 | AVG | 8.196E-01 | **8.330E-01** | 8.295E-01 | 8.115E-01 | 7.716E-01 | 8.028E-01 | 8.053E-01 | 7.993E-01 | 8.098E-01 |
|  |  | STD | 2.947E-02 | **2.422E-02** | 2.853E-02 | 4.306E-02 | 3.746E-02 | 3.132E-02 | 3.275E-02 | 3.737E-02 | 2.766E-02 |
|  | 20 | AVG | 8.627E-01 | 8.642E-01 | **8.648E-01** | 8.495E-01 | 8.172E-01 | 8.393E-01 | 8.325E-01 | 8.437E-01 | 8.370E-01 |
|  |  | STD | 2.449E-02 | 2.926E-02 | 3.004E-02 | 3.995E-02 | 3.945E-02 | 2.496E-02 | 4.689E-02 | 3.981E-02 | **2.324E-02** |
|  | 25 | AVG | **8.826E-01** | 8.780E-01 | 8.766E-01 | 8.764E-01 | 8.404E-01 | 8.644E-01 | 8.604E-01 | 8.698E-01 | 8.633E-01 |
|  |  | STD | 2.480E-02 | **2.096E-02** | 2.514E-02 | 3.155E-02 | 3.518E-02 | 2.667E-02 | 2.643E-02 | 3.691E-02 | 2.308E-02 |
| G | 4 | AVG | 6.726E-01 | 6.642E-01 | 6.519E-01 | 5.753E-01 | 6.689E-01 | 5.769E-01 | **6.907E-01** | 6.115E-01 | 6.120E-01 |
|  |  | STD | 5.856E-02 | 7.766E-02 | 9.046E-02 | 1.313E-01 | 5.342E-02 | 8.485E-02 | **4.400E-02** | 7.565E-02 | 9.372E-02 |
|  | 5 | AVG | 7.052E-01 | 6.797E-01 | 6.607E-01 | 6.337E-01 | 6.651E-01 | 6.358E-01 | **7.106E-01** | 6.247E-01 | 6.478E-01 |
|  |  | STD | **2.767E-02** | 6.227E-02 | 8.227E-02 | 9.941E-02 | 6.137E-02 | 7.445E-02 | 3.576E-02 | 1.046E-01 | 9.219E-02 |
|  | 6 | AVG | **7.289E-01** | 7.172E-01 | 7.066E-01 | 6.472E-01 | 6.634E-01 | 6.786E-01 | 7.153E-01 | 6.900E-01 | 6.815E-01 |
|  |  | STD | **2.017E-02** | 3.209E-02 | 5.652E-02 | 9.586E-02 | 7.029E-02 | 6.665E-02 | 5.968E-02 | 7.211E-02 | 6.039E-02 |
|  | 15 | AVG | **8.402E-01** | 8.272E-01 | 8.199E-01 | 8.046E-01 | 7.705E-01 | 8.066E-01 | 8.039E-01 | 8.026E-01 | 7.991E-01 |
|  |  | STD | **1.865E-02** | 2.629E-02 | 3.437E-02 | 4.676E-02 | 4.159E-02 | 2.922E-02 | 3.636E-02 | 4.608E-02 | 2.604E-02 |
|  | 20 | AVG | **8.747E-01** | 8.576E-01 | 8.484E-01 | 8.613E-01 | 8.220E-01 | 8.433E-01 | 8.441E-01 | 8.498E-01 | 8.444E-01 |
|  |  | STD | 2.061E-02 | 2.761E-02 | 3.339E-02 | 2.633E-02 | 2.430E-02 | 2.110E-02 | 3.205E-02 | 2.837E-02 | **1.969E-02** |
|  | 25 | AVG | **8.854E-01** | 8.757E-01 | 8.767E-01 | 8.784E-01 | 8.520E-01 | 8.689E-01 | 8.715E-01 | 8.769E-01 | 8.654E-01 |
|  |  | STD | **1.947E-02** | 2.320E-02 | 3.430E-02 | 2.895E-02 | 3.460E-02 | 2.295E-02 | 2.548E-02 | 2.252E-02 | 2.372E-02 |
| H | 4 | AVG | **6.581E-01** | 6.548E-01 | 6.379E-01 | 5.976E-01 | 6.367E-01 | 6.035E-01 | 6.298E-01 | 5.958E-01 | 6.166E-01 |
|  |  | STD | **2.936E-02** | 3.413E-02 | 5.058E-02 | 5.561E-02 | 5.423E-02 | 6.108E-02 | 5.720E-02 | 3.773E-02 | 5.071E-02 |
|  | 5 | AVG | 6.643E-01 | **6.673E-01** | 6.494E-01 | 6.144E-01 | 6.325E-01 | 6.242E-01 | 6.445E-01 | 6.149E-01 | 6.244E-01 |
|  |  | STD | 3.365E-02 | **3.243E-02** | 4.079E-02 | 6.321E-02 | 5.641E-02 | 5.175E-02 | 3.485E-02 | 4.923E-02 | 4.115E-02 |
|  | 6 | AVG | 6.872E-01 | **6.882E-01** | 6.688E-01 | 6.499E-01 | 6.573E-01 | 6.694E-01 | 6.636E-01 | 6.567E-01 | 6.515E-01 |
|  |  | STD | **2.685E-02** | 2.721E-02 | 3.931E-02 | 5.641E-02 | 5.267E-02 | 3.827E-02 | 3.647E-02 | 3.116E-02 | 4.135E-02 |
|  | 15 | AVG | **8.271E-01** | 8.252E-01 | 8.026E-01 | 7.917E-01 | 7.587E-01 | 7.790E-01 | 7.861E-01 | 7.872E-01 | 7.965E-01 |
|  |  | STD | **2.709E-02** | 3.330E-02 | 3.130E-02 | 4.298E-02 | 4.531E-02 | 2.953E-02 | 4.055E-02 | 3.697E-02 | 2.933E-02 |
|  | 20 | AVG | 8.588E-01 | **8.610E-01** | 8.479E-01 | 8.302E-01 | 8.003E-01 | 8.333E-01 | 8.260E-01 | 8.491E-01 | 8.260E-01 |
|  |  | STD | 2.789E-02 | **2.302E-02** | 4.049E-02 | 4.989E-02 | 4.695E-02 | 2.545E-02 | 3.445E-02 | 3.767E-02 | 2.791E-02 |
|  | 25 | AVG | 8.767E-01 | **8.804E-01** | 8.719E-01 | 8.684E-01 | 8.345E-01 | 8.639E-01 | 8.491E-01 | 8.689E-01 | 8.617E-01 |
|  |  | STD | **2.504E-02** | 2.625E-02 | 2.661E-02 | 3.263E-02 | 3.920E-02 | 2.720E-02 | 3.487E-02 | 3.039E-02 | 2.617E-02 |

**Table A. 8.** Specific segmentation thresholds at low threshold level

| Image | Level | MGACO | ACOR | MVO | HHO | SCA |
| --- | --- | --- | --- | --- | --- | --- |
| A | 4 | 37,62,111,161 | 46,80,107,158 | 36,62,112,171 | 31,63,100,146 | 17,28,60,135 |
|  | 5 | 43,84,118,146,183 | 46,87,120,146,183 | 46,86,119,145,185 | 24,37,86,109,185 | 26,42,97,120,161 |
|  | 6 | 33,63,88,107,147,186 | 24,43,76,107,144,181 | 46,91,129,167,209,254 | 49,93,140,176,212,254 | 25,37,50,110,168,186 |
| B | 4 | 34,63,108,158 | 34,63,112,161 | 34,63,107,157 | 27,58,100,155 | 24,37,69,164 |
|  | 5 | 28,50,91,135,177 | 33,63,102,140,179 | 28,50,92,139,182 | 26,52,81,118,178 | 13,37,74,107,151 |
|  | 6 | 16,35,54,92,133,174 | 17,40,63,102,140,179 | 26,50,79,115,148,188 | 16,37,83,109,153,190 | 24,85,103,115,159,206 |
| C | 4 | 45,75,101,154 | 53,95,136,177 | 53,96,137,178 | 59,104,145,192 | 48,112,131,188 |
|  | 5 | 44,73,101,140,179 | 45,75,101,140,179 | 37,62,90,137,178 | 44,73,100,151,192 | 23,62,97,148,211 |
|  | 6 | 36,62,81,117,154,189 | 38,63,82,115,148,184 | 45,70,99,126,154,190 | 31,53,76,113,153,198 | 30,55,71,97,117,209 |
| D | 4 | 74,148,204,253 | 32,57,82,141 | 65,154,208,253 | 88,169,214,253 | 144,171,224,253 |
|  | 5 | 55,110,166,222,253 | 57,113,168,223,253 | 52,97,170,222,253 | 74,154,196,232,253 | 36,106,187,212,253 |
|  | 6 | 43,91,136,184,222,253 | 47,95,141,186,230,253 | 62,117,152,188,227,200 | 55,146,178,211,242,200 | 69,101,149,159,214,200 |
| E | 4 | 30,61,95,150 | 30,61,95,140 | 28,58,94,143 | 41,67,95,143 | 22,36,118,165 |
|  | 5 | 25,49,73,119,165 | 30,60,90,132,174 | 63,110,132,158,185 | 67,92,110,137,180 | 41,62,99,113,141 |
|  | 6 | 22,43,66,101,140,178 | 24,44,66,104,143,182 | 23,42,64,103,145,193 | 15,37,66,110,140,168 | 52,73,86,95,119,180 |
| F | 4 | 42,69,116,164 | 42,69,117,164 | 41,69,115,164 | 37,69,111,168 | 34,67,99,149 |
|  | 5 | 31,59,85,129,172 | 31,58,85,127,171 | 30,63,94,136,175 | 25,48,89,123,184 | 19,31,55,144,163 |
|  | 6 | 24,47,70,107,145,185 | 23,46,70,108,147,185 | 23,44,62,91,131,169 | 37,60,79,100,136,189 | 40,95,153,178,207,254 |
| G | 4 | 41,74,107,158 | 42,75,107,158 | 38,72,107,163 | 39,65,107,157 | 35,81,99,128 |
|  | 5 | 32,61,90,132,174 | 32,61,90,132,174 | 32,61,91,140,170 | 52,77,129,183,199 | 58,73,100,138,149 |
|  | 6 | 29,55,78,113,150,186 | 25,47,66,105,143,181 | 26,51,69,98,140,192 | 18,34,50,69,154,181 | 53,97,129,165,188,226 |
| H | 4 | 38,77,108,154 | 38,77,108,158 | 42,77,107,156 | 47,79,109,140 | 85,130,169,200 |
|  | 5 | 46,69,92,117,164 | 30,60,88,116,164 | 32,57,84,116,171 | 59,100,145,170,198 | 26,81,152,187,202 |
|  | 6 | 43,86,129,158,184,204 | 28,55,83,108,145,182 | 54,91,132,159,187,211 | 47,103,151,184,216,200 | 21,58,94,117,136,198 |

**Table A. 8. (continued)** Specific segmentation thresholds at low threshold level

| Image | Level | BLPSO | IGWO | IWOA | CLPSO |
| --- | --- | --- | --- | --- | --- |
| A | 4 | 67,93,144,176 | 50,77,106,147 | 65,113,138,173 | 58,101,139,179 |
|  | 5 | 41,82,143,177,206 | 23,42,81,124,172 | 58,99,149,191,254 | 43,96,150,212,254 |
|  | 6 | 41,64,107,158,173,222 | 28,85,124,171,220,254 | 45,79,108,152,190,210 | 16,57,106,136,167,198 |
| B | 4 | 48,64,110,186 | 31,50,103,155 | 19,45,74,125 | 31,55,90,130 |
|  | 5 | 28,65,91,110,186 | 28,50,94,131,178 | 25,40,85,141,166 | 32,59,90,107,146 |
|  | 6 | 51,109,155,208,234,200 | 20,43,55,95,124,155 | 10,53,74,104,178,199 | 23,44,62,86,129,187 |
| C | 4 | 54,85,138,185 | 55,105,139,178 | 49,79,102,160 | 47,66,120,182 |
|  | 5 | 72,103,143,179,222 | 35,65,82,138,181 | 30,52,75,129,167 | 41,67,109,152,188 |
|  | 6 | 37,72,118,140,189,231 | 35,59,80,123,159,199 | 48,87,122,142,178,199 | 34,63,107,143,159,207 |
| D | 4 | 131,173,221,253 | 89,153,220,253 | 91,175,223,253 | 76,153,223,253 |
|  | 5 | 24,52,67,95,165 | 54,96,171,229,253 | 75,150,191,222,253 | 74,137,181,237,253 |
|  | 6 | 39,76,125,174,201,233 | 59,102,136,161,225,200 | 60,93,118,195,229,253 | 67,79,137,164,238,253 |
| E | 4 | 39,56,69,93 | 27,56,94,148 | 34,78,96,158 | 46,78,115,163 |
|  | 5 | 50,103,143,169,182 | 32,57,92,133,166 | 23,55,70,107,179 | 35,54,97,156,185 |
|  | 6 | 40,122,156,176,211,200 | 17,33,53,82,128,185 | 24,59,66,88,126,172 | 38,79,108,123,145,197 |
| F | 4 | 19,62,85,148 | 37,67,118,167 | 43,77,135,176 | 53,71,112,168 |
|  | 5 | 20,54,95,140,216 | 17,47,77,126,183 | 41,59,82,137,191 | 32,51,82,103,175 |
|  | 6 | 24,53,65,86,157,203 | 34,53,69,120,157,195 | 29,57,91,131,168,209 | 20,73,116,136,159,192 |
| G | 4 | 68,149,180,220 | 31,72,106,157 | 25,70,105,169 | 40,76,123,179 |
|  | 5 | 80,103,125,155,211 | 35,59,94,121,155 | 39,66,89,124,183 | 63,80,105,150,207 |
|  | 6 | 55,103,136,163,199,200 | 22,47,74,111,145,180 | 29,59,90,123,159,194 | 26,62,69,100,165,197 |
| H | 4 | 93,116,172,207 | 43,79,106,138 | 64,122,163,211 | 43,87,125,166 |
|  | 5 | 48,96,115,145,154 | 67,113,148,178,204 | 82,139,176,188,204 | 60,115,174,202,224 |
|  | 6 | 49,75,116,170,190,203 | 60,112,138,149,176,200 | 22,104,138,161,177,100 | 53,115,129,152,193,200 |


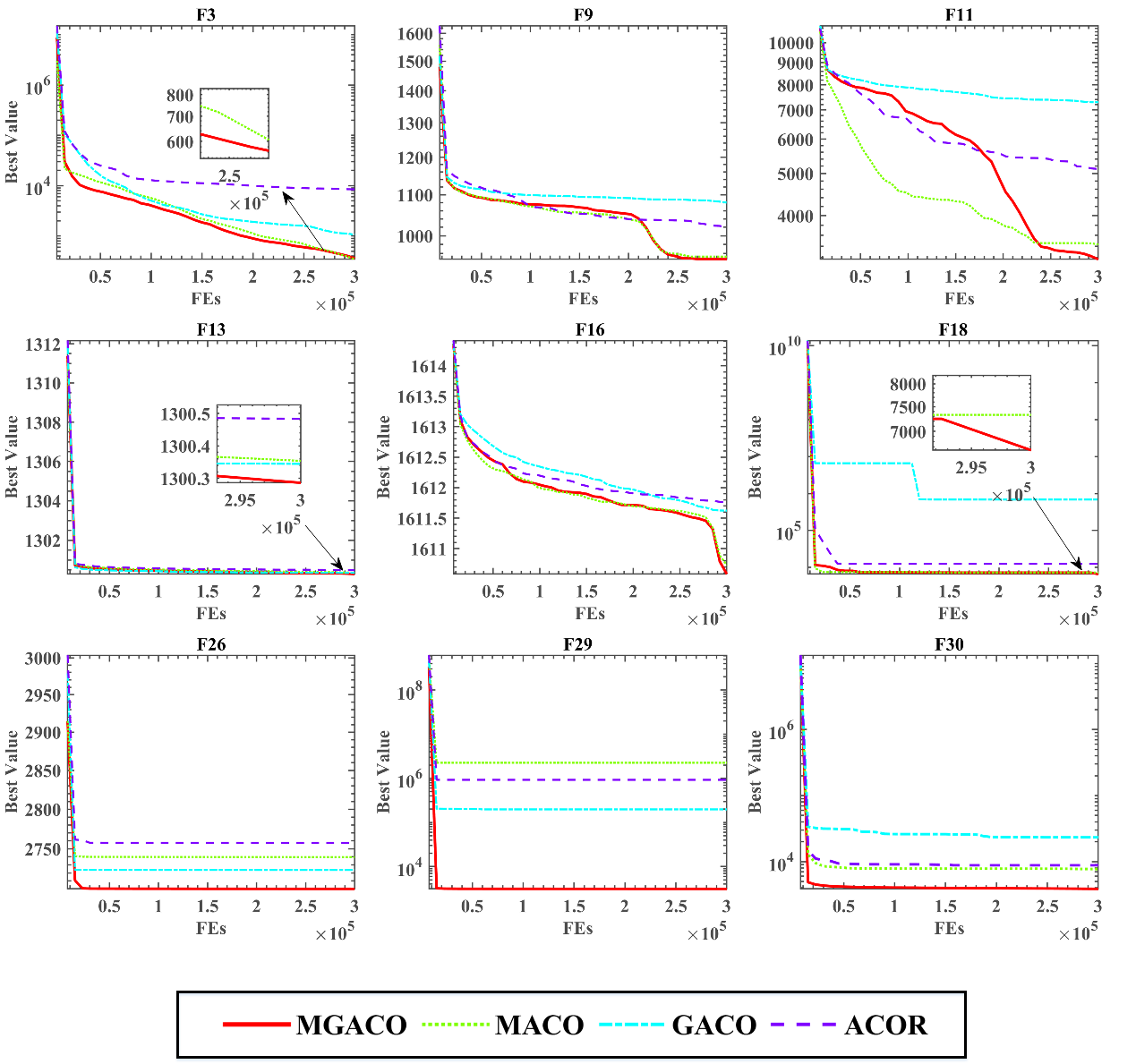


**Fig A 1.** Some convergence curves on benchmark functions


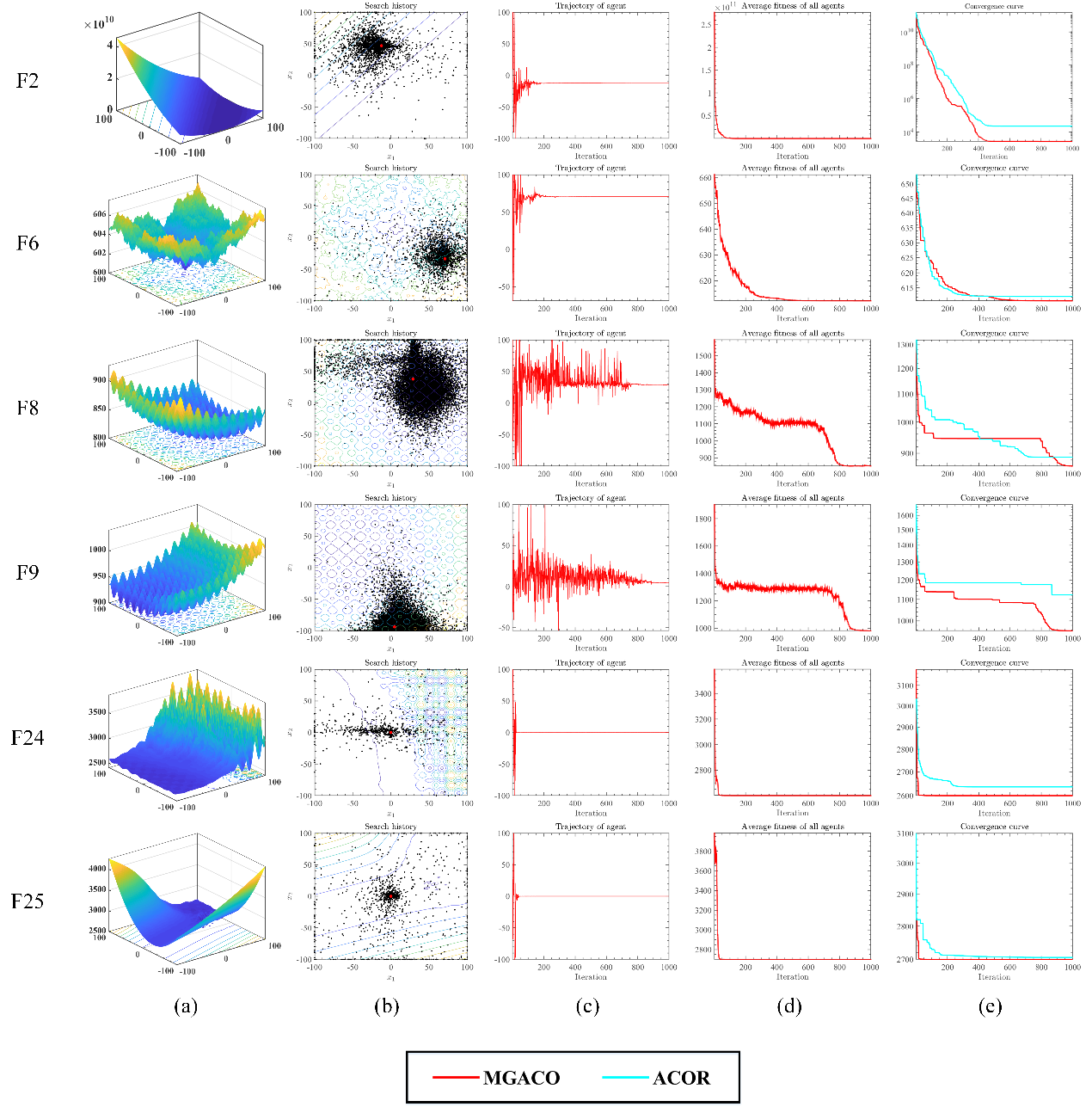


**Fig A 2.** (a) 3D location distribution of MGACO, (b) 2D location distribution of MGACO, (c) Trajectory of MGACO in the first dimension, (d) Average fitness of MGACO, (e) Convergence curves of MGACO and ACOR


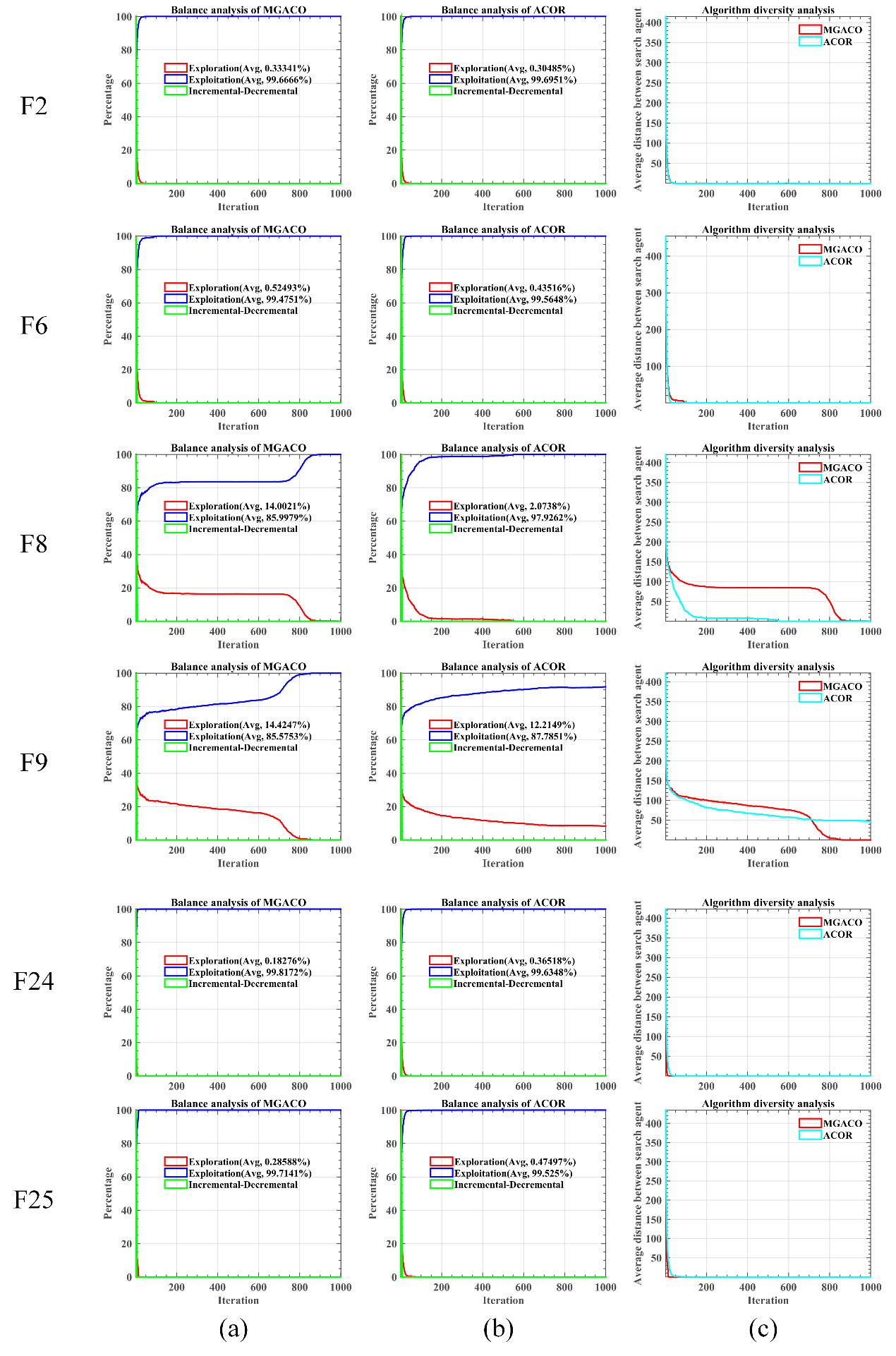


**Fig A 3.** (a) The balance analysis of MGACO, (b) The balance analysis of ACOR, (c) The diversity analysis of MGACO and ACOR


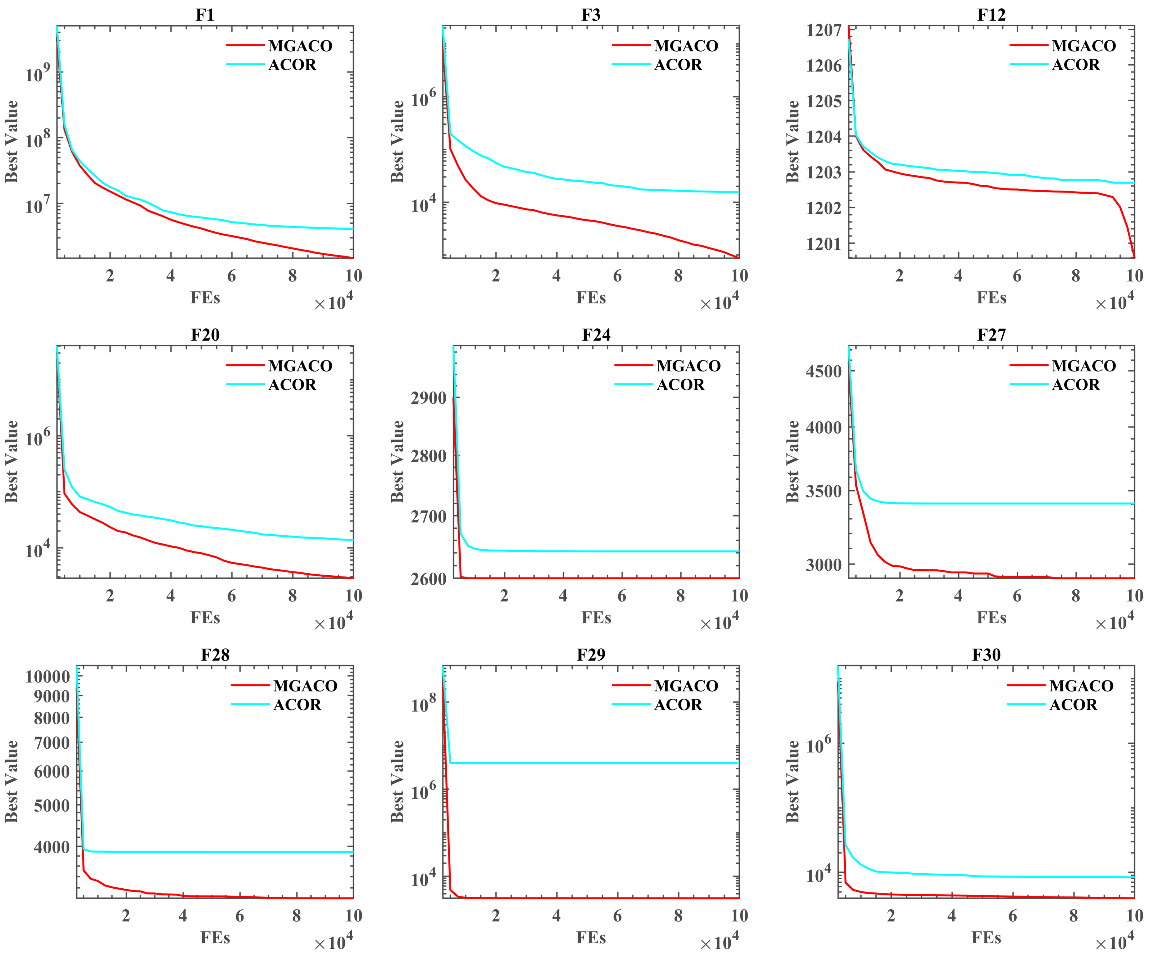


**Fig A 4.** Convergence curves of MGACO and ACOR at dimension 10


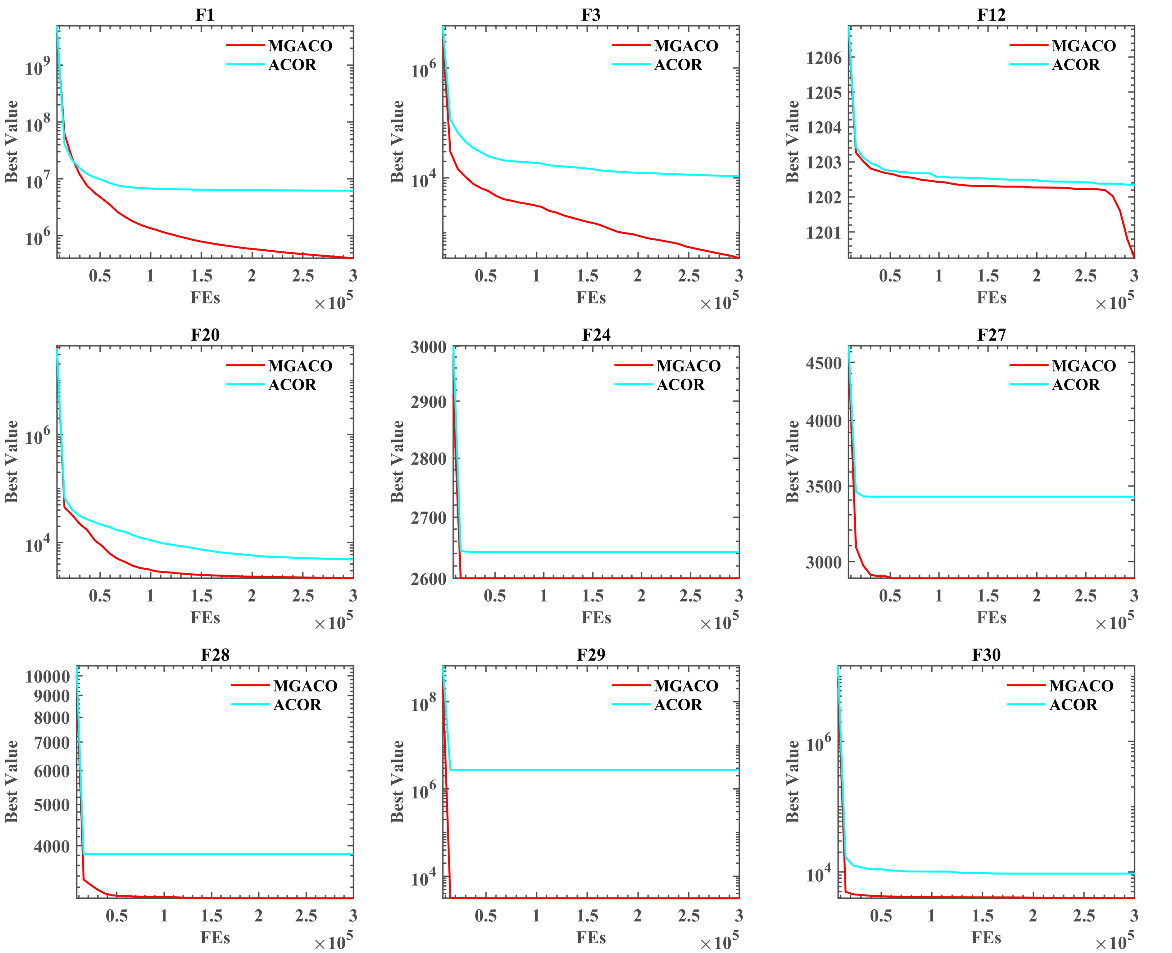


**Fig A 5.** Convergence curves of MGACO and ACOR at dimension 30


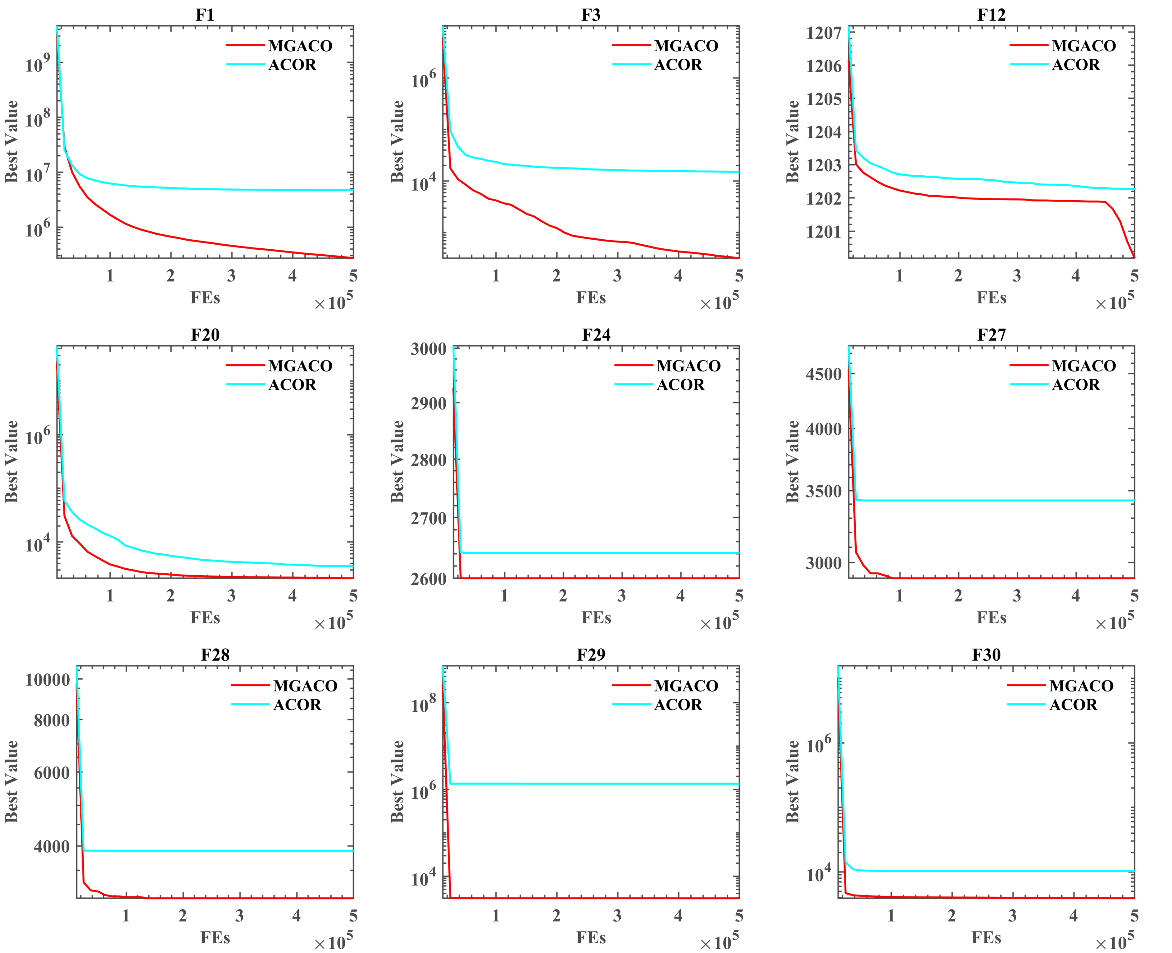


**Fig A 6.** Convergence curves of RCACO and ACOR at dimension 50


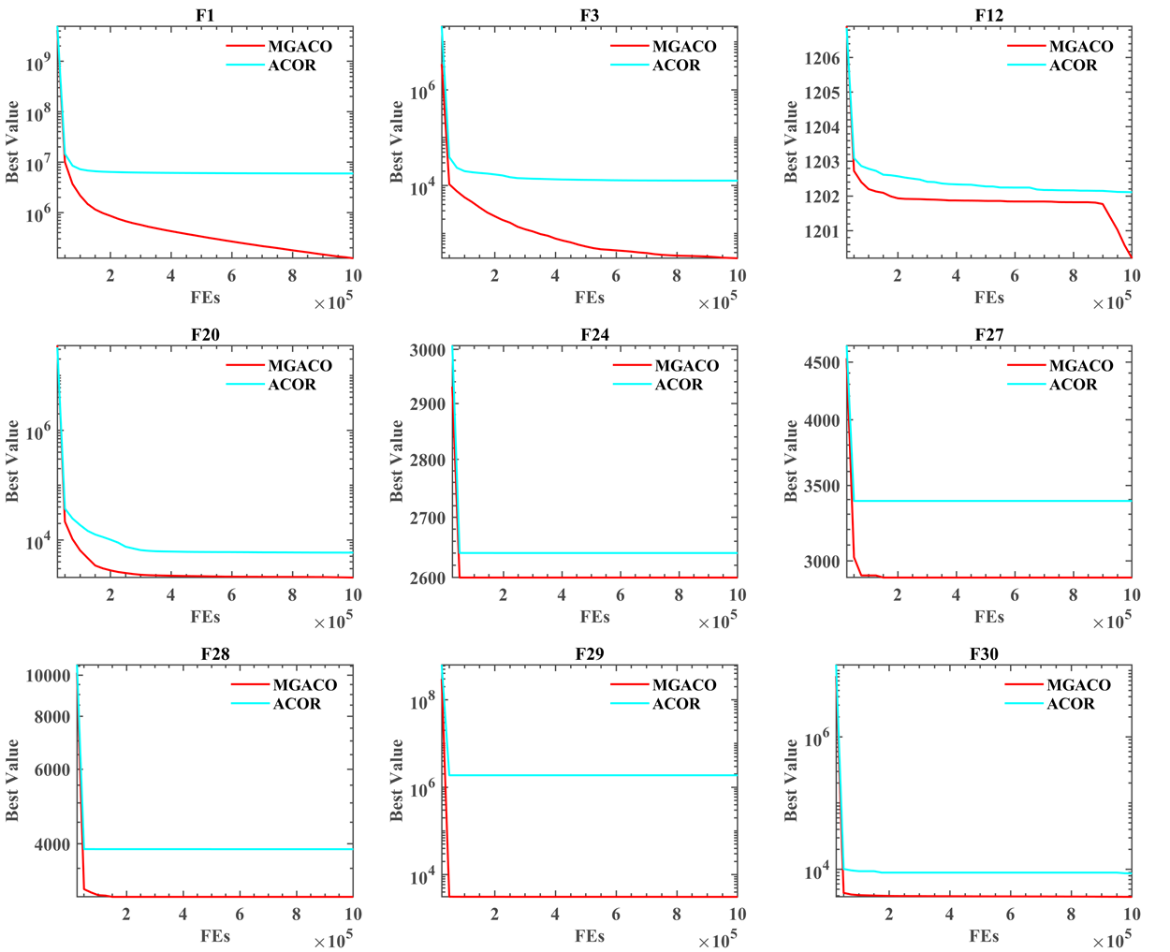


**Fig A 7.** Convergence curves of RCACO and ACOR at dimension 100


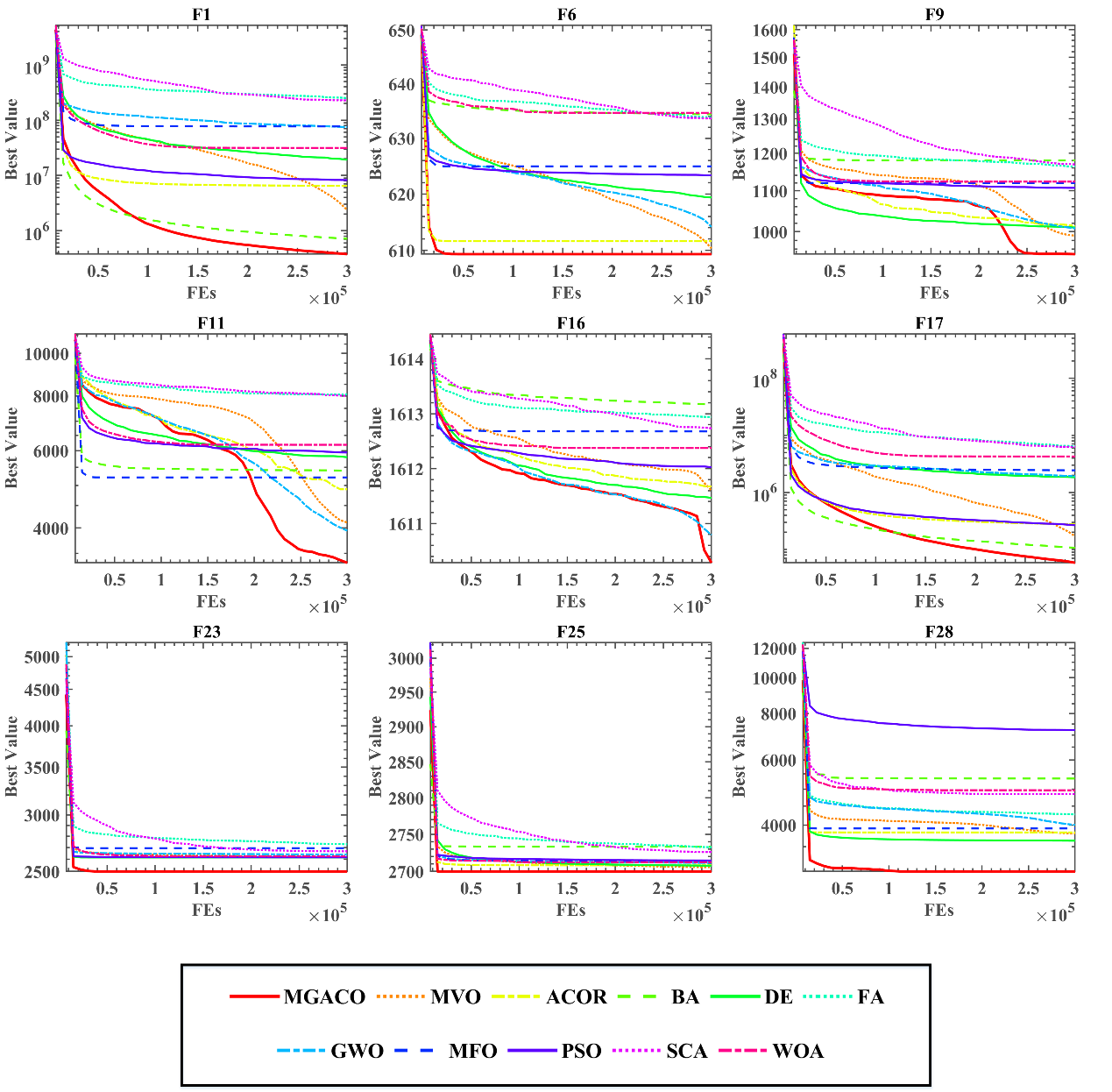


**Fig A 8.** Convergence curves of MGACO and its 10 basic peers on some benchmark functions


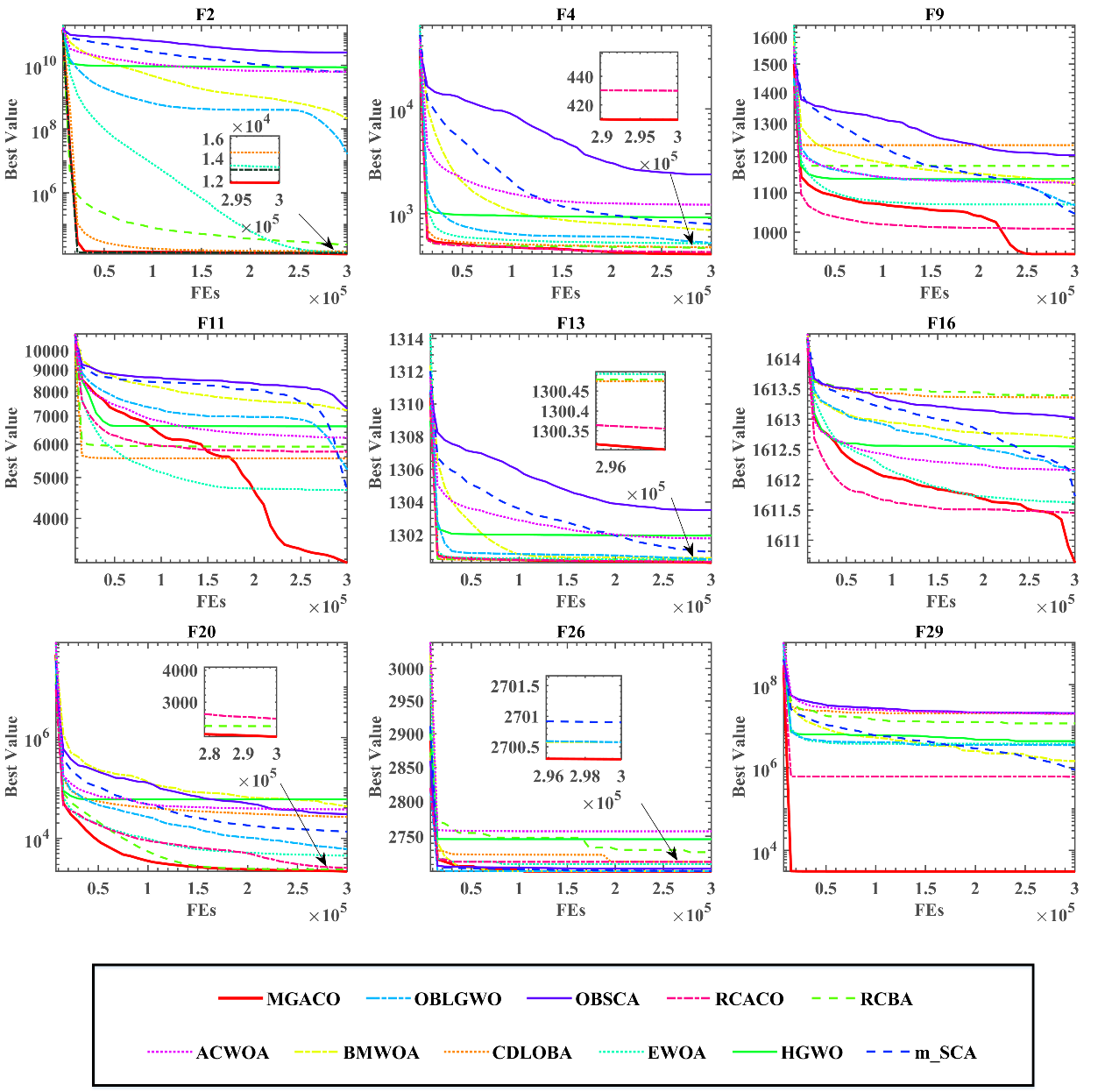


**Fig A 9.** Convergence curves of MGACO and its 10 advanced peers on some benchmark functions


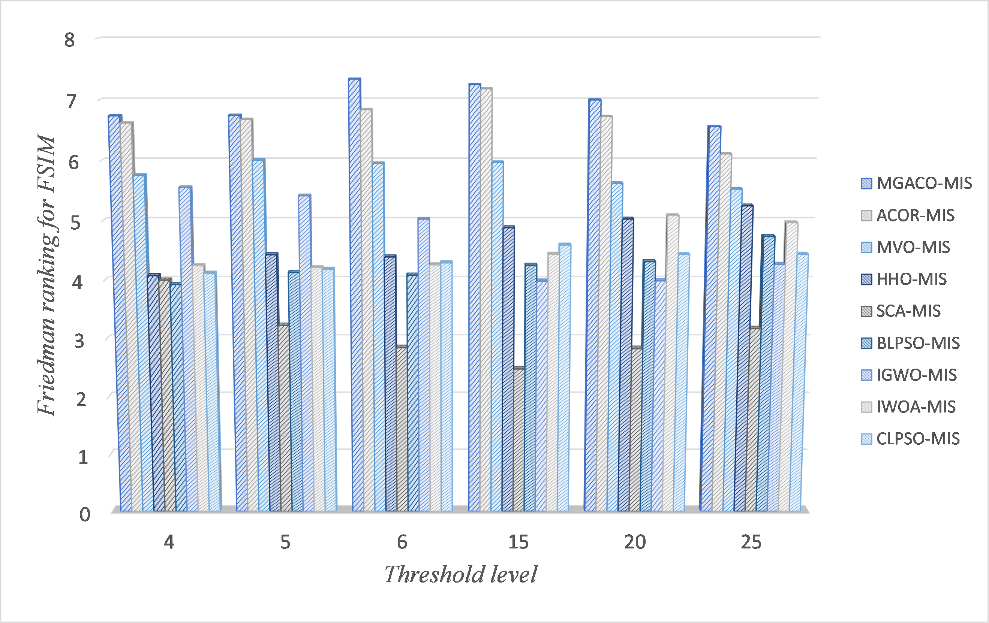


**Fig A 10.** Friedman test results of MGACO-MIS and its peers for FSIM evaluation


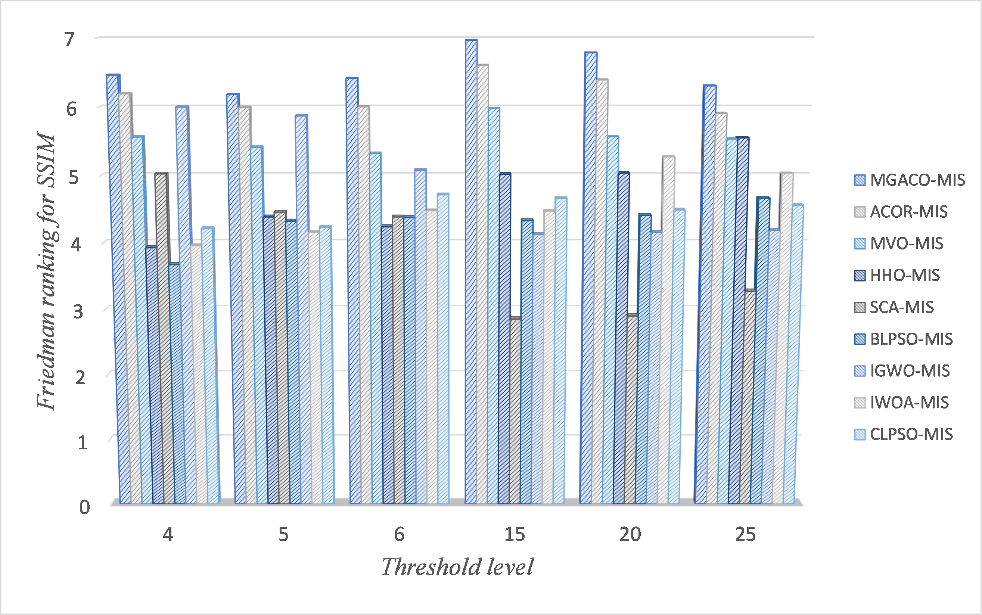


**Fig A 11.** Friedman test results of MGACO-MIS and its peers for SSIM evaluation


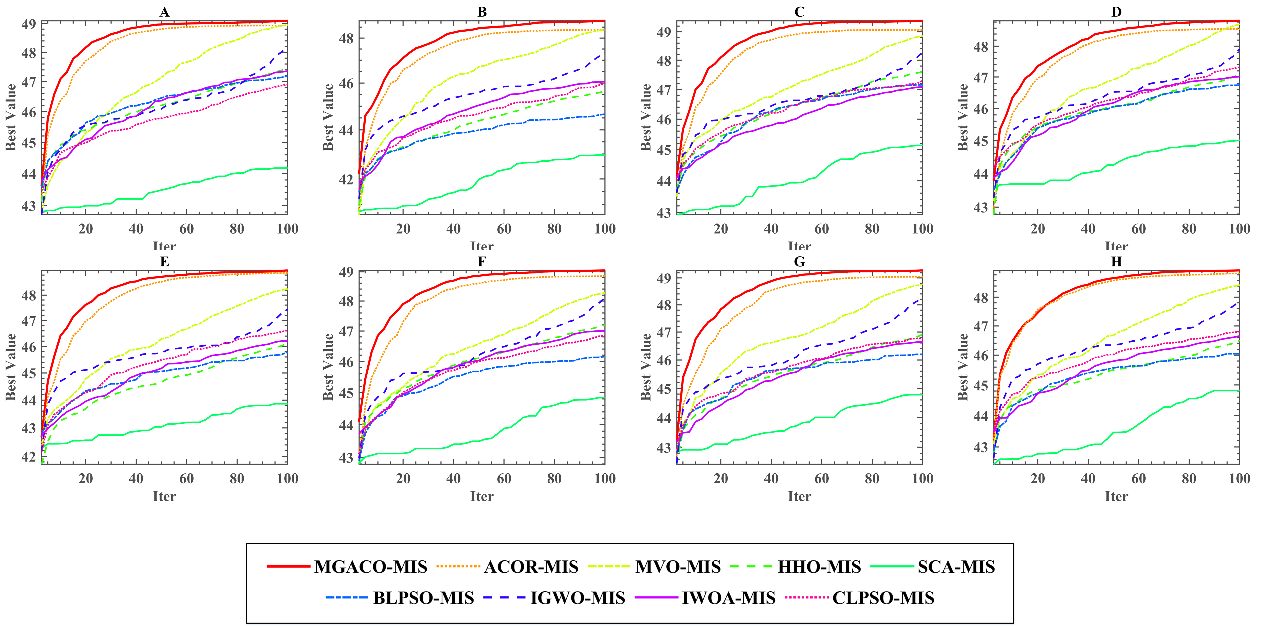


**Fig A 12.** Convergence curves of 2D kapur’s entropy for all methods at threshold level 6


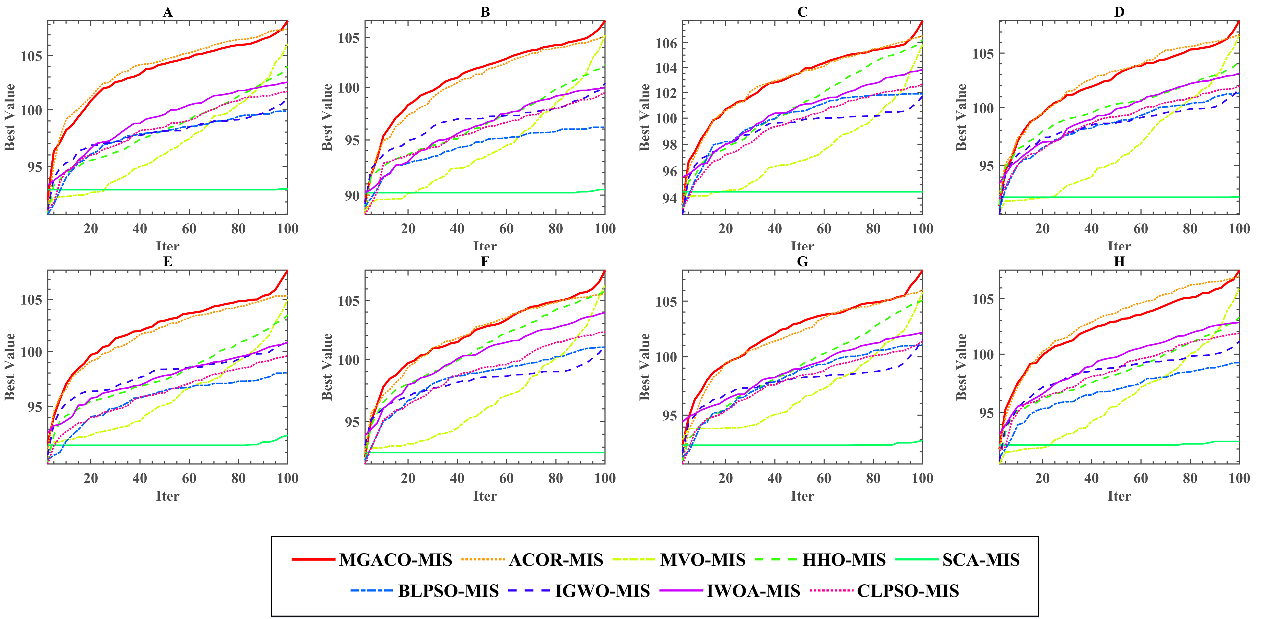


**Fig A 13.** Convergence curves of 2D kapur’s entropy for all methods at threshold level 25


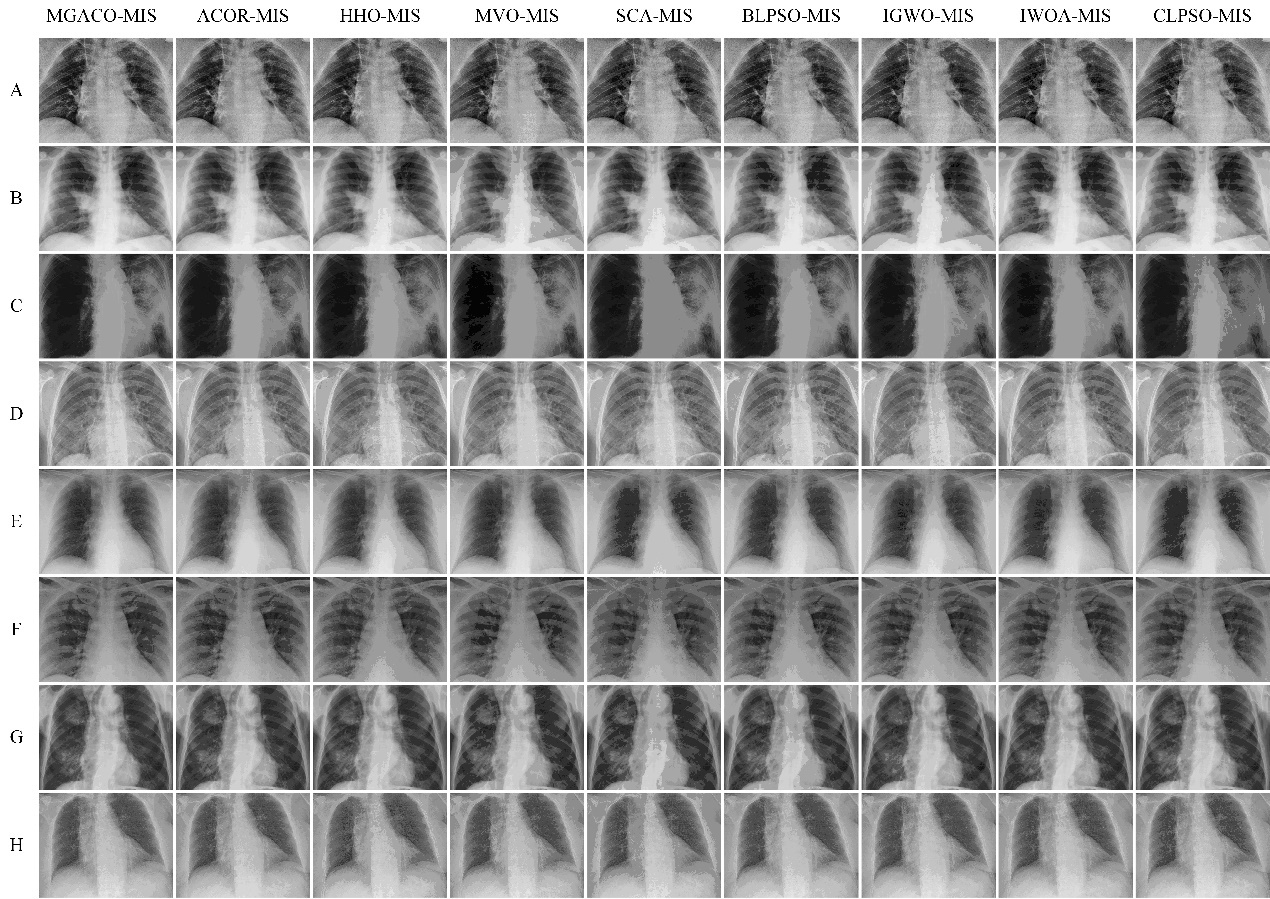


**Fig A 14.** Segmentation results by using 2D kapur’s entropy for all methods at threshold level 25
